# Surface Atomic Arrangements on High-Entropy-Alloy Nanocrystals Govern Alkaline Hydrogen Oxidation via Symmetry-Selected Multi-Atom Ensembles

**DOI:** 10.1021/jacs.6c13325

**Published:** 2026-09-04

**Authors:** Kuan-Fang Lee, Liang-Yu Hou, Yueh-Chun Hsiao, Chia-Ying Wu, Yun-Shan Tsai, Chun-Wei Chang, Jui-Tai Lin, Shang-Cheng Lin, Cheng-Yu Wu, Zong-Ying He, Yi Chen, Shu-Han Lee, Chia-Hsin Wang, Chih-Wen Pao, Kun-Han Lin, Tung-Han Yang

**Affiliations:** † Department of Chemical Engineering, 34881 National Tsing Hua University, Hsinchu 300044, Taiwan; ‡ College of Semiconductor Research, National Tsing Hua University, Hsinchu 300044, Taiwan; § 57815 National Synchrotron Radiation Research Center, Hsinchu 300092, Taiwan; ∥ High Entropy Materials Center, National Tsing Hua University, Hsinchu 300044, Taiwan

## Abstract

High-entropy alloys (HEAs) provide compositionally diverse surfaces for electrocatalysis, yet how their surface atomic arrangements dictate active-site ensembles and catalytic activity remains unclear. Here, we develop a dropwise deposition strategy to synthesize Pd@Pd_0.2_Pt_0.2_Ir_0.2_Ru_0.2_Rh_0.2_ core–shell nanocrystals with comparable shell compositions but distinct surface atomic arrangements, including square {100} facets, hexagonal {111} facets, and twin-interrupted {111} facets. Among them, {111}-enclosed nanooctahedra exhibit the highest alkaline hydrogen oxidation reaction (HOR) activity, delivering a 4.18-fold enhancement over commercial Pt/C at 0.10 V_RHE_. *In situ* synchrotron X-ray absorption spectroscopy and ambient-pressure X-ray photoelectron spectroscopy reveal potential-dependent interactions between Ru and OH*, accompanied by electronic-structure coupling among Pd, Pt, Ir, and Rh during HOR. Density functional theory and climbing-image nudged elastic band calculations show that the hexagonal {111} symmetry optimizes H*/OH* adsorption, H* spillover, and H_2_O formation through symmetry-selected multi-atom ensembles. These elementary processes lower the Volmer-step barrier, supporting a Volmer-influenced pathway. Experimental and computational insights demonstrate that HEA surface atomic arrangements govern alkaline HOR kinetics. Unlike conventional mono- or bimetallic catalysts with limited active-site motifs, the HEA {111} surface provides a distributed ensemble network with varied adsorption strengths, establishing surface atomic arrangement and active multi-atom ensembles as key descriptors for high-performance HEA electrocatalysts.

## Graphical abstract

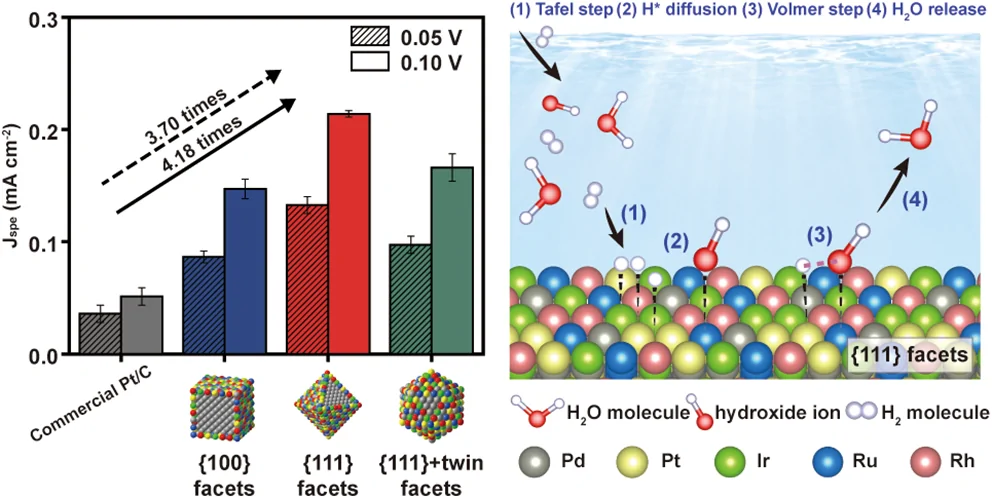

## Introduction

Multicomponent high-entropy-alloy (HEA) nanocrystals have recently attracted considerable attention in catalysis because of their unique combination of compositional tunability and structural complexity.^1–4^ These nanocrystals, which typically contain five or more metallic elements in a single solid solution phase, offer an extremely large compositional space and abundant catalytically active sites.^5–9^ By integrating multiple elements within one nanoscale lattice, HEA nanocrystals generate a vast number of local atomic configurations and electronic environments, giving rise to a rich ensemble of surface sites with tunable adsorption characteristics.^1,4,10,11^ This configurational richness makes HEA nanocrystals fundamentally distinct from conventional mono and bimetallic catalysts and provides a powerful platform for optimizing catalytic activity, selectivity, and durability through rational control of composition, surface structure, and electronic coupling.^5,6,12–14^ At the same time, however, the synthesis of well-defined multicomponent nanocrystals that combine uniform composition with controlled surface atomic arrangements remains highly demanding.

In heterogeneous catalysis, it is well established that surface structure, including the exposed facets and the local coordination of surface atoms, plays a decisive role in controlling adsorption and reaction pathways.^15–17^ On single-component or simple alloy surfaces, low-index and high-index facets can display markedly different binding strengths for relevant adsorbates, including reactants, intermediates, and products, as well as distinct ensembles of adsorption sites (e.g. top, bridge, and hollow) that govern adsorption energetics and facilitate elementary reaction pathways.^18–21^ For HEAs, the situation is even richer. The random mixing of multiple elements generates a wide spectrum of local atomic arrangements and elemental combinations, providing a configurational diversity that can, in principle, be harnessed to optimize complex reaction sequences.^1,4,11,22^ Indeed, HEA-based catalysts have already shown promising performance toward reactions such as hydrogen evolution (HER)^23–28^ and hydrogen oxidation reactions (HOR).^24,27–31^ However, most studies have focused on the role of the overall composition, including the identity and ratio of the mixed elements and average electronic structure descriptors, in determining catalytic performance.^5,14^ In many studies, HEA surfaces are approximated as compositionally averaged planes, while the influence of specific local atomic arrangements and surface symmetries has been much less explored.^5,6,14^ This is perhaps not surprising, because realizing multicomponent HEA nanocrystals that exhibit distinct surface atomic arrangements yet share the same overall composition is greatly challenging. As a consequence, it has been difficult to disentangle the effects of facet exposure and local atomic environments from those of composition, and their individual roles in HEA catalysis therefore remain fundamentally unresolved.

Alkaline exchange membrane fuel cells (AEMFC) are widely regarded as promising technologies for sustainable hydrogen energy conversion.^32–35^ Yet, in stark contrast to acidic media, the HOR in alkaline electrolytes suffers from sluggish kinetics, which severely limits cell efficiency and increases the noble metal loading required at the anode.^32,34–38^ In alkaline media, the HOR is generally described by a Tafel–Volmer or Heyrovsky–Volmer sequence, involving the following elementary steps: (1) Tafel step, H_2_ + 2* → 2H*; (2) Heyrovsky step, H_2_ + OH^–^ + * → H* + H_2_O + e^–^; and (3) Volmer step, H* + OH^–^ → H_2_O + e^–^ + *, where * denotes a surface site and H* represents adsorbed hydrogen.^34,35,39^ The elementary reactions are well-defined, but their relative contributions and the identity of the rate-determining step under alkaline conditions remain under debate, especially because H* can react either with OH^–^ in solution or with adsorbed hydroxyl (OH*; formed by OH^–^ + * → OH* + e^–^) to produce H_2_O.^35,40,41^ The hydrogen binding energy (HBE) theory highlights H* as the key reaction intermediate and correlates HOR activity with the strength of hydrogen adsorption.^34,35,41^ However, it is difficult to construct a comprehensive picture of the reaction solely from HBE, because OH^–^ and OH* explicitly participate in the Heyrovsky and Volmer steps.^35^ The hydroxyl binding energy (OHBE) or bifunctional theory therefore emphasizes the cooperative adsorption of both H* and OH* on neighboring sites.^39,40,42^ Computational simulations and experimental studies have shown that the interplay between the adsorption and desorption of H* and OH*, together with their coverage-dependent competition for surface sites, plays a decisive role in determining alkaline HOR performance.^42–46^

Although platinum group metal (PGM; Pt, Pd, Ir, Ru, and Rh)-based catalysts remain the benchmark for HOR, their activity in alkaline media is still far from ideal, and their high cost motivates the development of new multimetallic catalysts that can deliver both high performance and efficient utilization of noble metals.^39,41^ In light of the mechanistic picture outlined above, it is increasingly clear that alkaline HOR kinetics are governed by the cooperative behavior of multiple adsorption sites, involving not only hydrogen binding but also hydroxyl adsorption and interfacial water activation.^40,41,47,48^ These considerations imply that a single averaged hydrogen adsorption energy is not sufficient to describe alkaline HOR activity and that effective catalyst design must instead aim to simultaneously optimize the binding of both hydrogen and hydroxyl species on the catalyst surface.^40,49^ These insights underscore the likely importance of multi-atom ensembles that can cooperatively bind H* and OH* or promote water activation,^50^ particularly on multicomponent HEA surfaces where a wide variety of local atomic arrangements naturally exists.^29,51^ However, because composition and surface atomic arrangements are often strongly intertwined in HEA nanocrystals, it remains difficult to disentangle their respective contributions.^14,52^ As a consequence, our current understanding of which specific configurations and surface symmetries on HEA surfaces govern alkaline HOR activity, and how they should be engineered, is still limited and fragmented.^53^

In this work, we show that the atomic arrangement of PGM-HEA surfaces exerts decisive control over alkaline HOR activity. To isolate this effect, we construct three PdPtIrRuRh HEA surface structures with carefully matched overall compositions but distinct atomic arrangements: {100} facets that exhibit a square atomic arrangement, {111} facets with a hexagonal atomic arrangement, and {111} facets where the hexagonal atomic arrangement is intersected by twin planes that introduce well-defined mirror symmetry. Electrochemical benchmarking under identical alkaline HOR conditions shows that the {111}-enclosed PGM-HEA surfaces deliver the highest intrinsic activity among the three matched composition surfaces, exhibiting an approximately 3.70-fold enhancement over Pt/C at 0.05 V_RHE_ and 4.18 fold at 0.10 V_RHE_ (measured relative to a reversible hydrogen electrode). These comparisons indicate that an uninterrupted hexagonal atomic arrangement on {111} provides the most favorable surface motif for alkaline HOR, outperforming both the square arrangement on {100} and the twin-interrupted {111} arrangement. Furthermore, by integrating electrochemical measurements, *in situ* synchrotron X-ray absorption spectroscopy (XAS), ambient pressure X-ray photoelectron spectroscopy (AP-XPS), density functional theory (DFT) and climbing-image nudged elastic band (CI-NEB) calculations, we decouple the influence of surface atomic arrangements from compositional effects. This approach reveals how symmetry-selected multi-atom ensembles regulate alkaline HOR kinetics. Our experimental and computational results reveal that well-defined surface symmetries and multi-atom ensembles cooperatively establish an optimal adsorption landscape for H* and OH*, and reduce the kinetic barriers of key elementary steps, including H* spillover and H_2_O formation, thereby enabling markedly enhanced alkaline HOR activity. These findings establish a direct link between atomic arrangements on HEA surfaces and alkaline HOR kinetics and provide general design principles for engineering multimetallic surfaces for catalysis.

## Results and Discussion

### Tailored Surface Atomic Arrangements on HEA Nanocrystals

To obtain these three surface atomic arrangements, we epitaxially grew PdPtIrRuRh HEA atomic layers on preformed Pd nanocrystal seeds using a modified polyol method (Figure 1).^54^ Three types of Pd seeds served as templates: nanocubes enclosed by {100} facets, nanooctahedra enclosed by {111} facets, and nanoicosahedra enclosed by twinned {111} facets (Figure S1). Each Pd seed dispersion was mixed with ethylene glycol (EG) containing l-ascorbic acid (AA) as a reducing agent, poly­(vinylpyrrolidone) (PVP) as a colloidal stabilizer, and KBr as a surface modifier that slows the reduction kinetics and favors epitaxial growth on the seed surface. The suspension was heated to 195 °C under vigorous stirring, followed by dropwise injection of an EG solution containing equimolar Pd­(II), Pt­(II), Ir­(IV), Ru­(III), and Rh­(III) precursors at a slow rate of 0.6–0.8 mL h^–1^. The slow and continuous precursor feeding promotes layer-by-layer atomic deposition on the Pd surfaces while suppressing homogeneous nucleation, yielding conformal Pd_0.2_Pt_0.2_Ir_0.2_Ru_0.2_Rh_0.2_ shells that inherit the crystal facets of the underlying seeds.^55^ After completion of the precursor addition, the reaction mixture was maintained at 195 °C for an additional 2 h to ensure complete reduction and alloying, affording Pd@Pd_0.2_Pt_0.2_Ir_0.2_Ru_0.2_Rh_0.2_ core–shell nanocubes with {100} facets, nanooctahedra with {111} facets, and nanoicosahedra with twinned {111} facets. Unlike one-shot injection, which can produce a rapid increase in precursor concentration and promote sequential reduction, compositional gradients, or homogeneous nucleation, the slow and continuous precursor feeding maintains low and relatively stable precursor concentrations, thereby minimizing differences in the reduction kinetics of the individual metal precursors and promoting their concurrent deposition onto the Pd seeds.^56,57^ This kinetically regulated growth mode facilitates the formation of compositionally mixed HEA shells. In contrast to ultrafast nonequilibrium HEA synthesis approaches, such as carbothermal shock synthesis^1^ and laser scanning ablation,^58^ which rely on rapid heating and quenching to kinetically trap multiple elements, our strategy achieves multielement mixing through controlled precursor delivery under steady-state growth conditions.

**Figure 1:**
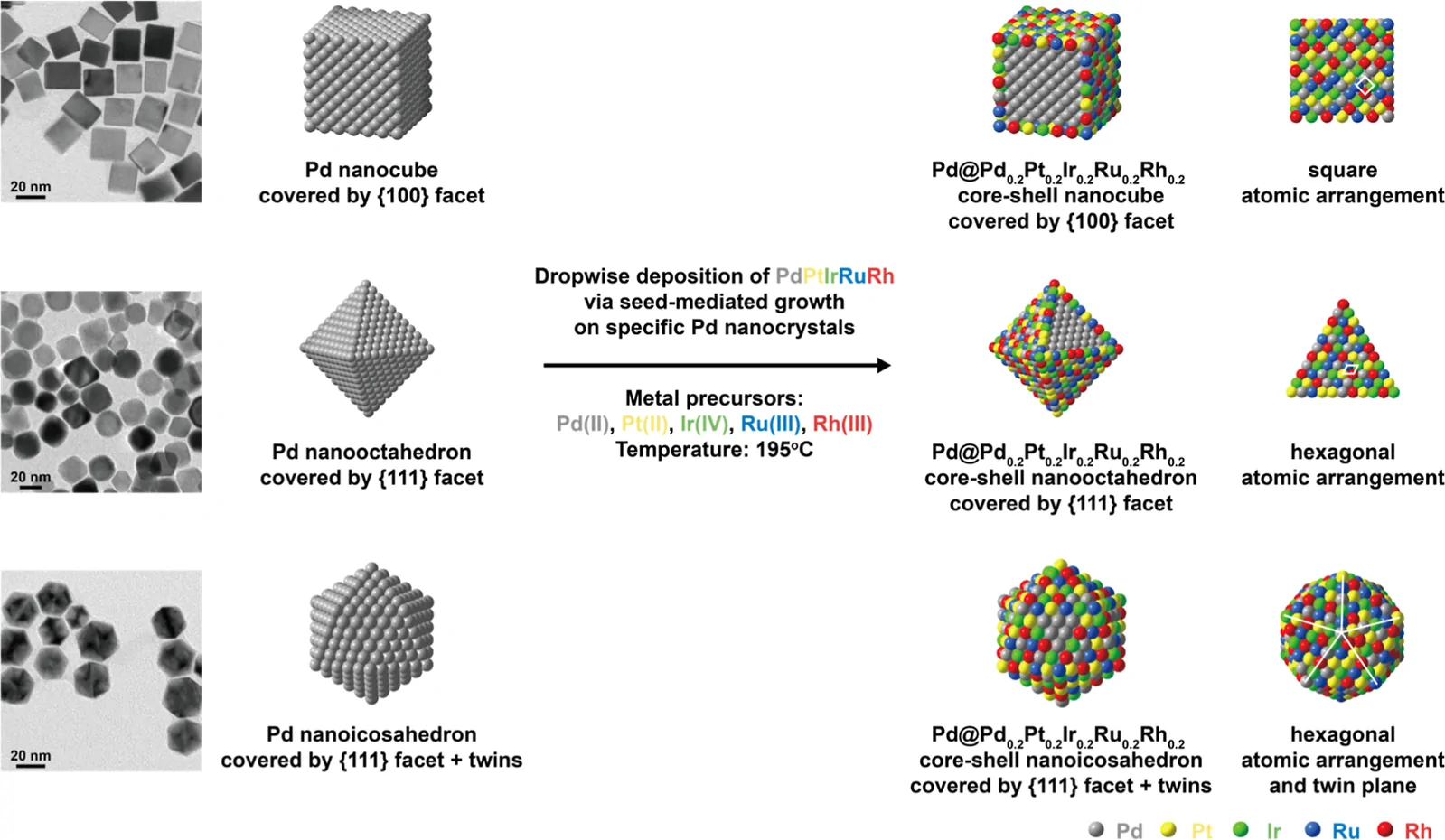
Schematic illustration of the dropwise seed-mediated growth of quinary Pd_0.2_Pt_0.2_Ir_0.2_Ru_0.2_Rh_0.2_ HEA shells on shape-controlled Pd nanocrystal seeds, together with representative TEM images of the corresponding Pd nanocubes, nanooctahedra, and nanoicosahedra. The resulting core–shell nanocrystals inherit the seed morphologies and expose {100}, {111}, and twin-interrupted {111} surface atomic arrangements, respectively.

Figure 2a shows a low-magnification transmission electron microscopy (TEM) image of uniform Pd@Pd_0.2_Pt_0.2_Ir_0.2_Ru_0.2_Rh_0.2_ core–shell nanocubes with smooth surfaces, while the corresponding high-angle annular dark-field scanning transmission electron microscopy (HAADF-STEM) image confirms a conformal PdPtIrRuRh shell surrounding the Pd core (Figure 2b). The atomic-resolution HAADF-STEM image in Figure 2c resolves well-resolved atomic columns with an interplanar spacing of about 0.20 nm, which can be indexed to the (002) planes of a face-centered cubic (FCC) structure viewed along the [100] direction, and the fast Fourier transform (FFT) pattern in Figure 2d further confirms the single-crystalline nature of the nanocubes. These observations are consistent with X-ray diffraction (XRD) results, which validate that the Pd@Pd_0.2_Pt_0.2_Ir_0.2_Ru_0.2_Rh_0.2_ core–shell nanocubes crystallize in a FCC structure (Figure S2). For comparison, the XRD patterns of the corresponding Pd cubic, octahedral, and icosahedral seeds are also provided. Based on the HAADF-STEM images, we estimated the HEA-shell thickness by comparing the theoretical HEA-to-Pd ratios derived from the structural model with the experimentally measured ratio obtained from inductively coupled plasma optical emission spectrometry (ICP-OES) (Table S1; see the Supporting Information for details). This analysis suggests that the HEA shells are approximately four to five atomic layers thick. Elemental mapping and EDS analyses show uniform Pd, Pt, Ir, Ru, and Rh distributions without obvious segregation, while ICP-OES confirms a near-equimolar shell composition (Figure 2e–h and Table S2).

**Figure 2:**
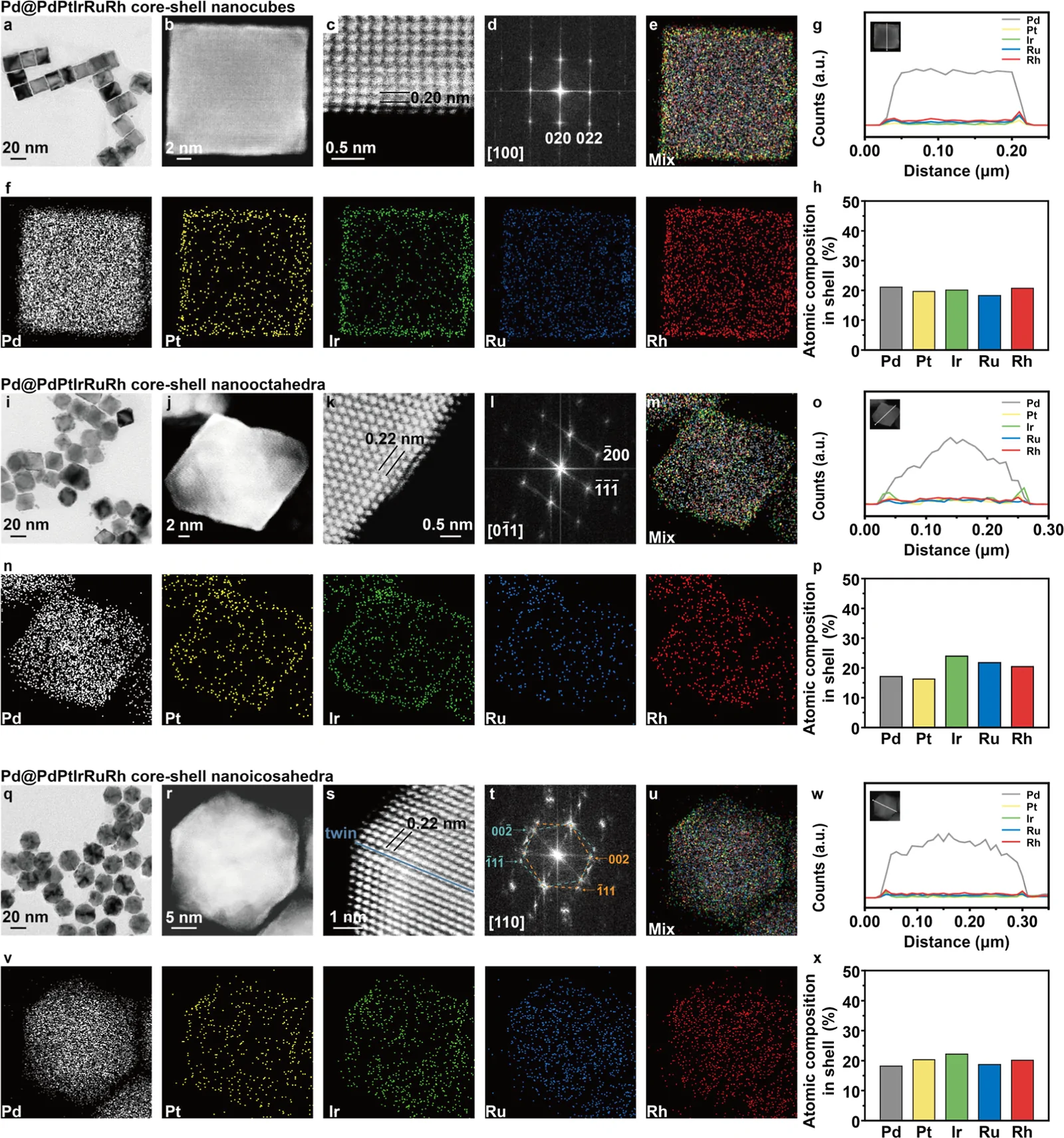
Structural and compositional characterization of Pd@Pd_0.2_Pt_0.2_Ir_0.2_Ru_0.2_Rh_0.2_ core–shell nanocrystals. (a–h) Core–shell nanocubes: (a) TEM image, (b–d) HAADF-STEM images and FFT analyses, (e–g) EDS mapping and line-scan, and (h) ICP-OES-derived shell composition. (i–p) Core–shell nanooctahedra: (i) TEM image, (j–l) HAADF-STEM images and FFT analyses, (m–o) EDS mapping and line-scan, and (p) ICP-OES-derived shell composition. (q–x) Core–shell nanoicosahedra: (q) TEM image, (r–t) HAADF-STEM images and FFT analyses, (u–w) EDS mapping and line-scan, and (x) ICP-OES-derived shell composition.

The same characterization was performed for the Pd@Pd_0.2_Pt_0.2_Ir_0.2_Ru_0.2_Rh_0.2_ core–shell nanooctahedra. TEM and HAADF-STEM images confirm the octahedral morphology and a brighter PdPtIrRuRh shell surrounding the Pd core (Figure 2i,j). Atomic-resolution HAADF-STEM in Figure 2k reveals well-resolved atomic columns with a spacing about 0.22 nm, which can be assigned to the (111) planes of the FCC structure and agrees well with XRD results (Figure S2). The FFT pattern in Figure 2l further confirms that the octahedra are single-crystalline and mainly enclosed by {111} facets. The thickness of the HEA shells is again estimated to correspond to roughly four to five atomic layers.

EDS mapping and line–scan profiles show uniform Pd, Pt, Ir, Ru, and Rh distributions, while ICP-OES confirms a near-equimolar shell composition (Figure 2m–p and Table S2).

For the Pd@Pd_0.2_Pt_0.2_Ir_0.2_Ru_0.2_Rh_0.2_ core–shell nanoicosahedra, TEM and HAADF-STEM images confirm the twinned nanoicosahedral morphology and a brighter PdPtIrRuRh shell surrounding the Pd core (Figure 2q,r). Interestingly, the PdPtIrRuRh shell appears thicker along the twin planes, likely due to their higher surface energy, which favors nucleation and shell growth. The atomic-resolution HAADF-STEM image in Figure 2s resolves well-resolved atomic columns with a spacing of about 0.22 nm (assigned to the FCC (111) planes) together with clear twin planes, indicating that the HEA shells inherit the multiply twinned FCC motif of the Pd icosahedral seeds. This observation is consistent with the XRD patterns in Figure S2, which display weak and broadened reflections, as expected for nanosized multiply twinned domains. The corresponding FFT pattern in Figure 2t exhibits split diffraction spots that are consistent with the presence of twin domains. The corresponding EDS maps and ICP-OES analysis in Figure 2u–x and Table S2 further confirm that the PdPtIrRuRh shells form a homogeneous solid solution. Together, these results demonstrate that the epitaxial growth strategy enables precise construction of PGM-HEA shells with three distinct surface atomic arrangements, namely square {100}, hexagonal {111}, and twinned {111} facets, at essentially the same overall composition. Additional EDS analyses of independently examined nanocubes, nanooctahedra, and nanoicosahedra show consistently overlapping distributions of Pd, Pt, Ir, Ru, and Rh without obvious elemental segregation (Figure S3). A minor population of self-nucleated clusters was also observed and quantitatively analyzed using representative nanooctahedral samples (Figures S4–S6). These clusters contribute only a small fraction of the total estimated surface area and particle volume, and predominantly retain an FCC multimetallic structure containing all five constituent elements.

To examine the influence of precursor composition on shell growth, we synthesized a series of Pd@multimetallic nanooctahedra under the same conditions used for the quinary HEA nanooctahedra, except for the metal precursor composition (Figure S7). Pd@Pt_0.33_Ir_0.33_Ru_0.33_ nanooctahedra exhibit relatively conformal shell growth (Figure S7a), whereas Pd@Ir_0.5_Ru_0.5_ nanooctahedra show more pronounced island-like deposition (Figure S7b). Pd@Pt_0.5_Ru_0.5_ and Pd@Pt nanooctahedra exhibit discernible shell growth accompanied by some homogeneous nucleation (Figure S7c,d), while Pd@Ru nanooctahedra display rough and nonuniform surfaces (Figure S7e). These observations indicate that precursor composition influences shell-growth behavior under otherwise identical reaction conditions, likely through differences in precursor reduction kinetics, metal–seed interactions, and structural compatibility between the deposited metals and the FCC Pd substrate. These representative TEM observations suggest that precursor composition influences shell-growth behavior, while a more systematic investigation will be needed to establish a general relationship between compositional complexity and epitaxial growth in multimetallic systems.

### Atomically Mixed Coordination Environments on HEA Surfaces

To elucidate the surface chemical states of the representative Pd@Pd_0.2_Pt_0.2_Ir_0.2_Ru_0.2_Rh_0.2_ core–shell nanooctahedra, high-resolution XPS spectra were collected for all five PGM elements (Figure 3a–e), and the peaks were deconvoluted using the fitting parameters summarized in Table S3. These results show that Pd, Pt, Ir, Ru, and Rh are all predominantly present in metallic states, with only minor oxidized contributions detected at higher binding energies (Figure 3a–e and Table S3). These weak oxidized components are attributed to slight surface oxidation upon air exposure, while the HEA shell remains largely metallic.

**Figure 3:**
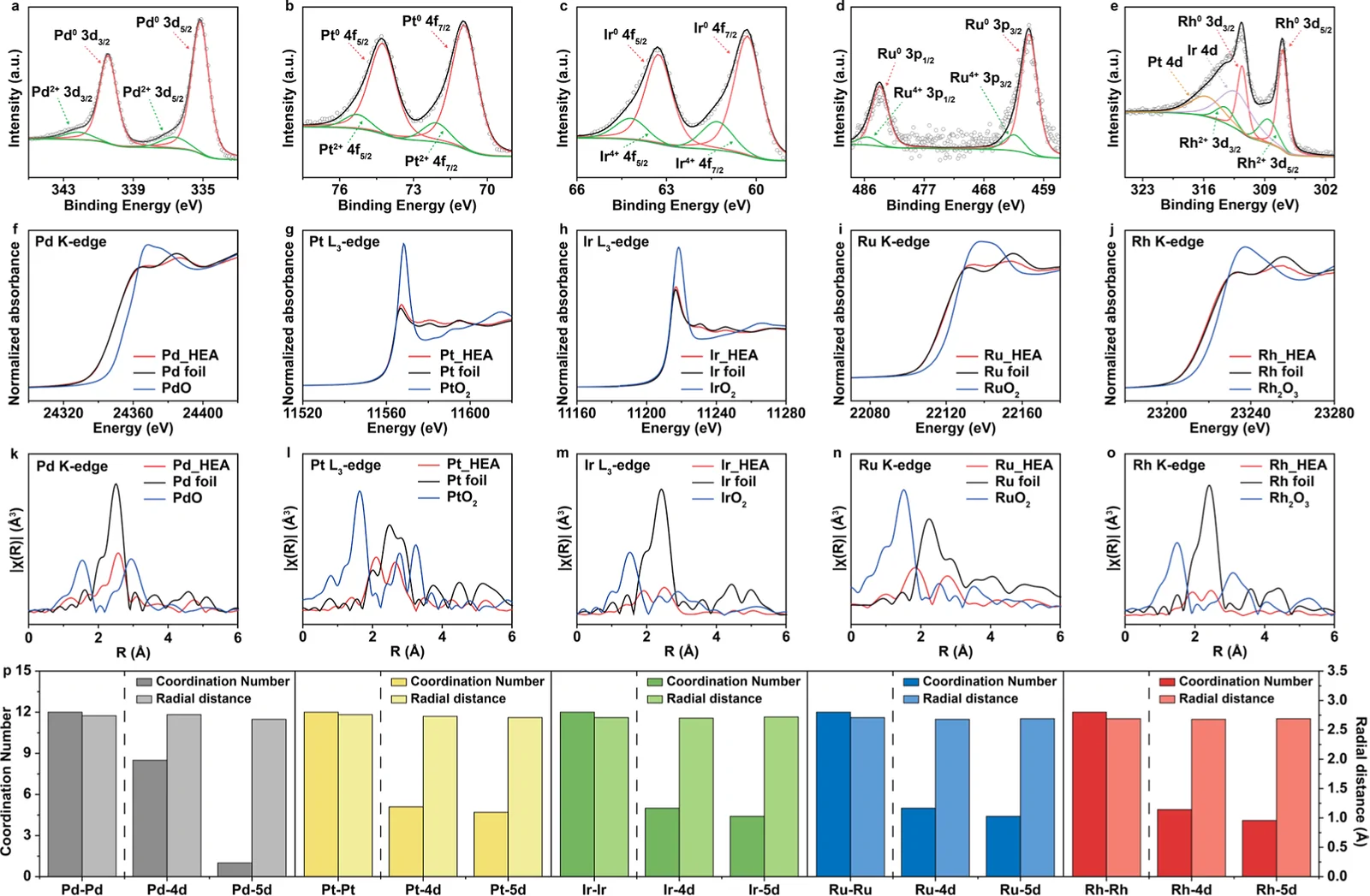
Surface chemical states and coordination environments of Pd@Pd_0.2_Pt_0.2_Ir_0.2_Ru_0.2_Rh_0.2_ core–shell nanooctahedra. (a–e) XPS spectra of Pd@Pd_0.2_Pt_0.2_Ir_0.2_Ru_0.2_Rh_0.2_ core–shell nanooctahedra for (a) Pd 3d, (b) Pt 4f, (c) Ir 4f, (d) Ru 3p, and (e) Rh 3d. (f–j) XANES spectra of Pd@Pd_0.2_Pt_0.2_Ir_0.2_Ru_0.2_Rh_0.2_ core–shell nanooctahedra and the corresponding metallic foils and oxidation states at (f) Pd K-edge, (g) Pt L_3_-edge, (h) Ir L_3_-edge, (i) Ru K-edge, and (j) Rh K-edge. (k–o) FT-EXAFS spectra of Pd@Pd_0.2_Pt_0.2_Ir_0.2_Ru_0.2_Rh_0.2_ core–shell nanooctahedra and the corresponding metallic foils and oxidation states at (k) Pd K-edge, (l) Pt L_3_-edge, (m) Ir L_3_-edge, (n) Ru K-edge, and (o) Rh K-edge. (p) Element-specific coordination parameters derived from FT-EXAFS fitting for the pure metal foils and Pd@Pd_0.2_Pt_0.2_Ir_0.2_Ru_0.2_Rh_0.2_ core–shell nanooctahedra. X–X represents the fitted coordination parameters of the corresponding pure metal foil, whereas the HEA sample was fitted using the X-4d and X-5d scattering paths.

Synchrotron XAS was further carried out to probe the local coordination environments of the representative Pd@Pd_0.2_Pt_0.2_Ir_0.2_Ru_0.2_Rh_0.2_ core–shell nanooctahedra, using the corresponding metallic foils and oxides as standards. The X-ray absorption near-edge structure (XANES) spectra at the Pd K, Pt L_3_, Ir L_3_, Ru K and Rh K edges (Figure 3f–j) lie between those of the metal foils and their oxides and are generally closer to the metallic references, indicating that the PGM elements remain largely in metallic states. At the same time, subtle shifts in edge position and changes in white line intensity relative to the foils may indicate a redistribution of valence electrons and modified orbital hybridization within the Pd_0.2_Pt_0.2_Ir_0.2_Ru_0.2_Rh_0.2_ shell. To clarify the atomic-level mixing in the HEA shell, Fourier-transformed extended X-ray absorption fine structure (FT-EXAFS) spectra were analyzed for all five elements (Figures 3k–o, S8, and S9), and quantitative fittings were performed as summarized in Table 1. For Pd, Pt, Ir, Ru and Rh, the magnitude of the FT-EXAFS shows characteristic alloy-like doublet peaks in the range R ≈ 1.7–2.8 Å, distinct from the single metal–metal and metal–oxygen shells observed in the corresponding foils and oxides. These features arise from multiple metal–metal scattering paths involving different neighboring elements and are consistent with extensive atomic mixing in the shell.^59–61^

**Table 1:** Structural Parameters Extracted From the FT-EXAFS Fitting for Pd@Pd_0.2_Pt_0.2_Ir_0.2_Ru_0.2_Rh_0.2_ Core–shell Nanooctahedra and the Corresponding Metallic Foils^a^ ^,^ ^b^

element	sample	bond	CN	R (Å)	σ^2^ (Å^2^)	*S*_0_^2^	Δ*E*_0_ (eV)	R-factor
Pd	Pd foil	Pd–Pd	12.00	2.74	0.004	0.65	–0.42	0.010
	Pd@Pd_0.2_Pt_0.2_Ir_0.2_Ru_0.2_Rh_0.2_ core–shell nanooctahedra	Pd-4d	8.5 ± 0.9	2.76 ± 0.01	0.008			
		Pd-5d	1.0 ± 0.1	2.68 ± 0.02	0.009			
Pt	Pt foil	Pt–Pt	12.00	2.76	0.003	0.71	8.00	0.025
	Pd@Pd_0.2_Pt_0.2_Ir_0.2_Ru_0.2_Rh_0.2_ core–shell nanooctahedra	Pt-5d	4.7 ± 0.4	2.73 ± 0.01	0.003			
		Pt-4d	5.1 ± 0.7	2.71 ± 0.01	0.003			
Ir	Ir foil	Ir–Ir	12.00	2.71	0.003	0.75	8.18	0.031
	Pd@Pd_0.2_Pt_0.2_Ir_0.2_Ru_0.2_Rh_0.2_ core–shell nanooctahedra	Ir-5d	4.4 ± 1.7	2.72 ± 0.03	0.006			
		Ir-4d	5.0 ± 0.8	2.70 ± 0.01	0.003			
Ru	Ru foil	Ru–Ru	12.00	2.71	0.002	0.64	1.47	0.013
	Pd@Pd_0.2_Pt_0.2_Ir_0.2_Ru_0.2_Rh_0.2_ core–shell nanooctahedra	Ru-4d	5.0 ± 1.9	2.68 ± 0.04	0.004			
		Ru-5d	4.4 ± 1.8	2.69 ± 0.08	0.003			
Rh	Rh foil	Rh–Rh	12.00	2.69	0.003	0.68	1.32	0.010
	Pd@Pd_0.2_Pt_0.2_Ir_0.2_Ru_0.2_Rh_0.2_ core–shell nanooctahedra	Rh-4d	4.9 ± 1.2	2.68 ± 0.05	0.004			
		Rh-5d	4.1 ± 1.6	2.69 ± 0.07	0.003			

a FT-EXAFS fitting analyses were conducted using the Artemis software with k^2^-weighted data. The FT-EXAFS fitting was performed over element-specific k ranges of 3–10 Å^–1^ (Pd), 3–11.5 Å^–1^ (Pt and Ru), 3–8.8 Å^–1^ (Ir), and 3–11 Å^–1^ (Rh). The R-space fitting windows were set to 1.6–3.1 Å (Pd), 1.6–3.0 Å (Pt), 1.4–3.2 Å (Ir), and 1.5–3.0 Å (Ru and Rh) in both the Pd@Pd_0.2_Pt_0.2_Ir_0.2_Ru_0.2_Rh_0.2_ core-shell nanooctahedra and the corresponding metallic foils.

b CN: Coordination numbers; R: bond distance; σ^2^: mean square relative displacement (Debye-Waller factor); S_0_^2^: amplitude reduction factor; Δ*E*_0_: edge energy shift; R-factor: goodness of fit.

In the fitting model, we took advantage of the similar backscattering characteristics of the 4d elements (Pd, Ru, and Rh) and the 5d elements (Pt and Ir) and grouped the paths into M-4d and M-5d contributions. The extracted coordination numbers, bond distances, mean square relative displacements σ^2^, amplitude reduction factors S_0_^2^, energy shifts Δ*E*_0_, and R factors are summarized in Figure 3p and Table 1, with fitting uncertainties obtained directly from the Artemis covariance matrix. For Pt, Ir, Ru, and Rh, the coordination numbers toward 4d and 5d neighbors are 5.1 ± 0.7/4.7 ± 0.4, 5.0/4.4, 5.0/4.4, and 4.9/4.1, respectively, demonstrating mixed 4d/5d coordination around each element. The Pd EXAFS signal inevitably contains contributions from both the Pd core and the Pd atoms in the HEA shell, which may explain the slight deviations between Pd@Pd_0.2_Pt_0.2_Ir_0.2_Ru_0.2_Rh_0.2_ and Pd foil. Even so, the pronounced mixed 4d/5d coordination for Pt, Ir, Ru, and Rh provides clear evidence for an atomically mixed HEA phase rather than elemental segregation. These results (Figure 3p and Table 1) show that each PGM atom in the Pd@Pd_0.2_Pt_0.2_Ir_0.2_Ru_0.2_Rh_0.2_ core–shell nanooctahedra is coordinated by a heterogeneous mix of 4d and 5d neighbors, producing atomically mixed HEA surface environments that differ from those of the corresponding pure metal foils. Together with the EDS analysis of spatial elemental distributions and the ICP-OES quantification of shell compositions, the XAS results confirm extensive multielement mixing in the HEA shells from the nanoscale compositional distribution to the local atomic coordination environment.

### Alkaline HOR Electrocatalytic Performance Governed by HEA Surface Atomic Arrangements

The alkaline HOR activities of Pd@Pd_0.2_Pt_0.2_Ir_0.2_Ru_0.2_Rh_0.2_ core–shell nanocubes, nanooctahedra, and nanoicosahedra were evaluated and benchmarked against commercial Pt/C (Figure 4). Measurements were carried out in a three-electrode configuration using H_2_-saturated 0.1 M KOH at a rotation speed of 1600 rpm, where the emergence of pronounced anodic currents at potentials positive of 0 V_RHE_ is characteristic of H_2_ oxidation. In Figure 4a, the linear sweep voltammetry (LSV) curves are presented as geometric current densities J_geo_ by normalizing the currents to the electrode area (0.07 cm^2^). Across the investigated potential window, the Pd@Pd_0.2_Pt_0.2_Ir_0.2_Ru_0.2_Rh_0.2_ nanooctahedra exhibit the largest J_geo_, indicating the most effective HOR kinetics under identical testing conditions. In H_2_-saturated 0.1 M KOH at 1600 rpm, all electrodes display mass transport limited currents of 2.35 to 2.60 mA cm^–2^, in good agreement with literature values for alkaline HOR under comparable RDE conditions (typically 2.2–2.7 mA cm^–2^).^62^

**Figure 4:**
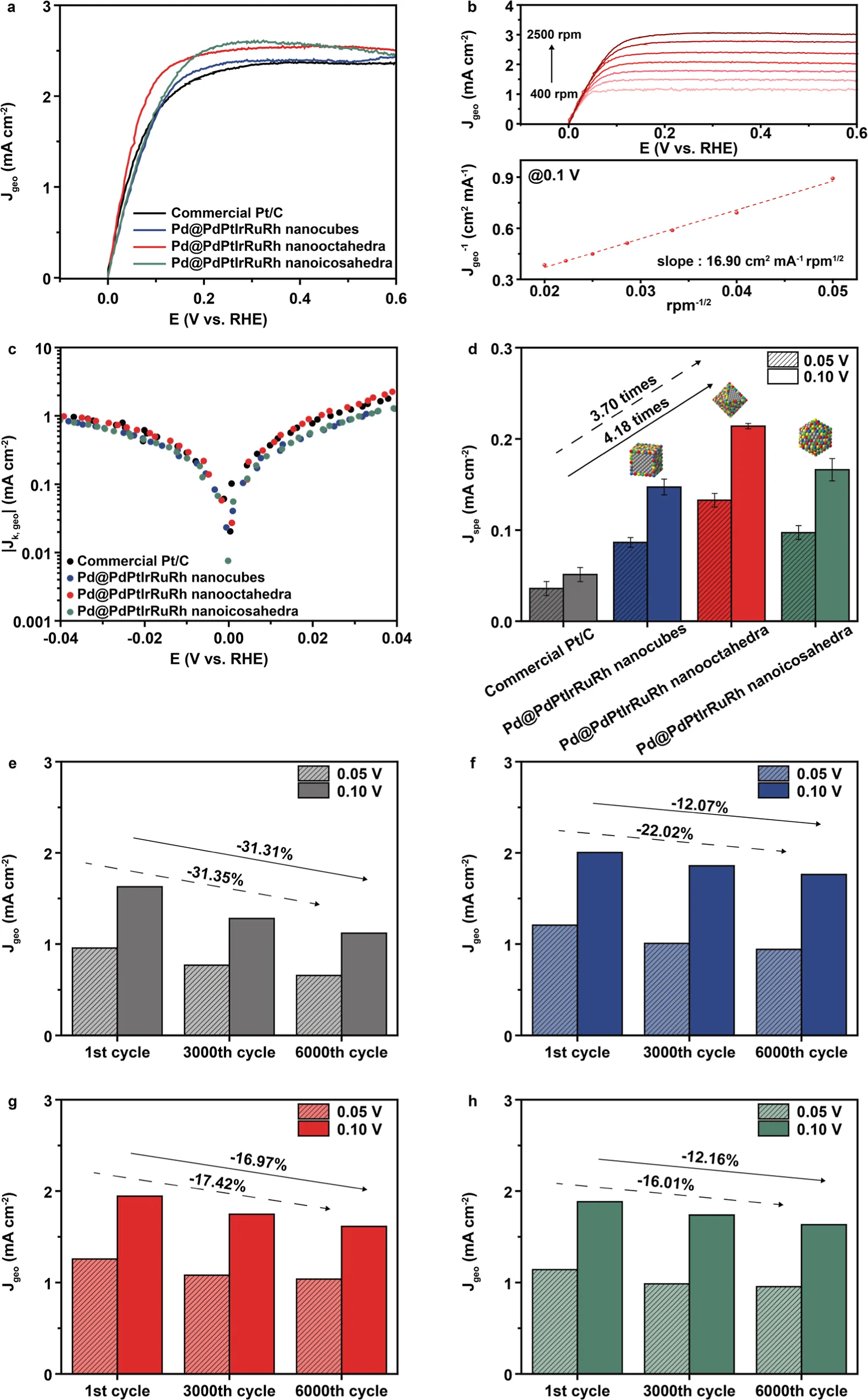
Alkaline HOR electrocatalytic performance of Pd@Pd_0.2_Pt_0.2_Ir_0.2_Ru_0.2_Rh_0.2_ core–shell nanocrystals and commercial Pt/C. (a) Geometric-area-normalized HOR LSV curves of Pd@Pd_0.2_Pt_0.2_Ir_0.2_Ru_0.2_Rh_0.2_ core–shell nanocubes, nanooctahedra, nanoicosahedra, and commercial Pt/C in H_2_-saturated 0.1 M KOH. (b) HOR LSV curves of Pd@Pd_0.2_Pt_0.2_Ir_0.2_Ru_0.2_Rh_0.2_ core–shell nanooctahedra at rotation rates of 400–2500 rpm, with the corresponding Koutecky-Levich plot at 0.10 V_RHE_. (c) Tafel plots derived from kinetic current densities for the Pd@Pd_0.2_Pt_0.2_Ir_0.2_Ru_0.2_Rh_0.2_ core–shell nanocrystals and commercial Pt/C. (d) ECSA-normalized HOR specific activities at 0.05 and 0.10 V_RHE_. (e–h) HOR durability comparison after 3000 and 6000 cycles for (e) commercial Pt/C, (f) Pd@Pd_0.2_Pt_0.2_Ir_0.2_Ru_0.2_Rh_0.2_ core–shell nanocubes, (g) core–shell nanooctahedra, and (h) core–shell nanoicosahedra.

To further interrogate HOR kinetics and the contribution of H_2_ transport, we examined the Pd@Pd_0.2_Pt_0.2_Ir_0.2_Ru_0.2_Rh_0.2_ nanooctahedra at rotation rates of 400, 625, 900, 1225, 1600, 2025, and 2500 rpm (Figure 4b). The diffusion-limited current rises with increasing rotation rate, reflecting faster replenishment of dissolved H_2_ and improved mass transport to the electrode surface. The inset of Figure 4b presents the corresponding Koutecky–Levich analysis at 0.10 V_RHE_, where 1/J_geo_ varies linearly with rpm^–1/2^. The extracted slope for the nanooctahedra (16.90 cm^2^ mA^–1^ rpm^1/2^) is close to the theoretical expectation,^63^ supporting the two-electron HOR pathway and indicating that the observed limiting currents are governed primarily by H_2_ mass transport. In addition, the kinetic current density (J_k_) was obtained using the Koutecky–Levich analysis, as shown in Figure 4c. Within the low overpotential window of 0–40 mV_RHE_, the Pd@Pd_0.2_Pt_0.2_Ir_0.2_Ru_0.2_Rh_0.2_ nanooctahedra consistently deliver the largest J_k_, indicating the most favorable HOR kinetics among the evaluated catalysts. The exchange current density (j_0_) was further assessed by linear fitting of the micropolarization region (−10 to +10 mV) based on the approximate Butler-Volmer equation (Figure S10). Notably, the Pd@Pd_0.2_Pt_0.2_Ir_0.2_Ru_0.2_Rh_0.2_ nanooctahedra exhibit the highest j_0_, further corroborating their superior intrinsic HOR activity. Moreover, the Tafel slopes of Pd@Pd_0.2_Pt_0.2_Ir_0.2_Ru_0.2_Rh_0.2_ nanocrystals with different morphologies, together with commercial Pt/C were extracted as shown in Figure S11. The Tafel slope values for Pd@Pd_0.2_Pt_0.2_Ir_0.2_Ru_0.2_Rh_0.2_ nanocubes, Pd@Pd_0.2_Pt_0.2_Ir_0.2_Ru_0.2_Rh_0.2_ nanooctahedra, Pd@Pd_0.2_Pt_0.2_Ir_0.2_Ru_0.2_Rh_0.2_ nanoicosahedra and commercial Pt/C are 126.07, 93.70, 119.07, and 119.56 mV dec^–1^, respectively. While all catalysts show Tafel slopes on the order of ∼90–130 mV dec^–1^, broadly consistent with a Volmer-influenced HOR pathway in alkaline media,^35,37,38^ the smaller slope for the {111}-enclosed nanooctahedra suggests a reduced kinetic penalty associated with the Volmer step. To further assess the reliability of the HOR kinetic analysis, post-measurement iR compensation and mass-transport correction were applied to the representative {111}-enclosed Pd@Pd_0.2_Pt_0.2_Ir_0.2_Ru_0.2_Rh_0.2_ core–shell nanooctahedra (Figure S12). Only minor differences were observed after correction, indicating that uncompensated resistance and residual mass-transport effects do not significantly affect the kinetic interpretation or overall activity trends.^64,65^

To decouple the contribution of surface area, the current densities were normalized to the electrochemical active surface area (ECSA), as determined from double-layer capacitance (C_dl_) measurements (Figures S13, S14). The ECSA-normalized specific activity (J_spe_) at 0.05 and 0.10 V_RHE_ was extracted from multiple HOR LSV measurements (Figure S15), and the averaged values with standard deviations from at least three independent measurements for each sample are summarized in Figure 4d. At both potentials, the J_spe_ values follow the same trend: Pd@Pd_0.2_Pt_0.2_Ir_0.2_Ru_0.2_Rh_0.2_ nanooctahedra > nanoicosahedra > nanocubes > Pt/C. In particular, the Pd@Pd_0.2_Pt_0.2_Ir_0.2_Ru_0.2_Rh_0.2_ nanooctahedra deliver an approximately 3.70-fold enhancement over Pt/C at 0.05 V_RHE_ and a 4.18-fold enhancement at 0.10 V_RHE_. Taken together, these comparisons indicate that the {111} facets featuring a hexagonal surface atomic arrangement provide the most favorable HEA surface motif for boosting alkaline HOR, outperforming twinned {111} surfaces where the hexagonal arrangement is interrupted by twin boundaries, as well as {100} surfaces with a square atomic arrangement.

Furthermore, the HOR durability of commercial Pt/C and the Pd@Pd_0.2_Pt_0.2_Ir_0.2_Ru_0.2_Rh_0.2_ nanocubes, nanooctahedra, and nanoicosahedra was assessed (Figures 4e–h and S16). Accelerated durability tests were conducted by continuous potential cycling between −0.10 and 0.70 V_RHE_ in H_2_-saturated 0.1 M KOH. After 6000 cycles, the nanooctahedra and nanoicosahedra retain most of their activity, showing less than 20% loss in current density measured at 0.05 and 0.10 V_RHE_, whereas commercial Pt/C exhibits a >30% decline under identical conditions. In addition, a slight decrease in the diffusion-limited current at higher potentials (for example, 0.50 V_RHE_) is observed after cycling, which is commonly attributed to the accumulation of surface oxygenated species such as adsorbed hydroxyl in alkaline media, as widely documented for HOR catalysts.^37,39,66,67^ Post-cycling TEM images further confirm that the Pd@Pd_0.2_Pt_0.2_Ir_0.2_Ru_0.2_Rh_0.2_ core–shell nanocrystals remain structurally intact after 6000 cycles, with no discernible cracking, fragmentation, or delamination from the Pd core (Figure S17). Additional post-catalysis HAADF-STEM and FFT analyses of the representative nanooctahedra confirm preservation of the nanooctahedral morphology and single-crystalline FCC structure, while EDS mapping shows overlapping distributions of Pd, Pt, Ir, Ru, and Rh without obvious elemental segregation (Figure S18). Collectively, these results demonstrate that Pd@Pd_0.2_Pt_0.2_Ir_0.2_Ru_0.2_Rh_0.2_ core–shell nanocrystals combine high activity with good durability, outperforming commercial Pt/C for alkaline HOR.

### Mechanistic Insight into HEA Surface Atomic Arrangements in Alkaline HOR

To elucidate how surface atomic arrangements on HEAs regulate alkaline HOR, we employed hydrogen underpotential deposition (HUPD) and CO stripping cyclic voltammetry (CV) as complementary probes of the surface affinity toward H* and OH*, respectively,^37,68,69^ with commercial Pt/C as a benchmark (Figure S19). In the HUPD profiles (Figure S19a), a more positive H* desorption peak indicates stronger hydrogen binding.^70^ The peak potentials follow the order Pt/C (0.22 V_RHE_) > Pd@Pd_0.2_Pt_0.2_Ir_0.2_Ru_0.2_Rh_0.2_ nanocubes (0.17 V_RHE_) > Pd@Pd_0.2_Pt_0.2_Ir_0.2_Ru_0.2_Rh_0.2_ nanooctahedra (0.16 V_RHE_) > Pd@Pd_0.2_Pt_0.2_Ir_0.2_Ru_0.2_Rh_0.2_ nanoicosahedra (0.15 V_RHE_), indicating that Pt/C binds H* most strongly, whereas the HEA shells exhibit weakened H* affinity. In addition, the slightly higher HUPD peak of the nanocubes relative to the two {111}-dominated surfaces indicates slightly stronger H* binding on the {100} surface. We then assessed OH* affinity using CO stripping (Figure S19b), where a lower CO oxidation peak potential reflects easier formation of oxygenated species that facilitate CO removal.^69^ The peak potentials increase in the sequence Pd@Pd_0.2_Pt_0.2_Ir_0.2_Ru_0.2_Rh_0.2_ nanocubes (0.58 V_RHE_) < nanooctahedra (0.61 V_RHE_) < nanoicosahedra (0.63 V_RHE_) < Pt/C (0.67 V_RHE_). Pt/C also shows an additional higher potential feature near 0.77 V_RHE_. These results indicate that the HEA-coated Pd nanocrystals facilitate OH* formation more readily than Pt/C, and that OH* adsorption on the {111} octahedral surface is intermediate between that on {100} and on the twin-interrupted {111} surface. Overall, the combined HUPD and CO stripping trends suggest that {111} facets with an uninterrupted hexagonal surface arrangement offer a more moderate H* and OH* binding motif, which has been widely associated with optimized alkaline HOR activity.^41,71^

To probe potential dependent electronic and coordination changes during alkaline HOR, we performed *in situ* synchrotron XAS on Pd@Pd_0.2_Pt_0.2_Ir_0.2_Ru_0.2_Rh_0.2_ core–shell nanooctahedra in 0.1 M KOH. Pd–K edge, Pt-L_3_ edge, Ir-L_3_ edge, Ru–K edge, and Rh–K edge spectra were collected in the dry state (in air) and after electrolyte immersion under applied potentials of −0.05, +0.05, and +0.15 V_RHE_ (Figures 5 and S20). This potential window is relevant to H_2_ fed AEMFC anode conditions, where the HOR electrode is often expected to lie near 0 V_RHE_, while extending to slightly positive potentials to enhance sensitivity to bias induced changes in surface electronic structure and local coordination. With increasing potential, the Pt-L_3_ edge shows a progressive increase in white line intensity (Figure 5a), consistent with an electron-depletion tendency at Pt-related surface sites, whereas the Ir-L_3_ edge exhibits the opposite behavior with a decreased white line intensity (Figure 5c), suggesting an electron-enrichment tendency at Ir-related motifs. In parallel, the Ru–K edge shifts toward higher photon energy under applied bias (Figure 5e), indicating an electron depletion at Ru-related motifs during the reaction. These changes point to a potential-dependent redistribution of electron density across the HEA surface, which may be linked to differentiated roles of Pt-, Ir-, and Ru-centered sites in governing adsorbate binding and interfacial reaction steps during HOR.

**Figure 5:**
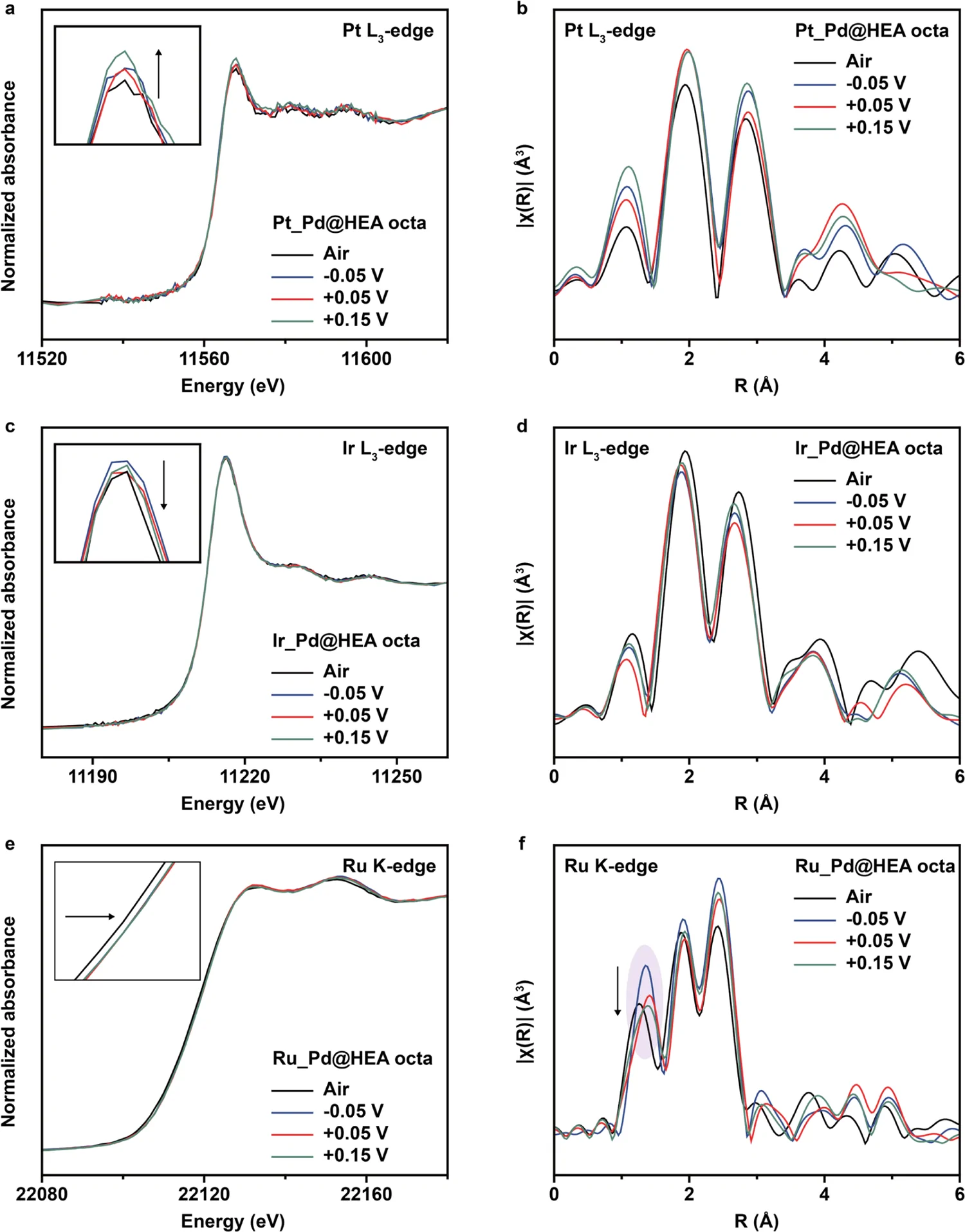
*In situ* synchrotron XAS analysis of Pd@Pd_0.2_Pt_0.2_Ir_0.2_Ru_0.2_Rh_0.2_ core–shell nanooctahedra during alkaline HOR. XANES and FT-EXAFS spectra were collected in air and at applied potential of −0.05, 0.05, and 0.15 V_RHE_ at (a, b) Pt L_3_-edge, (c, d) Ir L_3_-edge, and (e, f) Ru K-edge.

In parallel, the corresponding FT-EXAFS spectra of Pt and Ir show only subtle potential-dependent variations, with no pronounced changes in the main metal–metal coordination features, indicating that their local coordination frameworks remain largely preserved under the applied potentials (Figure 5b, d). Importantly, consistent with the potential-driven electronic redistribution, the Ru FT-EXAFS profiles exhibit a clear evolution of a short-range feature at ∼1.5 Å as the bias is stepped (Figure 5f), which we attribute to potential-dependent interactions between Ru-containing motifs and oxygenated adsorbates.^72^ Relative to the spectrum collected in air, electrolyte immersion and polarization at −0.05 V_RHE_ lead to a pronounced enhancement together with a slight shift of this short-range feature, indicating a substantial change in the local Ru coordination environment upon contact with the alkaline electrolyte. This feature is consistent with additional Ru–O coordination involving adsorbed OH* and related oxygenated species at Ru-based surface sites.^73^ Upon increasing the potential to +0.05 and +0.15 V_RHE_, the feature progressively decreases and its profile evolves, demonstrating that the local Ru–O coordination environment is sensitive to the applied potential. Because the FT-EXAFS amplitude and apparent radial position can be influenced by coordination number, Ru–O bond-distance distribution, and local structural disorder, these changes cannot be directly interpreted as quantitative variations in OH* surface coverage.^74,75^ Instead, they indicate a potential-dependent reorganization of Ru-bound oxygenated species and suggest that, relative to the other four constituent metals, Ru-containing motifs exhibit the most pronounced interaction with oxygenated intermediates during alkaline HOR.^76,77^

To further distinguish reversible adsorbate-induced changes from irreversible structural relaxation, an additional Ru K-edge potential-cycling experiment was performed at open circuit potential (OCP), +0.60 V_RHE_, and back to OCP (Figure S21). The +0.60 V_RHE_ condition was intentionally selected as a relatively high anodic potential to provide a more stringent test of the structural reversibility of Ru-containing sites. The Ru K-edge XANES spectra show only minor changes throughout the cycle and largely recover after returning to OCP, indicating no pronounced irreversible change in the average Ru electronic structure. In the corresponding FT-EXAFS spectra, the short-range ∼1.5 Å feature decreases at +0.60 V_RHE_ and largely recovers after returning to OCP, whereas the higher-shell Ru-M coordination features remain essentially unchanged. These results indicate that the potential-dependent Ru spectral evolution predominantly reflects reversible changes in the local Ru coordination environment rather than irreversible rearrangement of the HEA framework.

It is noteworthy that the Pd- and Rh–K edge positions show no appreciable change over the investigated potential range during alkaline HOR (Figure S20), indicating that the average electronic states of Pd and Rh motifs remain largely stable under these conditions. However, this does not exclude their catalytic involvement, as their contributions may arise from multicomponent electronic coupling and ensemble effects that are not directly reflected by edge-position shifts. To further clarify the catalytic contribution of Pd and Rh, we compared the alkaline HOR activities of Pd@Pd_0.2_Pt_0.2_Ir_0.2_Ru_0.2_Rh_0.2_ nanooctahedra with those of compositionally simplified Pd@Pt_0.33_Ir_0.33_Ru_0.33_ nanooctahedra, along with Pd@Pt_0.5_Ru_0.5_, Pd@Pt, and Pd@Ru nanooctahedra as mono- and bimetallic control samples (Figure S22). Among these catalysts, Pd@Pd_0.2_Pt_0.2_Ir_0.2_Ru_0.2_Rh_0.2_ nanooctahedra exhibit the highest HOR activity. In particular, their ECSA-normalized specific activity is higher than that of Pd@Pt_0.33_Ir_0.33_Ru_0.33_ nanooctahedra at both 0.05 and 0.10 V_RHE_. This comparison indicates that, although Pd and Rh do not show pronounced potential-dependent edge shifts in the *in situ* XAS spectra, their incorporation still contributes positively to the catalytic performance, likely through multicomponent electronic coupling and ensemble effects within the HEA surface.

For the *in situ* synchrotron AP-XPS measurements, a custom anion-exchange-membrane (AEM)-based liquid cell was employed, in which H_2_-saturated 0.1 M KOH was continuously circulated to supply dissolved H_2_ to the catalyst/AEM interface (Figure S23).^78^ The hydrated AEM maintained ionic and water transport pathways, while a positive current was consistently observed under applied potentials, confirming electrochemical oxidation under the measurement conditions. To monitor how the surface chemical states evolve with applied bias during alkaline HOR, we conducted *in situ* AP-XPS measurements on Pd@Pd_0.2_Pt_0.2_Ir_0.2_Ru_0.2_Rh_0.2_ core–shell nanooctahedra in a thin-layer 0.1 M KOH electrolyte, under 0.3 mbar H_2_O vapor (Figure 6). Spectra were collected sequentially at OCP, +0.05 V_RHE_, +0.60 V_RHE_, and after returning to OCP. The +0.60 V_RHE_ step was intentionally included as an accelerated anodic condition to enhance the sensitivity of XPS to bias induced changes in surface composition and oxidation related chemistry within the measurement time scale. The return to OCP enables evaluation of the reversibility of these surface changes and any hysteresis after exposure to elevated bias. We monitored the Pd 3d, Pt 4f, Ir 4f, Ru 3d, and Rh 3d regions, and quantified the relative fractions of metallic and oxidized species using consistent peak deconvolution and area analysis (Figure 6a–e and Table S4). The O 1s signal was collected in parallel to track the evolution of oxygen-containing species (Figure 6f and Table S4). The O 1s spectra are dominated by two water-related contributions at higher binding energies, assigned to gas-phase and liquid-phase H_2_O. At lower binding energies, the M-O contribution increases slightly with increasing potential, whereas the OH-related contribution shows a modest decrease, consistent with a subtle increase in metal–oxygen coordination under anodic polarization.

**Figure 6:**
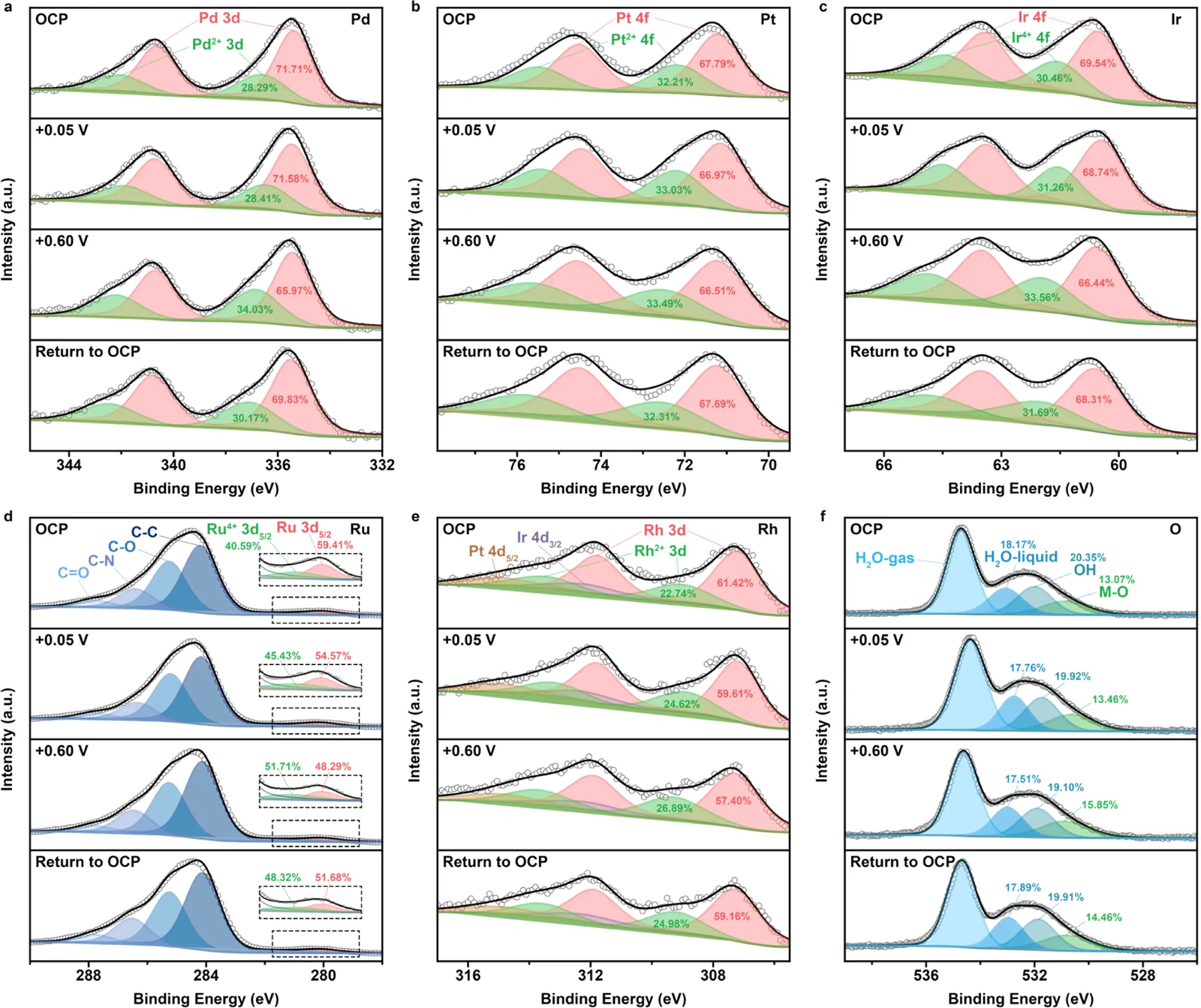
*In situ* synchrotron AP-XPS analysis of Pd@Pd_0.2_Pt_0.2_Ir_0.2_Ru_0.2_Rh_0.2_ core–shell nanooctahedra under alkaline HOR condition. (a) Pd 3d, (b) Pt 4f, (c) Ir 4f, (d) Ru 3d, (e) Rh 3d, and (f) O 1s spectra of Pd@Pd_0.2_Pt_0.2_Ir_0.2_Ru_0.2_Rh_0.2_ core–shell nanooctahedra measured at applied potential of OCP, +0.05, +0.60 V_RHE_, and returning to OCP.

For Pd, Pt, Ir, and Rh, the oxidized fractions increase only slightly upon stepping to anodic potentials, whereas the metallic components again predominate after returning to the OCP state, indicating largely reversible surface redox behavior with only very limited anodic oxidation even at +0.60 V_RHE_. For example, the Pt^2+^ fraction changes only slightly from 32.21% at OCP to 33.03% at +0.05 V_RHE_ and 33.49% at +0.60 V_RHE_, and then decreases to 32.31% after returning to OCP. Likewise, the Ir^4+^ fraction increases from 30.46% at open circuit potential to 31.26% at +0.05 V_RHE_ and 33.56% at +0.60 V_RHE_, before decreasing to 31.69% upon returning to OCP. In contrast, the Ru 3d region exhibits a stronger potential dependence (Figure 6d). Quantitative deconvolution shows that the oxidized Ru component (assigned as Ru^4+^) increases as the potential is raised, with the Ru^0^ fraction decreasing from 59.41% at OCP to 54.57% at +0.05 V_RHE_ and further to 48.29% at +0.60 V_RHE_, while the Ru^4+^ fraction increases correspondingly from 40.59% to 45.43% and 51.71%, respectively. Notably, after returning to OCP, the Ru^0^ fraction largely recovers to 51.68% (Ru^4+^ 48.32%), although a modest residual change remains. This recovery indicates that the Ru surface chemical-state evolution is predominantly reversible, while the remaining deviation is somewhat more pronounced than those observed for Pd, Pt, Ir, and Rh under the same *operando* sequence. This behavior can be rationalized by two complementary considerations. First, Ru is more susceptible to anodic oxidation in alkaline media, and oxidized Ru species formed at the relatively high anodic potential of +0.60 V_RHE_ may not be fully reduced upon returning to OCP within the experimental time window, consistent with previous literature.^77,79^ Second, the increased oxidized Ru contribution may also include Ru sites coordinated to strongly bound oxygenated species, including OH*, which can contribute to an apparent oxidized signature in XPS. Importantly, this AP-XPS trend should not be interpreted as a direct quantitative measure of OH* surface coverage. The Ru 3d AP-XPS spectra probe changes in the surface chemical state of Ru, whereas the ∼1.5 Å Ru K-edge FT-EXAFS feature reflects the ensemble-averaged local Ru coordination environment and is additionally sensitive to coordination number, bond-distance distribution, and local structural disorder. Therefore, the increase in the oxidized Ru contribution observed by AP-XPS and the decrease in the ∼1.5 Å FT-EXAFS feature at higher potential are not necessarily contradictory. Rather, the combined *in situ* AP-XPS and XAS results demonstrate that Ru-containing motifs exhibit the most pronounced potential-dependent interaction with oxygenated species among the constituent metals during alkaline HOR.

Furthermore, building on these experimental insights, we next perform DFT calculations to evaluate the adsorption free energies of H* (ΔG_H*_) and OH* (ΔG_OH*_), as well as the reaction energy barriers, on the {111} facets with a hexagonal atomic arrangement of Pd@Pd_0.2_Pt_0.2_Ir_0.2_Ru_0.2_Rh_0.2_ core–shell nanooctahedra. For comparison, analogous calculations are also carried out on the {100} facets with a square atomic arrangement of the corresponding core–shell nanocubes to assess facet-dependent trends. To account for the configurational diversity of the HEA surfaces, 12 independently randomized {100} slab models and 19 independently randomized {111} slab models were considered, covering a broad range of local elemental configurations (Figures S24 and S25) relevant to the adsorption properties and catalytic behavior of multimetallic active sites.^80^ In general, high catalytic performance arises when the binding of reaction intermediates is appropriately moderated, consistent with the Sabatier principle that catalyst–intermediate interactions should avoid both overbinding and underbinding.^71^ As illustrated in Figure 7a, the atomic scale models highlight the distinct surface symmetries and atomic packings of the representative {100} and {111} facets. The {100} surface exhibits a square atomic arrangement with 4-fold symmetry, generating a periodic lattice of adsorption environments that includes on-top sites, bridge sites, and hollow sites. In contrast, the {111} surface adopts a hexagonally packed arrangement with 3-fold symmetry, generating on-top, bridge, and hollow sites that differ from those on the square {100} surface. The adsorption-site classifications and corresponding frequencies reported in Figures S26 and S27 were determined from the fully relaxed H* and OH* adsorption structures rather than from their initial adsorption positions. These symmetry-defined motifs provide diverse H* and OH* binding configurations and multi-atom ensembles, thereby shaping the facet-dependent adsorption landscape and catalytic behavior.

**Figure 7:**
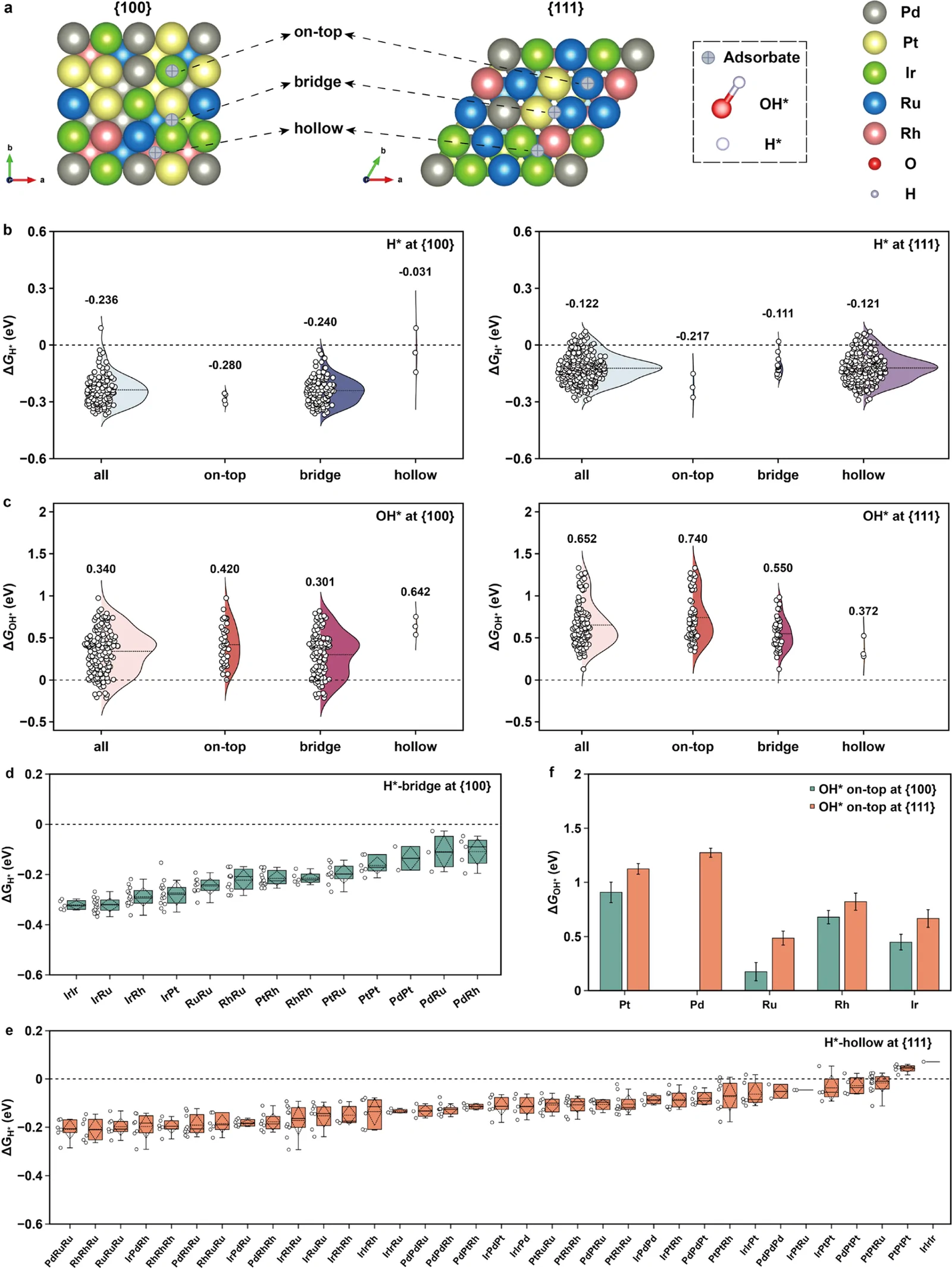
Hydrogen and hydroxyl adsorption behaviors on Pd_0.2_Pt_0.2_Ir_0.2_Ru_0.2_Rh_0.2_ HEA surfaces investigated via DFT calculations. (a) Top views of the atomic configurations for the {100} and {111} facets, illustrating the typical adsorption sites (on-top, bridge, and hollow). (b, c) Overall distributions of the calculated Gibbs free energies for (b) hydrogen (ΔG_H*_) and (c) hydroxyl (ΔG_OH*_) adsorption at different sites on the {100} and {111} facets. (d, e) Component-specific analysis of ΔG_H*_ showing the dependence of adsorption energies on the local atomic environments at (d) various bridge sites on the {100} facet and (e) various hollow sites on the {111} facet. (f) Comparison of ΔG_OH*_ at the on-top sites of individual metallic elements on both {100} and {111} facets. Note that stable OH* adsorption was not observed on the Pd on-top site at the {100} facet, suggesting its intrinsically weak binding affinity for hydroxyl species.

Considering the chemically and geometrically diverse coordination environments on Pd_0.2_Pt_0.2_Ir_0.2_Ru_0.2_Rh_0.2_ surfaces, we first evaluate ΔG_H*_ by sampling an ensemble of more than 100 distinct hydrogen adsorbed configurations on both facets. As shown in Figures 7b and S26, the {100} surface exhibits a more negative overall average ΔG_H*_ than {111} (mean ΔG_H*_ = −0.236 eV for {100} versus −0.122 eV for {111}), indicating that the {111} facet is closer to the thermoneutral ideal (ΔG_H*_ ≈ 0 eV). This facet-dependent contrast is further supported by the site-resolved statistics. On {100}, hydrogen adsorption occurs predominantly at bridge sites, characterized by a representative mean ΔG_H*_ of −0.240 eV. In contrast, on {111}, adsorption is dominated by hollow sites with a less negative ΔG_H*_ (−0.121 eV), consistent with an overall weaker hydrogen-binding landscape. We then evaluate ΔG_OH*_ by sampling a broad set of OH* configurations on both surfaces. Figures 7c and S27 show that the {100} facet yields systematically lower ΔG_OH*_ values than {111} (average ΔG_OH*_ = 0.340 eV for {100} versus 0.652 eV for {111}), implying a stronger affinity for OH* on {100}. The preferred adsorption motif on {100} is the bridge site, with a representative ΔG_OH*_ of 0.301 eV, while on-top site is the next most common but less favorable (0.420 eV). In contrast, OH* on {111} most frequently occupies on-top sites, which are associated with a higher ΔG_OH*_ (0.740 eV), and the second most populated bridge sites remain comparatively strongly bound (0.550 eV). Notably, the broad distributions for each site type highlight the intrinsic heterogeneity of multimetallic surface ensembles, where local elemental arrangements modulate adsorption strength even within the same nominal adsorption motif. Overall, the two facets exhibit distinct adsorption preferences: the {100} surface binds both H* and OH* more strongly, whereas the {111} surface provides a comparatively moderated adsorption landscape. These facet-dependent trends are consistent with the electrochemical HUPD and CO-stripping results (Figure S19).

Additionally, to pinpoint the atomic ensembles that are most likely to function as hydrogen adsorption and catalytically active sites at the preferred bridge positions on the {100} facets and the 3-fold hollow sites on the {111} facets, we conducted site resolved ΔG_H*_ analyses across representative elemental configurations (Figure 7d and e). On {100} (Figure 7d), Ir-based bridge ensembles, defined as bridge sites where Ir pairs with one of the constituent elements including itself (Ir–Ir, Ir–Ru, Ir–Rh, and Ir–Pt), consistently deliver more negative ΔG_H*_ values (approximately −0.22 to −0.37 eV), indicating stronger H binding. By contrast, Pd-based bridge ensembles on {100} exhibit the least negative ΔG_H*_ values among all evaluated sites, suggesting the weakest H adsorption. Turning to the hollow sites on the {111} facets (Figure 7e), the site preference shifts noticeably. Interestingly, the relatively stronger adsorption motifs are mainly associated with Ru- and Rh-based hollow ensembles, indicating moderately enhanced H binding. In contrast, Pt-based hollow ensembles exhibit the weakest adsorption on {111}, with ΔG_H*_ values much closer to thermoneutral adsorption. This trend is further quantified in Figure S28, which summarizes the average ΔG_H*_ values of hollow ensembles on the {111} facets. Each ensemble comprises three atoms forming one hollow site, where the main-metal (M) denotes the two identical atoms (MM) and the sub-metal (N) denotes the third atom (MMN). Among these motifs, RuRuN and RhRhN ensembles consistently exhibit more negative ΔG_H*_ values, indicating stronger H binding on {111}. Specifically, RuRuN spans from −0.11 eV (RuRuPt) to −0.21 eV (RuRuPd) and remains strongly binding when N is Rh or Ru (RuRuRh = −0.19 eV and RuRuRu = −0.20 eV). A similar trend is observed for RhRhN, which ranges from −0.11 eV (RhRhPt) to −0.21 eV (RhRhRu), with the strongest adsorption when N is Ru or Rh. In contrast, PtPtN motifs remain close to thermoneutral adsorption across nearly all combinations, with average ΔG_H*_ values of approximately +0.04 to −0.07 eV, indicating intrinsically weak H binding on {111} largely independent of N. Moreover, as sub-metal (N) is Pt, we found that the H binding becomes weaker than the others in the same main-metal based group. However, interestingly Ir-based ensembles (IrIrN) show relatively moderate ΔG_H*_ values of approximately +0.07 to −0.14 eV compared to RuRuN or RhRhN motifs, suggesting that Ir-based sites might serve as better candidate sites for H_2_ activation, generating adsorbed H* that subsequently diffuses to neighboring Pt-based motifs, where the more thermoneutral H* adsorption facilitates the subsequent HOR reaction.

To further resolve how the surrounding multimetallic environment contributes to these adsorption trends, we performed an element-resolved radial distribution function (RDF) analysis around H* on the {111} surface (Figure S29). Because H* predominantly occupies hollow sites, the first-nearest-neighbor (1NN) coordination shell mainly reflects the three metal atoms constituting the local hollow adsorption ensemble. The most pronounced adsorption-energy-dependent variations occur within this 1NN region, indicating that the identities of the directly coordinated metal atoms play a dominant role in regulating H* binding. In particular, increased contributions from Ru, Rh, and Pd are generally associated with stronger H* adsorption, whereas a higher Pt population shifts H binding toward weaker and more thermoneutral values, consistent with the site-resolved trends in Figures 7e and S28. Ir exhibits a comparatively weaker dependence on H* adsorption strength and remains represented across a broad range of adsorption energies, suggesting that its contribution is more strongly influenced by the surrounding multimetallic environment. This behavior is also consistent with the presence of Ir-containing hollow ensembles, including IrIrM configurations, whose H* adsorption energies can remain relatively close to the thermoneutral region. Overall, these results show that H* adsorption on the HEA {111} surface is governed by cooperative mixed-metal hollow ensembles rather than by isolated single-metal adsorption centers.

We next compare OH* adsorption by analyzing ΔG_OH*_ on the {100} and {111} surfaces (Figure 7f). To provide a simple site-specific comparison of the elemental effect, we first focus on on-top sites. Across evaluated on-top motifs, Ru sites consistently exhibit the strongest OH* affinity, evidenced by the lowest ΔG_OH*_ values. For instance, on the most populated on-top sites on {111}, Ru shows a ΔG_OH*_ of around +0.48 eV, suggesting that OH* preferentially adsorbs on Ru. For on-top sites, the OH* binding strength follows the order Ru > Ir > Rh > Pt > Pd, and a similar trend is also observed for on-top sites on {100}, confirming Ru as the primary OH* binding center across facets. Notably, this preference is consistent with our *in situ* XAS results (Figure 5f), which reveal the emergence of Ru–O scattering contributions attributable to adsorbed OH* (and related oxygenated species) at Ru-based surface sites. To assess the local environments associated with OH* adsorption across all relaxed configurations, including both on-top and bridge sites, we further performed an element-resolved RDF analysis on the {111} surface (Figure S30). Unlike H*, which predominantly occupies hollow sites, OH* mainly adsorbs at on-top and bridge sites, consistent with the site-distribution analysis in Figure 7c. The largest adsorption-energy-dependent variations occur within the 1NN shell, where Ru and Rh are more prominent in the stronger OH-binding regime, indicating their favorable contributions to OH* binding. In contrast, Pt and Pd are preferentially associated with weaker OH* adsorption and contribute little direct coordination within the strongest-binding configurations, whereas Ir exhibits an intermediate behavior. Notably, Pd remains present in the more distant coordination shells even for strongly bound OH* configurations, suggesting that it may also influence OH* adsorption indirectly through the surrounding multimetallic environment rather than acting as a primary OH-binding center. Taken together with the H* RDF analysis, these results indicate that adsorption on the HEA surface is governed not only by the identities of the directly coordinated metal atoms but also by the broader multimetallic environment, providing a microscopic basis for the cooperative regulation of H* and OH* during alkaline HOR.

To further elucidate the possible catalytic mechanism of Pd@Pd_0.2_Pt_0.2_Ir_0.2_Ru_0.2_Rh_0.2_ core–shell nanooctahedra, we performed CI-NEB calculations to map the alkaline HOR energy profiles. Experimentally, the nanooctahedra exhibit a Tafel slope of 93.70 mV dec^–1^ (Figure S11), which is consistent with a Volmer-influenced pathway involving H_2_O formation as a possible rate-determining step.^35,37,38^ Therefore, our theoretical analysis focused on the Volmer pathway. *In situ* XANES shows that Pt exhibits an electron-deficient tendency during anodic polarization (Figure 5a), whereas Ir tends to gain electron density (Figure 5c). Meanwhile, *in situ* XANES and XPS suggest that Ru remains partially oxidized in alkaline electrolyte, and in situ EXAFS reveals the appearance of a Ru–O scattering contribution, indicating possible interactions between Ru and oxygenated species (Figures 5e f and 6d). These spectroscopic observations suggest a plausible cooperative reaction motif involving adjacent H* and OH* species, represented as Pt­(H*)–Ru­(OH*) (Figure 8a). In this proposed configuration, OH* may be anchored on the oxophilic Ru site, while H* is adsorbed on a neighboring Pt site with near-thermoneutral ΔG_H*_. As H* approaches OH* to form H_2_O, electron density may be drawn toward the electronegative oxygen atom, which could account for the electron-deficient Pt signature observed by *in situ* XAS (Figure 5). The electron-deficient character of Ru is likely associated with its strong OH* adsorption affinity in alkaline electrolyte. These assignments are also consistent with the DFT-derived adsorption energetics (Figure 7), which suggest favorable H* adsorption on Pt-containing sites and stronger OH* affinity at Ru-containing motifs. Together, these results suggest that Pt-based H* adsorption sites and Ru-based OH* adsorption sites may form cooperative multi-atom ensembles that facilitate the Volmer step during alkaline HOR.

**Figure 8:**
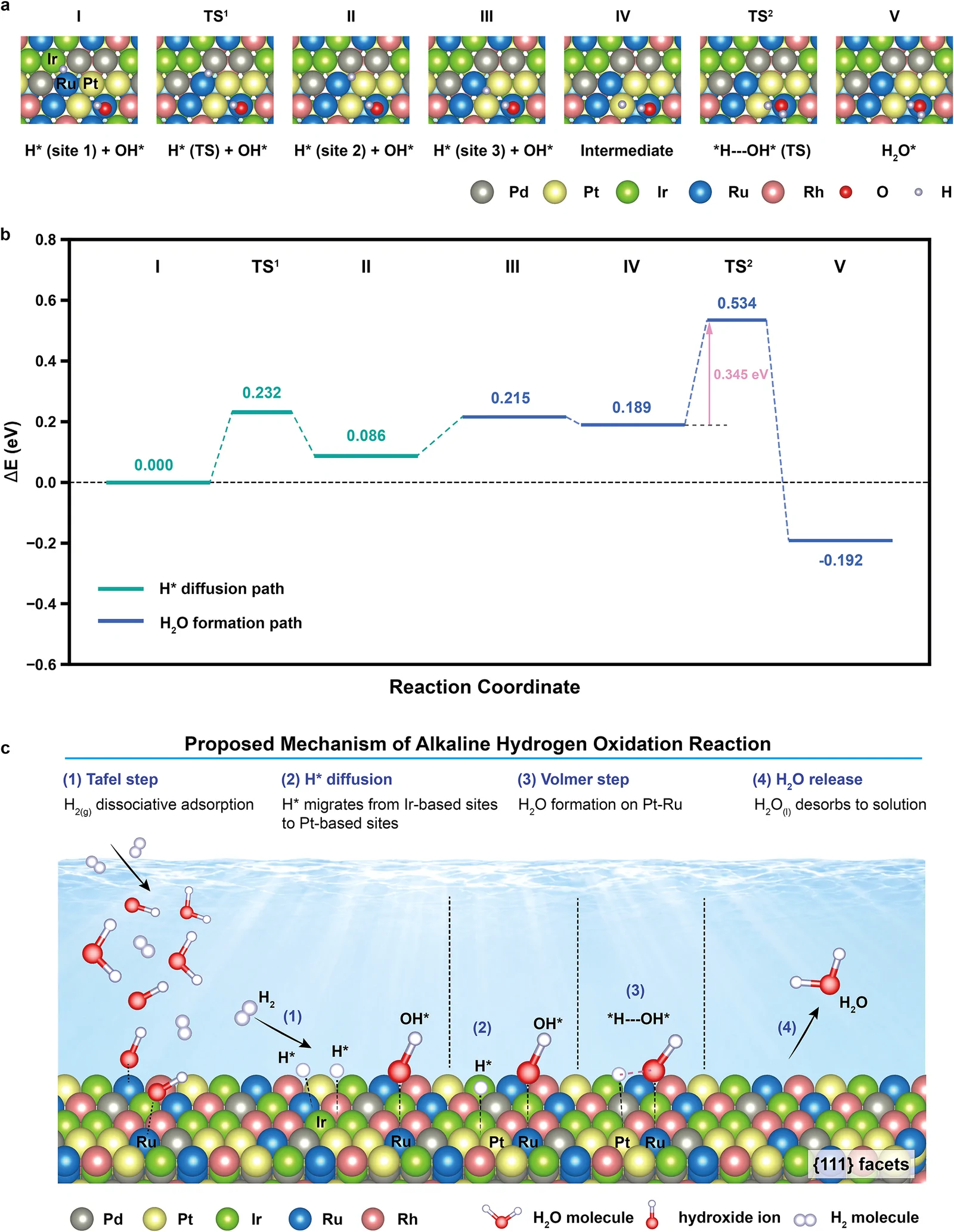
Mechanistic study of the alkaline HOR on {111} facet of Pd_0.2_Pt_0.2_Ir_0.2_Ru_0.2_Rh_0.2_ HEA. (a) Atomic configurations and (b) corresponding relative energy profile along the proposed reaction coordinate derived from theoretical calculations. The green and blue lines represent the H* diffusion pathway and H_2_O formation pathway, respectively, with the latter corresponding to the Volmer step. (c) Schematic illustration of the proposed alkaline HOR mechanism on the {111} facet of the HEA.

Based on these considerations, we modeled a possible Volmer reaction pathway for activation barrier calculations (Figure 8). In this pathway, an H* atom initially adsorbed at a Pt-containing hollow site, considered a plausible active site based on the adsorption-energy analysis, migrates to a neighboring Pt atop site and subsequently couples with OH* adsorbed on an adjacent Ru atom to form H_2_O (Figure 8a). The CI-NEB results support this proposed pathway (Figure 8b). Along the reaction coordinate, H* first migrates from the PtPtRu hollow site to a thermodynamically more favorable Pt atop site, forming the proposed Pt­(H*)-Ru­(OH*) intermediate. This intermediate then undergoes H* and OH* coupling to generate H_2_O, with an activation barrier of 0.345 eV at the transition state (TS). These results suggest that the Volmer process can proceed through a bifunctional pathway, in which the local composition and {111} surface arrangement of the HEA nanooctahedra cooperatively regulate H* and OH* adsorption, thereby facilitating alkaline HOR kinetics.^40^

In alkaline HOR, the facility of surface hydrogen diffusion may also influence the overall catalytic activity, particularly on HEA surfaces where H* adsorption strengths are distributed across diverse local atomic environments. Using the same slab model, we identified a continuous H* diffusion pathway across the {111} surface (Figure 8a, b). Along this pathway, H* migrates from a strongly binding IrRuPd hollow site, denoted as H* site 1, to a weaker binding PtPtRu hollow site, denoted as H* site 3 (Figure 7e). The initial diffusion step requires an activation barrier of 0.232 eV, while the transition from stage II, corresponding to H* hollow site 2, to stage III, corresponding to H* hollow site 3, involves an uphill energy change of 0.129 eV. These energetic requirements are lower than the subsequent Volmer coupling barrier of 0.345 eV, suggesting that H* diffusion is kinetically accessible and is unlikely to be the RDS under the examined conditions. This result is consistent with the experimentally observed Tafel slope, which supports a Volmer-influenced pathway on the HEA catalyst.

To further clarify whether H* spillover can proceed across the {111} surface and deliver H* to moderately binding active sites, such as Pt-containing hollow sites, we compared three representative hydrogen diffusion pathways (Figure S31). These pathways originate from structurally analogous hollow ensembles, including IrIrRh, PtPtRh, and RuRuRh, and follow similar hollow-to-hollow trajectories toward the same PtPtRu active site. The intrinsic H* adsorption strengths of the initial sites follow the order RuRuRh > IrIrRh > PtPtRh. To minimize energy variations caused by different local atomic environment, the coordination environments surrounding the primary diffusion pathways were kept consistent. The CI-NEB results show that Pathway 2, involving diffusion from a Pt-containing hollow site to the active site PtPtRu, has the lowest activation barrier of 0.132 eV, whereas diffusion from Ru- and Ir-containing hollow sites requires higher barriers of 0.234 and 0.285 eV, respectively (Figure S31). These results suggest that, in the examined pathways, Pt-containing ensembles not only provide favorable sites for H* adsorption and subsequent water formation but may also facilitate surface H* diffusion.^23,81,82^ Moreover, all three diffusion barriers remain lower than the Volmer coupling barrier of 0.345 eV, suggesting that H* spillover from stronger binding sites to moderately binding sites across the hexagonally arranged HEA surface is both thermodynamically and kinetically accessible. This comparison further supports that the Volmer step, rather than H* diffusion, is the likely RDS under the examined conditions. However, the relative rates of H* adsorption and diffusion, OH* adsorption, OH* accumulation, and H_2_O formation may vary with the applied potential and are not explicitly resolved by the present static DFT calculations.

Taken together, these results rationalize why the {111}-enclosed HEA nanooctahedra exhibit superior alkaline HOR activity, as schematically illustrated in Figure 8c. On the hexagonally arranged {111} surface, H_2_ may first undergo dissociative chemisorption on relatively strong H-binding ensembles, followed by H* diffusion toward Pt-containing sites with more moderate hydrogen binding. In parallel, OH^–^ ions from the alkaline electrolyte preferentially interact with oxophilic Ru-containing sites, forming neighboring OH* species. Once H* reaches a Pt site adjacent to an OH*-occupied Ru site, the two intermediates may cooperatively couple to form H_2_O, followed by product desorption into the electrolyte. Although other reaction pathways may also operate on the compositionally diverse HEA surface, this proposed pathway is consistent with the spectroscopic observations, DFT-derived adsorption energetics, and CI-NEB barrier analysis. This pathway differs from conventional mono- or bimetallic catalysts, which often rely on a single dominant active site or a simple two-component bifunctional interface. Instead, the HEA {111} surface provides a distributed network of local atomic ensembles with different H* and OH* binding strengths, enabling H_2_ activation, H* diffusion, OH* adsorption, and H_2_O formation to be spatially coordinated.^12,82^ Such symmetry-selected multi-atom ensembles create a continuous reaction network for alkaline HOR, demonstrating the unique advantage of controlling surface atomic arrangements in HEA electrocatalysts.

## Conclusion

In summary, this work establishes a direct connection between surface atomic arrangement and alkaline HOR activity on PdPtIrRuRh HEA nanocrystals. By growing compositionally comparable HEA shells on Pd seeds with cubic, octahedral, and icosahedral shapes, we constructed three well-defined surface configurations, including square {100}, hexagonal {111}, and twin-interrupted {111} facets. This design allows the role of surface symmetry to be distinguished from the overall compositional effect. Among these structures, the {111}-enclosed HEA nanooctahedra deliver the highest intrinsic HOR activity, with a 4.18-fold enhancement over commercial Pt/C at 0.10 V_RHE_, demonstrating that the hexagonal surface arrangement provides a more favorable catalytic environment than square or twin-interrupted surfaces. In addition, the HEA nanooctahedra exhibit good structural and electrochemical durability under alkaline operating conditions. Combined spectroscopic and computational analyses further reveal the origin of this facet-dependent activity. *In situ* XAS and AP-XPS suggest potential-dependent interactions between Ru-containing motifs and oxygenated species, together with electronic-structure coupling among the constituent metals during HOR. DFT and CI-NEB calculations show that the hexagonal {111} surface provides symmetry-selected multi-atom ensembles that balance H* and OH* adsorption, enable H* spillover, and facilitate H_2_O formation. The calculated energy profiles further support a Volmer-influenced pathway under the examined conditions. Unlike conventional mono- or bimetallic catalysts that often rely on a limited number of active-site motifs, the HEA {111} surface offers a distributed network of local atomic ensembles with varied adsorption strengths, enabling cooperative and spatially coordinated alkaline HOR kinetics. These findings identify surface atomic arrangement as a critical descriptor for HEA electrocatalysts and provide design principles for optimizing multicomponent catalytic surfaces beyond composition control alone.

## Experimental and Computational Details

### Synthesis of Pd@Pd_0.2_Pt_0.2_Ir_0.2_Ru_0.2_Rh_0.2_ Core–shell Nanocrystals with Controlled Facets

Pd@Pd_0.2_Pt_0.2_Ir_0.2_Ru_0.2_Rh_0.2_ core–shell nanocrystals with controlled facets were synthesized using a dropwise addition method based on a seed-mediated growth strategy. In a typical synthesis, 0.4 mL of Pd seed suspension in EG, using cubic, octahedral, and icosahedral Pd seeds, was mixed with PVP (100 mg), KBr (60 mg), AA (20 mg), and EG (3.1 mL) in a 23 mL vial. The mixture was magnetically stirred and preheated in an oil bath at 110 °C for 20 min, after which the temperature was rapidly increased to 195 °C within 20 min. Subsequently, 14 mL of precursor solution containing five metal precursors (Na_2_PdCl_4_, K_2_PtCl_4_, H_2_IrCl_6_ xH_2_O, RuCl_3_ xH_2_O, and RhCl_3_ xH_2_O) dissolved in EG was prepared at an equimolar concentration. The precursor concentration was slightly adjusted for each type of seeds (cubes: 0.048 μmol mL^–1^; octahedra: 0.059 μmol mL^–1^; and icosahedra: 0.044 μmol mL^–1^) to deposit approximately 4–5 atomic layers of the HEA shells. Using a syringe pump, the precursor solution was added dropwise to the preheated mixture at a controlled rate. The injection rates were adjusted according to the seed morphology, with 0.8, 0.7, and 0.6 mL h^–1^ used for cubic, octahedral, and icosahedral seeds, respectively. Following the addition, the reaction mixture was maintained at 195 °C for an additional 2 h. The Pd@Pd_0.2_Pt_0.2_Ir_0.2_Ru_0.2_Rh_0.2_ core–shell nanocrystals were purified by centrifugation, washed once with acetone and twice with DI water, and eventually dispersed in 1 mL of DI water for further use. The preparation of other alloy compositions (Pt_0.33_Ir_0.33_Ru_0.33_, Ir_0.5_Ru_0.5_, Pt_0.5_Ru_0.5_, Pt, and Ru) was carried out following the similar deposition growth procedure described above, except for variations in the types and amounts of the metal precursors.

### Synchrotron X-ray Absorption Spectroscopy (XAS)

XAS measurements were conducted at TPS 44A, TLS 01C1 and TLS 17C1 beamlines of National Synchrotron Radiation Research Center (NSRRC) in Hsinchu, Taiwan. The spectra were acquired using a customized three-electrode cell fabricated from Teflon and equipped with an X-ray transparent window sealed with Kapton film, allowing measurements in total fluorescence yield (TFY) mode. For *in situ* XAS measurements, the experimental procedures were similar to those used for the alkaline HOR electrochemical measurements described above. The collected data were processed using Athena software for calibration, normalization and spectral analysis at the Ru, Rh and Pd K-edge and the Ir and Pt L_3_-edge. FT-EXAFS analysis was performed by Fourier transforming the k^2^-weighted EXAFS oscillations to probe the local coordination environments. The resulting spectra were then fitted using Artemis software to extract detailed structural parameters. The FT-EXAFS fitting was performed over element-specific k ranges of 3–10 Å^–1^ (Pd), 3–11.5 Å^–1^ (Pt and Ru), 3–8.8 Å^–1^ (Ir), and 3–11 Å^–1^ (Rh). The R-space fitting windows were set to 1.6–3.1 Å (Pd), 1.6–3.0 Å (Pt), 1.4–3.2 Å (Ir), and 1.5–3.0 Å (Ru and Rh) in both the Pd@Pd_0.2_Pt_0.2_Ir_0.2_Ru_0.2_Rh_0.2_ core–shell nanooctahedra and the corresponding metallic foils.

### Synchrotron Ambient-Pressure X-ray Photoelectron Spectroscopy (AP-XPS)

*In situ* AP-XPS measurements were carried out at the TLS 24A1 beamline of NSRRC using an electrochemical constant-flow electrolyte cell integrated with an AP-XPS analysis chamber, as reported previously.^83^ For sample preparation, Pd@Pd_0.2_Pt_0.2_Ir_0.2_Ru_0.2_Rh_0.2_ core–shell nanooctahedra were first deposited onto an anion exchange membrane (AEM), followed by transfer of a single-layer graphene film onto the catalyst-coated membrane to improve electrical conductivity. After the sample was mounted into the chamber as working electrode, electrochemical measurements were performed using an Ag/AgCl electrode and a Pt wire as the reference and counter electrodes, respectively, in H_2_-saturated 0.1 M KOH electrolyte. The H_2_-saturated electrolyte was continuously circulated through the cell using a peristaltic pump, allowing dissolved H_2_ to reach the catalyst/AEM interface through the hydrated membrane. The chamber was maintained at approximately 0.3 mbar H_2_O vapor to preserve membrane hydration. The potential was controlled using a Bio-Logic SP-150e potentiostat. Before spectroscopic measurements, the electrode was preconditioned by cyclic voltammetry to stabilize the surface state, and the open-circuit potential (OCP) was recorded after stabilization. *In situ* XPS spectra were acquired at a pressure of 0.3 mbar with a photon energy of 700 eV, while the electrode potential was held at OCP, 0.05, and 0.6 V_RHE_ for 2 h at each step, followed by a final measurement at OCP. This sequence allowed the surface chemical states of the catalyst to be monitored under applied bias. The spectra were fitted using a procedure similar to that used for conventional XPS analysis (see the Supporting Information for details). Owing to the experimental constraints associated with *in situ* AP-XPS measurements, the absolute binding energy positions were not used as the primary basis for comparison. The full width at half maximum (FWHM) of each component was allowed to vary within a limited range to maintain physically reasonable peak shapes, and variations in FWHM under different applied potentials were not assigned specific chemical significance. Binding energy calibration here was performed by setting the Au 4f_7/2_ peak to 84.0 eV.^84^

### Density Functional Theory (DFT) Calculations

This project used DFT to explore the hydrogen and hydroxyl adsorption properties. All DFT calculations were performed using the Vienna Ab initio Simulation Package (VASP).^85^ The projector augmented wave (PAW) method was used to describe the core-electron interactions,^86^ and the RPBE functional from the generalized gradient approximation (GGA) class was employed for the exchange–correlation interaction.^87^ The electronic wave functions were expanded in a plane-wave basis set with energy cutoff (ENCUT) of 500 eV and a Monkhorst–Pack *k*-point mesh of 5 × 5 × 1 was employed for structural relaxation. The electronic energy convergence criterion (EDIFF) was set to 1 × 10^–5^ eV, and geometry optimizations were continued until the residual forces acting on each atom (EDIFFG) were less than 0.02 eV/Å. The HEA surfaces were modeled using four-atomic-layer periodic slab models with a 15 Å vacuum layer along the *z*-direction to avoid artificial periodic interactions. During structural relaxations, the bottom layer was fixed at its bulk positions, while the other layers and the adsorbates were allowed to fully relax. The adsorption Gibbs free energies of hydrogen (ΔG_H*_) and hydroxyl (ΔG_OH*_) species were evaluated using the following expressions

$$\Delta G_{H^{*}}=E_{{slab+H}^{*}}-E_{slab}-{\frac{1}{2}E}_{H_{2}}+0.24\left(eV\right)$$

$$\Delta G_{{OH}^{*}}=E_{{slab+OH}^{*}}-E_{slab}-\left(E_{H_{2}O}-\frac{1}{2}E_{H_{2}}\right)+0.29\left(eV\right)$$

where E_slab+H*_ and E_slab+OH*_ represent the DFT total electronic energies of the HEA slab with H* and OH* adsorbed, respectively. E_slab_ is the electronic energy of the clean slab, and E_H2_ and E_H2O_ are the electronic energies of isolated gas-phase H_2_ and H_2_O molecules calculated in a large non-periodic box.

For H* and OH* adsorption, the Gibbs free energy corrections were derived by incorporating zero-point energy and entropy contributions based on previously reported values from separate literature sources.^18,88,89^ For more detailed information, please refer to the Computational section in the Supporting Information.

### Climbing-Image Nudged Elastic Band (CI-NEB) Calculations

The transition states (TS) and minimum energy pathways (MEP) for the surface diffusion and coupling reaction steps were determined using the CI-NEB method implemented within VASP.^90–92^ The core computational parameters, including the PAW potentials, plane-wave cutoff energy (500 eV), and electronic/ionic convergence criteria (EDIFF/EDIFFG), were kept consistent with those used in the structural optimizations described above. To map the reaction coordinates, 3 or 5 intermediate images were generated via linear interpolation between the optimized initial and final states based on their geometric distances. A spring constant (SPRING) of −5 eV/Å^2^ was applied to ensure equidistant spacing between adjacent images along the elastic band. Brillouin zone integration for all CI-NEB calculations was performed using Gamma-centered 4 × 4 × 1 *k*-point mesh. A slightly reduced *k*-point density compared with the structural optimization calculations was adopted to balance computational efficiency while maintaining reliable relative energy differences along the reaction pathways. The activation barrier was determined from the potential energy profile along the reaction coordinate obtained from CI-NEB calculations. For each intermediate image, the relative energy (Δ*E*) was calculated with respect to corresponding initial state (INI) as

$$\Delta E=E-E_{INI}$$

Also the energy barrier for each elementary reaction step was then evaluated as the energy difference between the TS and the INI.

## Supplementary Material



## Notes

Materials and Methods, XRD, TEM, XPS, XAS, electrochemical analyses, and DFT calculations (PDF)
