# Supplementary material for: Surface Atomic Arrangements on High-Entropy-Alloy Nanocrystals Govern Alkaline Hydrogen Oxidation via Symmetry-Selected Multi-Atom Ensembles
Source: J Am Chem Soc. 2026 Sep 4;148(43):46825–45. doi: 10.1021/jacs.6c13325 (PMC13741843; doi:10.1021/jacs.6c13325)
Supplement: Supplementary file 1 [file ja6c13325_si_001.pdf]

Supporting information

Surface Atomic Arrangements on High-Entropy-Alloy Nanocrystals Govern Alkaline Hydrogen Oxidation *via* Symmetry-Selected Multi−Atom Ensembles

Kuan-Fang Lee,[1,§] Liang-Yu Hou,[2,§] Yueh-Chun Hsiao,[1] Chia-Ying Wu,[1] Yun-Shan Tsai,[1] Chun-Wei Chang,[1] Jui-Tai Lin,[1] Shang-Cheng Lin,[1] Cheng-Yu Wu,[1] Zong-Ying He,[2] Yi Chen,[1] Shu-Han Lee,[3] Chia-Hsin Wang,[3] Chih-Wen Pao,[3] Kun-Han Lin,[1] and Tung-Han Yang[1,2,4,*]

[1]Department of Chemical Engineering, National Tsing Hua University, Hsinchu 300044, Taiwan.

[2]College of Semiconductor Research, National Tsing Hua University, Hsinchu 300044, Taiwan.

[3]National Synchrotron Radiation Research Center, Hsinchu 300092, Taiwan.

[4]High Entropy Materials Center, National Tsing Hua University, Hsinchu 300044, Taiwan.

[§]Kuan-Fang Lee and Liang-Yu Hou contributed equally to this work.

*Corresponding author. Email: tunghanyang@mx.nthu.edu.tw

The file includes: Materials and Methods, Figures S1 to S31, Tables S1 to S6, and References.

Materials and Methods

Chemicals

Sodium tetrachloropalladate(II) (Na_2PdCl_4), potassium tetrachloroplatinate(II) (K_2PtCl_4), hydrogen hexachloroiridate(IV) hydrate ($H_2IrCl_6 \cdot xH_2O$), ruthenium(III) chloride hydrate ($RuCl_3 \cdot xH_2O$), rhodium(III) chloride hydrate ($RhCl_3 \cdot xH_2O$), poly(vinylpyrrolidone) (PVP, Mw ≈ 55,000), L-ascorbic acid (AA), potassium bromide (KBr), triethylene glycol (TEG), formaldehyde (CH_2O), 5 wt% Nafion solution were all obtained from Sigma-Aldrich. Ethylene glycol (EG) and hydrochloric acid (HCl) were purchased from J.T. Baker. Acetone (CH_3COCH_3) and potassium hydroxide (KOH) were obtained from Duksan. Acetic acid (CH_3COOH) and isopropanol ($(CH_3)_2CHOH$) were bought from Honeywell. Deionized (DI) water with a resistivity of 18.2 MΩ·cm was used for all the experiments.

Synthesis of Pd cubic, octahedral and icosahedral seeds with controlled facets

Pd cubic seeds with an average size of approximately 18.0 nm were synthesized using a typical protocol.[1, 2] 8 mL of DI water, 105 mg of PVP, 600 mg of KBr, and 60 mg of AA were placed in a 23-mL vial and preheated at 80 °C oil bath for 10 minutes with magnetic stirring. A one-shot injection of 3 mL of Na_2PdCl_4 aqueous solution at a concentration of 19 mg mL^{-1} was added to the vial. The vial was sealed with a cap and heated at 80 °C for 3 hours under continuous magnetic stirring. After cooling to room temperature, the product was purified by centrifugation with three washes using DI water, and finally collected in EG. For the synthesis of 12.0-nm Pd cubic seeds, only the amount of KBr was reduced to 300 mg, whereas all other conditions remained identical to those described above; however, this product was dispersed in DI water.

To prepare Pd octahedral seeds with an average size of approximately 18.0 nm, a previously reported standard synthesis protocol was employed.[3] 8.1 mL of DI water, 0.1 mL of HCHO, and 0.2 mL of a suspension of the 12.0-nm Pd cubic seeds were introduced into a 23-mL vial, followed by

stirring and preheating in an oil bath at 60 °C for 10 minutes. Subsequently, 3 mL of Na_2PdCl_4 aqueous solution at a concentration of 12.57 mg mL^{-1} was injected into the vial. The vial was sealed with a cap and maintained at 60 °C for 3 hours with continuous magnetic stirring. The mixture was allowed to cool to room temperature. The product was purified by centrifugation, washed three times with DI water, collected, and finally redispersed in EG.

To obtain the Pd icosahedral seeds, the preparation followed a method based on previous work.[4] 2 mL of TEG and 80 mg of PVP were placed in a 23-mL vial and preheated in an oil bath at 130 °C for 20 minutes. The precursor solution containing 15.5 mg of Na_2PdCl_4, 1 mL of TEG, and 2.5 μL of HCl (37.7%) was prepared and then added to the vial in one-shot. The vial was capped and maintained at 130 °C for 3 hours under magnetic stirring. After the reaction was complete, the vial was immediately immersed in an ice-water bath to cool the mixture. The product was collected by centrifugation, washed once with acetone and twice with DI water, and finally redispersed in EG.

Calculation of the ratio of the surface atom and total atom number of core-shell nanocrystals

Case 1: core-shell nanocube

The Pd nanocubes were assumed to maintain a perfect cubic shape after seed-mediated epitaxial growth of the targeted metal layers (Figure 1). Based on this assumption, the total number of atoms in the Pd cubic seed and the number of atoms in the shell (n layers) of each core-shell nanocube could be calculated through the equations[2]:

$$\text{The total number of atoms in the Pd cubic seed} = 4 * \left(\frac{l^3}{A^3}\right)$$

$$\text{The total number of atoms in the shell (n layers)} = \sum_{L=1}^{n} \left\{ \frac{6 * 2 * (l + a + L)^2}{a^2} \right\}$$

In these equations, l is the edge length of the Pd nanocube, A is the lattice constant of Pd (0.389 nm), and a is the lattice constant of the targeted metal layers.

Case 2: core-shell nanooctahedron

As shown in Figure 1, the Pd nanooctahedra were assumed to retain a truncated octahedral shape after the seed-mediated growth procedure, similar to the case described above. To quantitatively describe the truncated octahedral geometry, two parameters were defined: n_l and n_{cut}. n_l denotes the number of atoms along the edges of a complete octahedron, while n_{cut} represents the number of atomic layers removed relative to a perfect octahedron. Based on this model, both the total number of atoms in the Pd octahedral seed and the number of atoms in each shell layer (n layers) of the truncated core-shell octahedra were calculated using the equations[5]:

$$\text{The total number of atoms in the Pd octahedral seed} = \frac{1}{3}\left(2n_l^3 + n_l\right) - 2n_{cut}^3 - 3n_{cut}^2 - n_{cut}$$

$$\text{The total number of atoms in the shell (n layers)} =$$

$$\sum_{L=1}^{n}\left\{6[(n_{cut}+1)+2L]^2 + 8\left\{\frac{1}{2}[1+(n_l+2L)]*(n_l+2L) - \frac{3}{2}[1+(n_{cut}+2L)]*(n_{cut}+2L)\right\} - 24[(n_{cut}+1)+2L] - 12[(n_l-2n_{cut})+2L]\right\}$$

Case 3: core-shell nanoicosahedron

The Pd icosahedra were assumed to remain unchanged icosahedral shape following the layer-by-layer epitaxial growth of the targeted metal layers as shown in Figure 1. Under this assumption, the total number of atoms and the number of atoms in the shell (n layers) for each core-shell nanoicosahedron were estimated by the equations[6, 7]:

$$\text{The total number of atoms} = \frac{10}{3}L^3 + 5L^2 + \frac{11}{3}L + 1$$

$$\text{The total number of atoms in the shell (n layers)} = \sum_{L=62}^{62+2n}(10L^2 + 2)$$

Here, L represents the number of atoms along the edge of the Pd icosahedron. Using the Pd interplanar spacing of 0.225 nm, the edge length of a 26.85-nm Pd icosahedron was determined to be 14.12 nm, corresponding to 62 Pd atoms.

Based on the above equations together with the nanocrystals sizes, the total number of atoms in the core-shell nanocrystals and the total number of atoms in the shell for each case can be obtained. The ideal atomic percentage (at%) of the shell in core-shell nanocrystals was calculated based on the equation:

$$\text{The ideal atomic percentage of the shell in core-shell nanocrystals} = \frac{Total\ number\ of\ atoms\ in\ the\ shell\ (n\ layers)}{Total\ number\ of\ atoms\ in\ the\ seed + Total\ number\ of\ atoms\ in\ the\ shell\ (n\ layers)}$$

For core-shell nanoicosahedron, the denominator can be directly expressed as the total number of atoms.

This approach was then used to estimate the ideal atomic percentage corresponding to each shell thickness, and the experimental atomic percentages of the synthesized core-shell nanocrystals were calculated from the ICP-OES data. The approximate shell thickness, in terms of number of atomic layers, was then determined by comparing the theoretical and experimental atomic percentages listed in Table S2.

Preparation of the working electrode for electrochemical measurements

In a standard procedure, 1 mg of $Pd@Pd_{0.2}Pt_{0.2}Ir_{0.2}Ru_{0.2}Rh_{0.2}$ core-shell nanocrystals dispersed in 2 mL of DI water and 2 mg of carbon support (Vulcan XC-72R) suspended in 8 mL of DI water were introduced into a capped 23-mL vial, and then mixed under ultrasonication at room temperature for 3 hours. The mixture was centrifuged to remove DI water, followed by the addition of 3 mL of acetic acid and heating at 60 °C for 3 hours to ensure surface cleanliness.[8, 9] To thoroughly eliminate residual acetic acid, the sample was washed three times with DI water and three times with ethanol by centrifugation, and subsequently dried at 60 °C for 6 hours. After drying, 1.0 mg of sample was redispersed in 0.5 mL of DI water, 0.5 mL of isopropanol, and 20 μL of Nafion (5 wt%) solution, followed by ultrasonication for 15 minutes to obtain a homogeneous ink. Subsequently, 3.6 μL of the catalyst suspension was deposited onto a glassy carbon rotating disk electrode (RDE) with a geometric

area of 0.07 cm^2 and dried under an isopropanol-saturated atmosphere for 12 hours. For a fair comparison, the commercial Pt/C (20%) served as a benchmark. The preparation process was similar to that described above. 1 mg of commercial Pt/C (20%) was dispersed in 0.5 mL of DI water, 0.5 mL of isopropanol, and 20 μL of Nafion (5 wt%) solution, followed by ultrasonication for 15 minutes. Afterwards, 3.6 μL of the catalyst suspension was deposited onto a glassy carbon RDE and dried under an isopropanol-saturated atmosphere for 12 hours.

Electrochemical measurements

The electrochemical measurements were conducted using a Metrohm Autolab PGSTAT204 Nova Potentiostat as the electrochemical workstation. A catalyst-coated glassy carbon electrode was used as the working electrode, while a platinum electrode and an Hg/HgO electrode served as the counter and reference electrodes, respectively. The measured potentials referenced to the Hg/HgO electrode were calibrated to the reversible hydrogen electrode (RHE) according to the following equation: $V_{RHE} = V_{measured} + 0.059pH + 0.118$. Prior to electrochemical measurements, the 0.1M KOH electrolyte was purged with N_2 gas for 30 minutes. To ensure the cleanliness of the electrode surface, 40 cycles of cyclic voltammetry (CV) were performed at a scan rate of 500 mV s^{-1} within a potential window of 0.05 to 1.1 V_{RHE}. Subsequently, for alkaline HOR electrocatalytic measurements, the 0.1M KOH electrolyte was pretreated by purging with H_2 gas for 30 minutes. CV and linear sweep voltammetry (LSV) were employed at a rotation speed of 1600 rpm without iR compensation. CV curves were recorded within a potential range of –0.1 to 0.7 V_{RHE} at a scan rate of 10 mV s^{-1}, followed by LSV at a scan rate of 10 mV s^{-1} over the same potential range.

To investigate the effect of H_2 transport on performance, LSV measurements were conducted at rotation speeds of 400, 625, 900, 1225, 1600, 2025, and 2500 rpm, individually. The inverse square root of the rotation rate ($\omega^{-1/2}$) was linearly fitted against the corresponding inverse current density. The resulting slope was compared with theoretical Koutecky-Levich values to assess the influence of H_2 mass transport on the alkaline HOR.

To estimate long-term durability, a sequential stability test was conducted using the procedure described above. The catalyst was subjected to 3000 cycles of CV within the same potential range at a scan rate of 150 mV s^{-1}. Following this, 40 cycles of CV were performed at a scan rate of 500 mV s^{-1} between 0.05 and 1.1 V_{RHE} for electrode surface refreshing. CV curves were then recorded at a scan rate of 10 mV s^{-1} over the potential range of –0.1 to 0.7 V_{RHE}, and the resulting data were defined as the 3000th cycle. The same procedure was repeated to obtain CV curves at the 6000th cycle. The CV curves recorded at the 1st, 3000th, and 6000th cycles were compared to assess the catalytic durability.

The kinetic current density (J_k) was calculated by the Koutecky-Levich equation: $\frac{1}{J} = \frac{1}{J_k} + \frac{1}{J_d}$, where J is the measured current density, and J_d is the diffusion current density.

The exchange current density (j_0) was estimated from the slope of the linear fitting of kinetic current density versus overpotential in the low-overpotential range, according to the approximate Butler-Volmer equation: $j_0 = \frac{J}{\eta} \cdot \frac{RT}{nF}$, where $\frac{J}{\eta}$ is the slope of the linear fitting, where R is the gas constant (8.314 J $K^{-1}mol^{-1}$), T is the temperature (assumed to be room temperature, 298.15 K), F is the Faraday constant (96485 C mol^{-1}), and n is the number of electrons participating in the reaction (n = 2 for alkaline HOR).

The Tafel slopes were obtained by fitting the linear region of the Tafel plots derived from the alkaline HOR polarization curves. Tafel plots were constructed by plotting the overpotential (η) versus the logarithm of current density (log|J|), where η represents the overpotential relative to the equilibrium potential of HOR (0 V vs. RHE). Linear fitting of the Tafel region was performed to determine the Tafel slopes.

Evaluation of electrochemical surface area (ECSA) based on double-layer capacitance

The double-layer capacitance (C_{dl}) is used to determine the ECSA of the catalysts.[10] To measure the C_{dl}, CV measurement was performed in N_2-saturated 0.1 M KOH electrolyte. CV curves were recorded within a potential window of ±0.05 V around the open circuit potential (OCP) at various scan

rates of 25, 50, 100, 150, 200, 300, 400, and 500 mV s^{-1}. Subsequently, the anodic and cathodic current densities were extracted from the CV curves to calculate the capacitive current ($\Delta j = j_{anodic}$-$j_{cathodic}$). The C_{dl} was obtained from the slope of the linear fit of $\Delta j/2$ as a function of scan rate. The ECSA was then calculated using the following equation: $\mathrm{ECSA} = C_{dl}/C_s$, where C_s represents an assumed specific capacitance value of 40 µF cm^{-2}, commonly adopted for mixed platinum-group metal surfaces in 0.1 M KOH.[11, 12]

Hydrogen underpotential deposition (HUPD) and CO-stripping analyses

The hydrogen underpotential deposition experiments were employed by recording CV curves within a potential range from 0.05 to 1.1 V_{RHE} at a scan rate of 50 mV s^{-1} in N_2-saturated 0.1 M KOH electrolyte. The potential of the peak (E_{peak}) observed from 0.05 to 0.4 V_{RHE} was used to represent the hydrogen binding energy for each sample by using the equation: $\Delta H = -FE_{peak}$.[13, 14]

The CO-stripping experiments were carried out in 0.1 M KOH electrolyte, which was pre-saturated with CO by purging CO gas into the electrolyte for 30 minutes prior to the measurements. The electrode was initially held at a constant potential of 0.1 V_{RHE} for 10 minutes to promote CO adsorption on the catalyst surface. Afterward, the electrolyte was purged with N_2 gas for 20 minutes to remove the residual CO, which prevented interference with subsequent measurements. CV curves were then conducted with the potential window of 0 to 1.2 V_{RHE} for two continuous cycles at a scan rate of 50 mV s^{-1}. The first CV cycle exhibited a CO-stripping peak arising from the electrooxidation of adsorbed CO, whereas the second CV cycle confirmed the complete removal of CO from the catalyst surface and served as the baseline for the first cycle.

X-ray photoelectron spectroscopy (XPS) peak-fitting analysis

XPS spectra were analyzed with XPSPEAK41 software. Peak deconvolution was performed using a Shirley background and mixed Gaussian-Lorentzian functions, with theoretical constraints applied to the intensity ratios and energy separations of spin-orbit split peaks. In particular, the area

ratios were fixed at 2:1 for Ru $3p_{3/2}$ to $3p_{1/2}$, 3:2 for Pd and Rh $3d_{5/2}$ to $3d_{3/2}$, and 4:3 for Pt and Ir $4f_{7/2}$ to $4f_{5/2}$.[15] For each chemical state, the corresponding spin-orbit components were constrained to have identical full width at half maximum (FWHM) values. All binding energies were calibrated to the C 1s peak at 284.5 eV. The fitted binding energies were cross-validated against literature-reported values for common chemical states.[15] Because XPSPEAK41 software does not provide covariance-matrix-derived statistical uncertainties for fitted peak areas, formal fitting error bars could not be directly extracted. To improve the robustness and physical consistency of the fitting, the spin-orbit splitting, peak-area ratios, and FWHM ranges were constrained before iterative optimization, and the fitting was continued until the $\Sigma\chi^2$ value converged. Accordingly, the fitted component fractions should be interpreted as chemically reasonable relative trends rather than as absolute quantitative measures with independently determined statistical uncertainties.

For the *in situ* AP-XPS analysis, particular caution was applied to the Ru 3d region because of its partial overlap with the adventitious C 1s signal. Although the Ru 3p region was initially considered to avoid this overlap, its substantially weaker photoelectron intensity under the liquid-cell AP-XPS conditions prevented reliable analysis. The stronger Ru 3d signal was therefore used, with the above fitting constraints applied consistently to separate the Ru 3d and C 1s contributions. Consequently, the potential-dependent Ru^0/Ru^{4+} ratios are used primarily to evaluate relative chemical-state trends rather than as precise quantitative measures of surface coverage. Binding energy calibration for conventional XPS spectra was performed using the C 1s peak at 284.5 eV, whereas the *in situ* AP-XPS spectra were calibrated separately as described in the corresponding experimental section.

Characterizations

The element compositions of the samples were measured by inductively coupled plasma optical emission spectroscopy (ICP-OES) (Thermo SCIENTIFIC, ICAP 7200 Duo). Transmission electron microscopy (TEM) images were obtained using a transmission electron microscope (JEOL, JEM-2100) operated at 200 kV. High-angle annular dark-field scanning TEM (HAADF-STEM) and energy-

dispersive X-ray spectroscopy (EDS) images were acquired using a spherical-aberration-corrected field-emission TEM (JEOL, JEM-ARM200FTH) operated at 200 kV. The crystalline structure of the nanocrystals was characterized by X-ray diffraction (XRD) using an X-ray diffractometer (Bruker, D8). The surface chemical states were analyzed by X-ray photoelectron spectroscopy (XPS) using a ULVAC-PHI Quantera II high-resolution spectrometer.

Computational section

The PdPtIrRuRh high-entropy alloy (HEA) surfaces were modeled using periodic slab structures constructed as 4 × 4 supercells with four atomic layers. Both (100) and (111) crystallographic surfaces were considered to evaluate facet-dependent adsorption properties. To ensure a consistent basis for comparison, the total number of atoms in the (100) and (111) slab models was kept identical. A vacuum layer of 15 Å was introduced along the surface-normal direction to eliminate spurious interactions between periodic images. During structural optimization, only the bottom atomic layer was fixed to mimic bulk constraints, while the remaining layers and all adsorbates were fully relaxed. To account for the configurational diversity of the HEA surfaces, 12 independently randomized {100} slab models and 19 independently randomized {111} slab models were generated using the Atomic Simulation Environment (ASE) (Figures S24 and S25). For each model, the atomic identities of Pd, Pt, Ir, Ru, and Rh were randomly shuffled among the lattice sites while keeping the number of atoms of each element fixed according to the prescribed near-equimolar composition based on the experimentally determined TEM and ICP-OES results. All models retained the same slab size and thickness, thereby allowing the effects of different local elemental configurations to be evaluated on a consistent structural basis. This procedure generated a broad set of surface configurations for subsequent adsorption and reaction-energy calculations.

To systematically sample the adsorption configurations, H^* and OH^* adsorbates were initially placed approximately 1.5–2.0 Å above representative high-symmetry sites distributed across each

HEA surface. Hollow sites were primarily used as the initial positions for the {111} surfaces, whereas both on-top and hollow sites were considered for the {100} surfaces. These positions served only as starting geometries and did not impose predefined adsorption-site assignments. During subsequent DFT geometry optimization, both the adsorbates and unconstrained surface atoms were allowed to relax, enabling H^* and OH^* to migrate toward locally stable adsorption configurations. The final adsorption sites were therefore classified based on the fully relaxed structures rather than their initial positions. For each {100} slab, the surface lattice contains 16 on-top, 32 bridge, and 16 hollow sites, whereas each {111} slab contains 16 on-top, 48 bridge, and 32 hollow sites. Representative surface regions were sampled across multiple independently randomized HEA slabs to cover a broad range of geometrically distinct adsorption sites and local elemental environments. The frequencies of on-top, bridge, and hollow sites were then determined from the final relaxed adsorption structures. The corresponding adsorption energies and site distributions of H^* and OH^* are summarized in Figures 7, S26, S27, and Tables S5 and S6.

The adsorption free energy was calculated based on the concept of computational hydrogen electrode (CHE):

$$\Delta G_{ads} = E_{slab+adsorbate} - E_{slab} - E_{adsorbate} + \Delta E_{ZPE} - T\Delta S$$

where $E_{slab+adsorbate}$, E_{slab}, and $E_{adsorbate}$ denote the total energies of the adsorbate-bound slab, clean HEA surface, and isolated reference species, respectively. The Gibbs free energy correction was obtained by including zero-point energy and entropy contributions following previously reported methodologies and reference data.[16-18]

Radial distribution function (RDF) analysis

To elucidate how the local atomic environment influences the adsorption energetics of reaction intermediates, element-resolved RDF analysis was performed for the equimolar PdPtIrRuRh HEA. The {111} facet was selected because the corresponding core-shell HEA nanooctahedra, which are predominantly enclosed by {111} facets, exhibited the highest alkaline HOR activity. The {111}

surface was therefore used as a representative model to correlate the local coordination environment with the adsorption energetics of H^* and OH^*. For each adsorbate, the spatial distributions of the five constituent metal species (Pd, Pt, Ir, Ru, and Rh) were analyzed separately as a function of radial distance from the adsorption center. For H^* adsorption, the adsorbed H atom was used as the reference center, whereas for OH^* adsorption, the O atom directly coordinated to the surface was selected as the reference center. Previous studies have shown that element-resolved analysis of local atomic environments provides an effective means of relating the identities and spatial distributions of neighboring metal atoms to adsorbate binding on multicomponent alloy surfaces.[19-22] Such analysis can also provide insight into ligand effects, whereby neighboring metal species modify the local electronic environment of the adsorption site and thereby influence the adsorbate–surface interaction. By resolving the contribution of each metal species as a function of radial distance, the RDF analysis enables the relationship between the surrounding multimetallic environment and the adsorption strengths of H^* and OH^* to be statistically evaluated.

The resulting frequency distribution value, denoted as $g_{H\text{-}M}(r)$ and $g_{O\text{-}M}(r)$, describes the relative occurrence of a specific metal species (M) at a radial distance (r) from the corresponding adsorbate. The radial distributions were calculated using a sphere-based algorithm with a cutoff radius of 5.6 Å, corresponding to half of the simulation cell length and thereby satisfying the minimum image convention. The radial distance was discretized into bins with a width (δr) of 0.05 Å. For a given metal species (M) and adsorbate configuration within a specified adsorption free-energy range, the radial distribution value was obtained by counting the number of metal atoms ($N_{count}(r)$), located within a spherical shell centered at distance (r). The resulting count was normalized by both the volume of the spherical shell ($V_{shell}(r)$), and the number density of the corresponding metal species (ρ_M), to minimize geometric and compositional contributions:

$$g_{X-M}(r) = \frac{N_{count}(r)}{V_{shell}(r) \times \rho_M}$$

where X represents H for H^* adsorption and O for OH^* adsorption. The shell volume is defined as:

$$V_{shell}(r) = \frac{4}{3}\pi \left[\left(r + \frac{\delta r}{2}\right)^3 - \left(r - \frac{\delta r}{2}\right)^3\right]$$

Finally, to ensure statistical representativeness, the RDF profiles were averaged over all sampled PdPtIrRuRh {111} configurations within each adsorption free-energy category. This ensemble averaging reduces fluctuations arising from individual atomic configurations and highlights the characteristic Pd-, Pt-, Ir-, Ru-, and Rh-containing coordination environments associated with different H^* and OH^* adsorption strengths. By comparing the element-resolved radial distributions across different adsorption-energy ranges, preferential enrichment of specific metal species within the local coordination environment can be identified, providing statistical insight into how the surrounding multimetallic environment, including possible ligand effects, correlates with H^* and OH^* adsorption relevant to alkaline HOR (Figures S29 and S30).

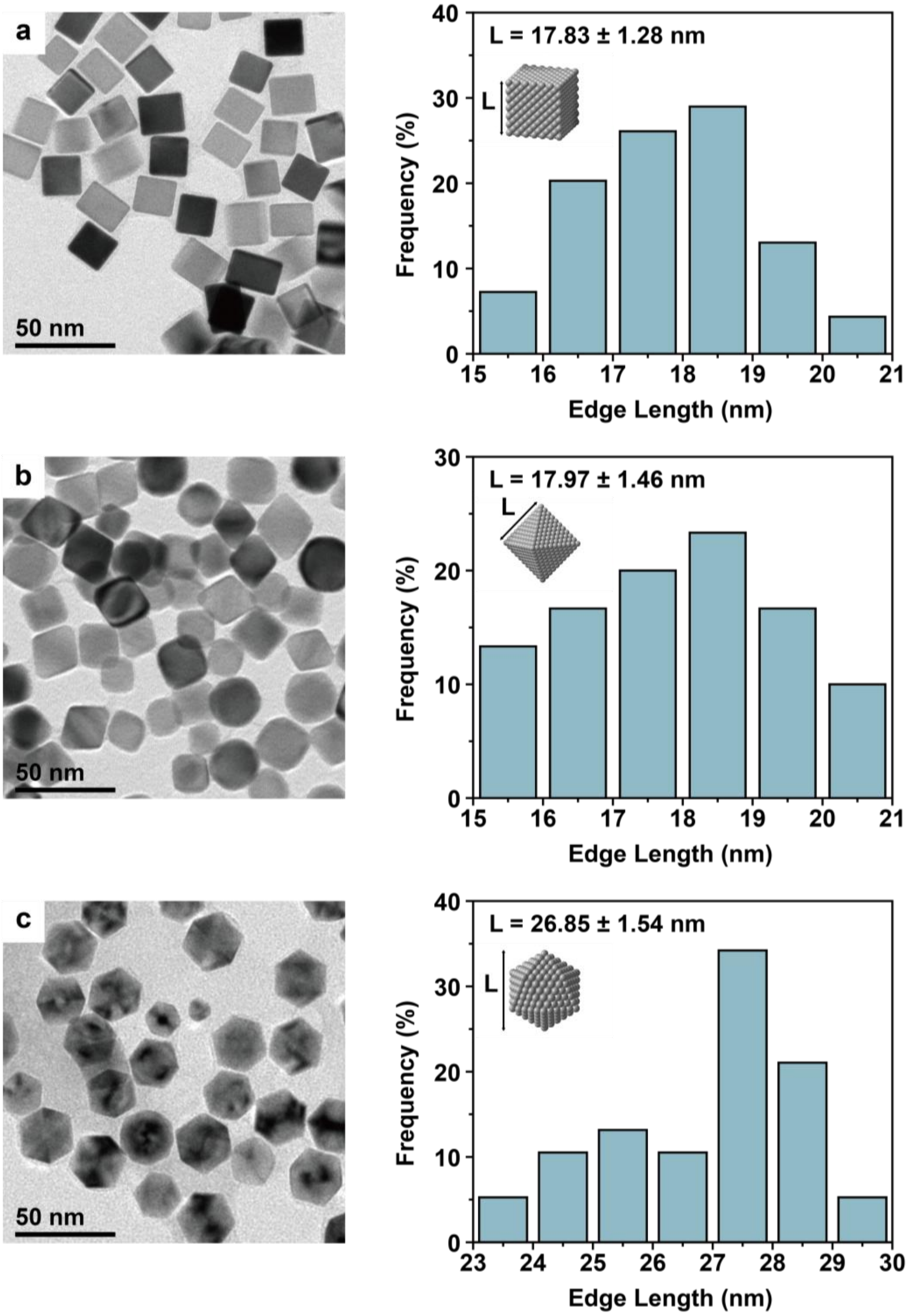

Figure S1. TEM images and edge-length distributions of Pd seeds. (a) Pd nanocubes with an average edge length of 17.83 nm enclosed by {100} facets, (b) Pd nanooctahedra with an average edge length of 17.97 nm enclosed by {111} facets, and (c) Pd nanoicosahedra with an average edge length of 26.85 nm enclosed by twinned {111} facets. The particle size (L) of each seed is defined in the corresponding image.

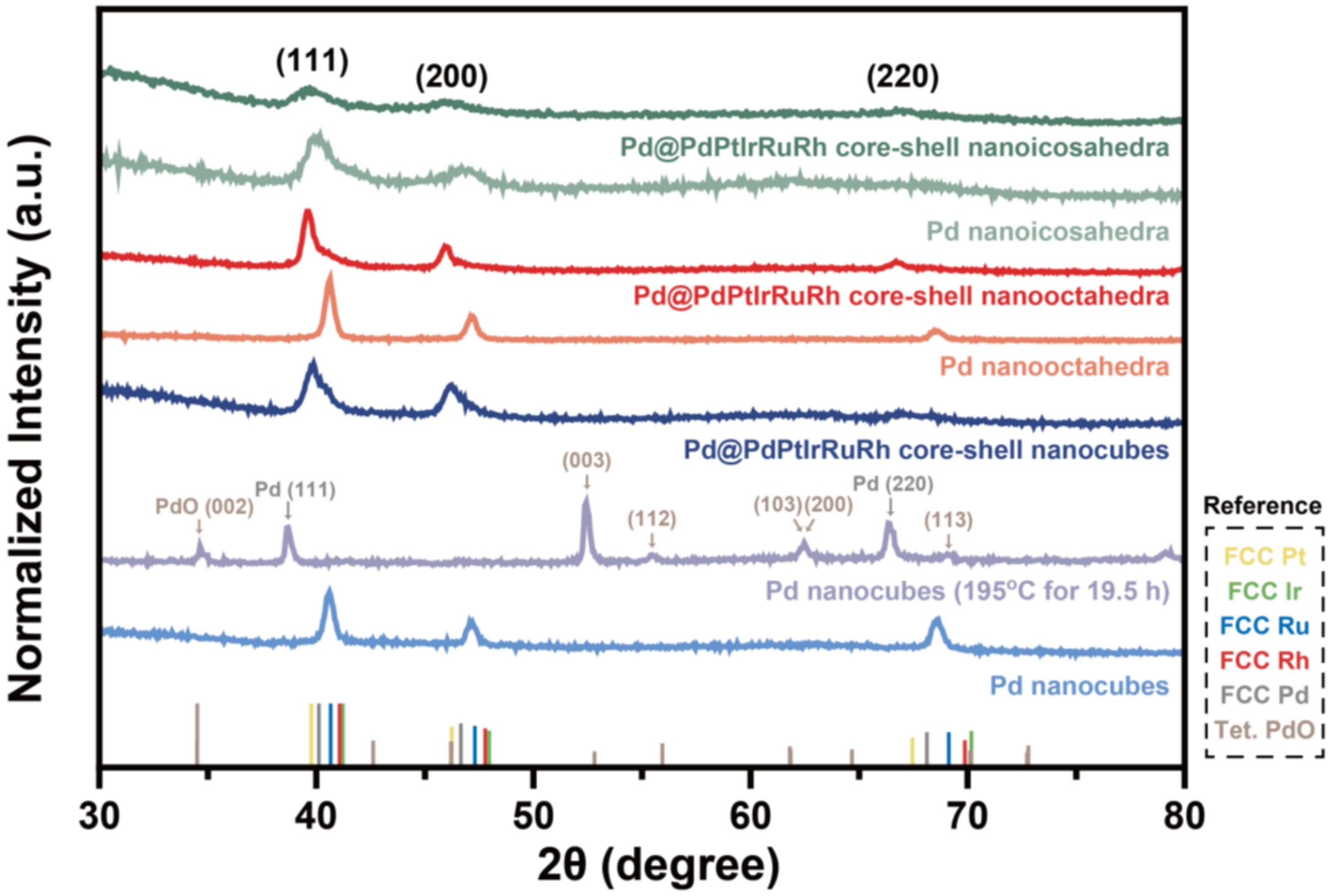

Figure S2. XRD analyses of $Pd@Pd_{0.2}Pt_{0.2}Ir_{0.2}Ru_{0.2}Rh_{0.2}$ core-shell nanocrystals and the corresponding Pd seeds. The XRD patterns of $Pd@Pd_{0.2}Pt_{0.2}Ir_{0.2}Ru_{0.2}Rh_{0.2}$ core-shell nanocubes enclosed by {100} facets, $Pd@Pd_{0.2}Pt_{0.2}Ir_{0.2}Ru_{0.2}Rh_{0.2}$ core-shell nanooctahedra enclosed by {111} facets, and $Pd@Pd_{0.2}Pt_{0.2}Ir_{0.2}Ru_{0.2}Rh_{0.2}$ core-shell nanoicosahedra enclosed by twinned {111} facets exhibit characteristic diffraction peaks that can be indexed to the (111), (200), and (220) planes of the face-centered cubic (FCC) structure. The XRD patterns of the corresponding Pd nanocube, nanooctahedron, and nanoicosahedron seeds are also included for comparison. A control experiment was further performed using representative Pd nanocubes heated at 195 °C for 19.5 h under the same thermal conditions used for HEA-shell growth, but without addition of the multimetallic precursors. In the absence of the HEA shell, partial oxidation of the Pd nanocubes occurs during the prolonged thermal treatment, leading to the appearance of tetragonal (Tet.) PdO-related reflections. The remaining FCC Pd reflections also shift noticeably relative to those of the initial Pd nanocubes. For example, the strongest FCC Pd (111) reflection shifts from approximately 40.6° for the initial Pd nanocubes to 38.7° after thermal treatment, indicating substantial modification of the Pd lattice during

prolonged heating. This lattice change may arise from the combined effects of thermal treatment, partial surface oxidation, and residual Br-containing surface species originating from the Br^--containing capping environment used during the synthesis of the Pd nanocubes. The strongest FCC-related reflection of the$Pd@Pd_{0.2}Pt_{0.2}Ir_{0.2}Ru_{0.2}Rh_{0.2}$ core-shell nanocubes appears at approximately 39.8°, which is also shifted to lower 2θ relative to the initial Pd nanocubes. Although this peak position is not identical to that of the thermally treated Pd control, both samples exhibit the same overall shift toward lower 2θ, indicating that prolonged thermal treatment and subsequent shell growth both influence the Pd-based lattice. The dominant diffraction features of the core-shell nanocrystals are therefore attributed mainly to the structurally evolved Pd-rich core, with additional contributions from the thin HEA shell. Weak shoulder features at slightly higher 2θ values are observed only after growth of the $Pd_{0.2}Pt_{0.2}Ir_{0.2}Ru_{0.2}Rh_{0.2}$ HEA shell and are therefore assigned to diffraction contributions from the thin FCC HEA shell. Their higher 2θ positions are consistent with a smaller average lattice parameter of the alloy shell relative to the Pd-rich core, as qualitatively expected from the incorporation of Pt, Ir, Ru, and Rh into the FCC lattice based on Vegard-type considerations. Overall, the asymmetric diffraction profiles are consistent with overlapping contributions from the structurally evolved Pd-rich core and the thin FCC HEA shell rather than the formation of an additional unidentified bulk crystalline phase. All XRD patterns were normalized to their respective maximum intensities to facilitate comparison of the diffraction peak positions and shoulder features.

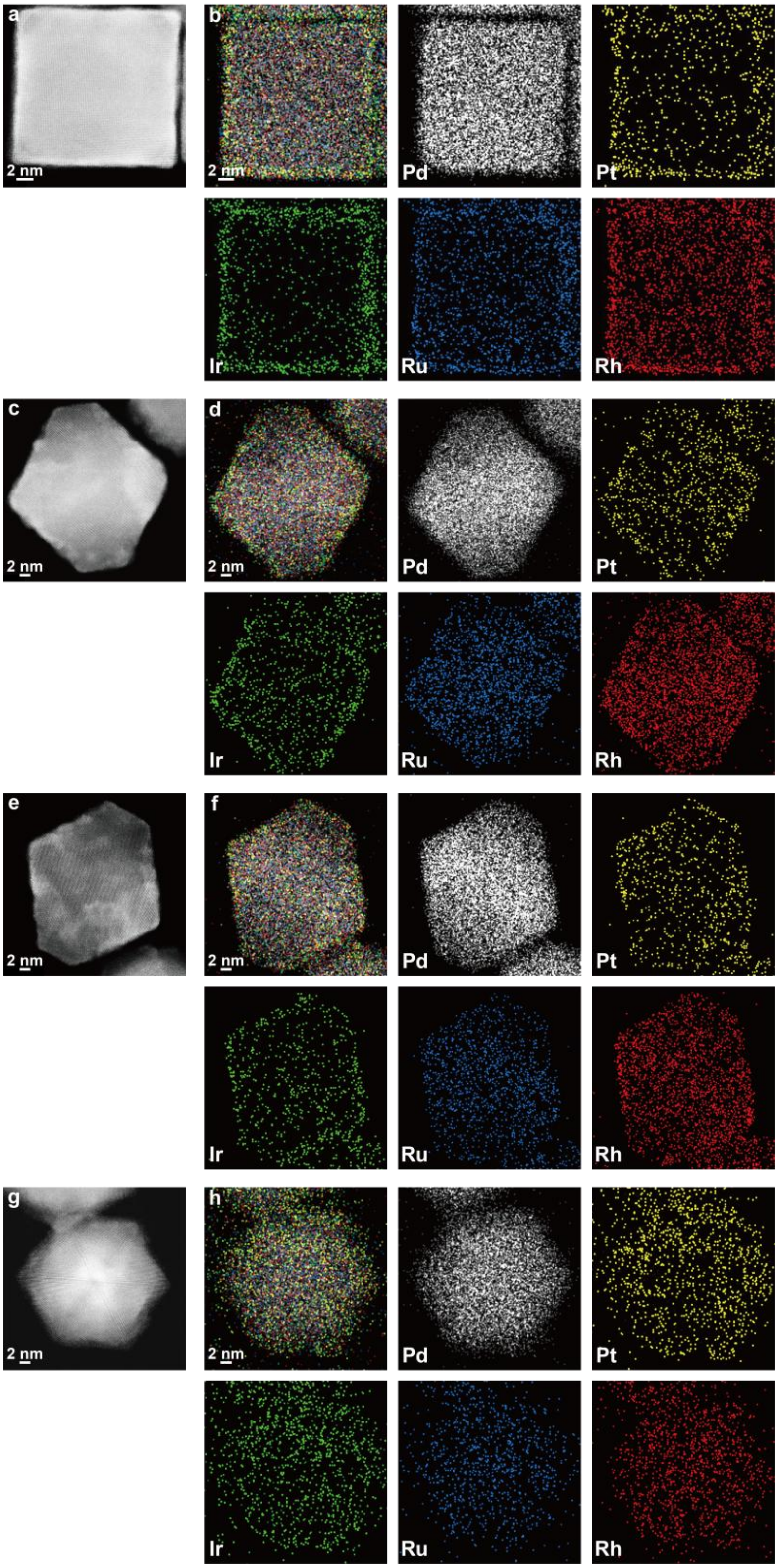

Figure S3. Additional HAADF-STEM images and EDS mapping of $Pd@Pd_{0.2}Pt_{0.2}Ir_{0.2}Ru_{0.2}Rh_{0.2}$ core-shell nanocrystals. (a, b) A core-shell nanocube, (c–f) two core-shell nanooctahedra, and (g, h) a core-shell nanoicosahedron. The EDS elemental maps show the overlaid distribution of all constituent elements together with the individual distributions of Pd, Pt, Ir, Ru, and Rh.

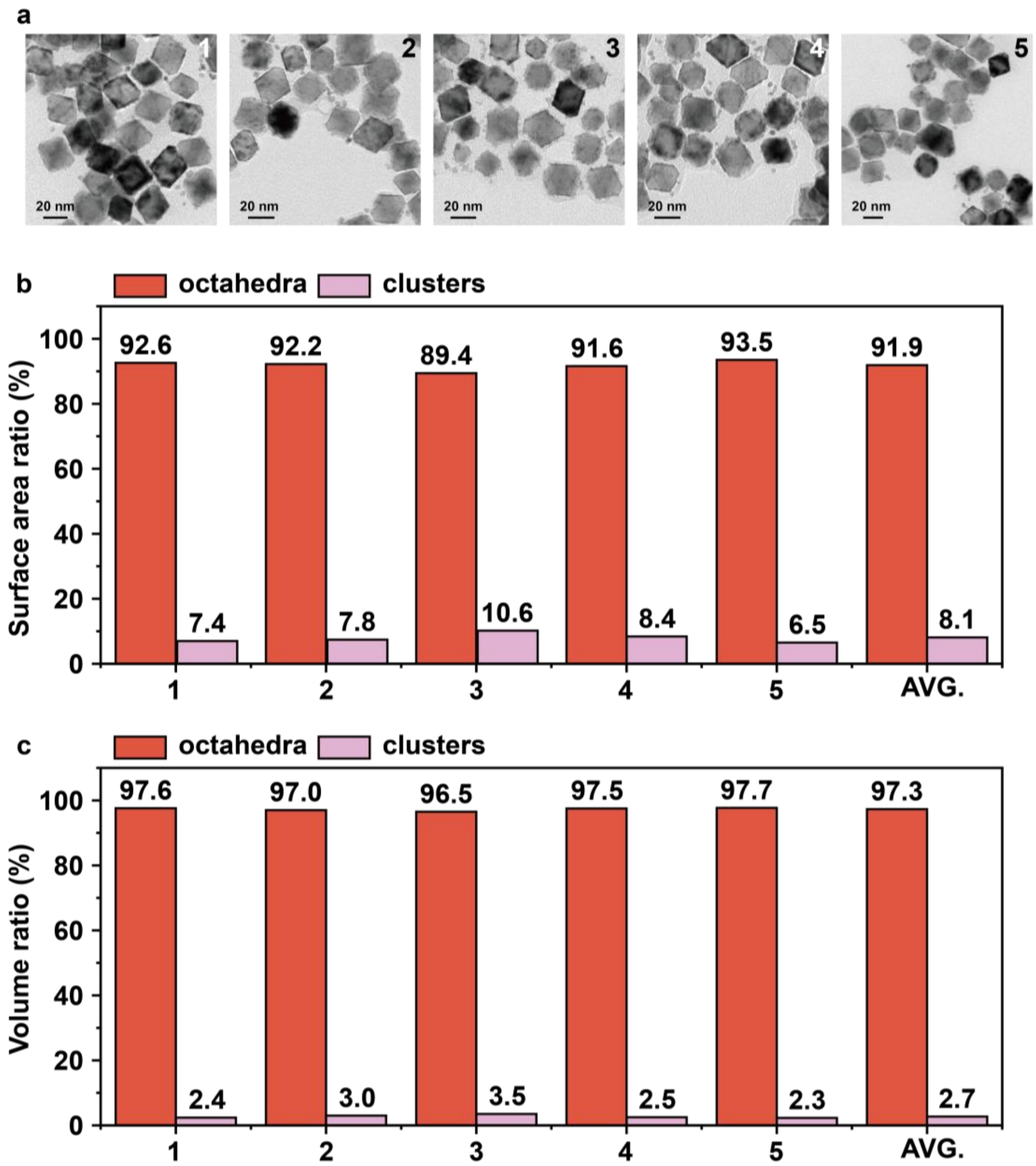

Figure S4. Quantitative comparison of the surface area and volume contributions of the representative $Pd@Pd_{0.2}Pt_{0.2}Ir_{0.2}Ru_{0.2}Rh_{0.2}$ core-shell nanooctahedra and a minor fraction of accompanying self-nucleated clusters. (a) Five representative TEM images used for statistical analysis. (b) Estimated surface area fractions of the core-shell nanooctahedra and self-nucleated clusters in each TEM image. The nanooctahedra account for an average of 91.9% of the total surface area, whereas the self-nucleated clusters contribute 8.1%. (c) Estimated volume fractions of the core-shell nanooctahedra and self-nucleated clusters, with average contributions of 97.3% and 2.7%, respectively. These results suggest that the self-nucleated clusters contribute only a relatively small fraction of the catalytic surface area and ensemble-averaged spectroscopic signals compared with the core-shell nanooctahedra.

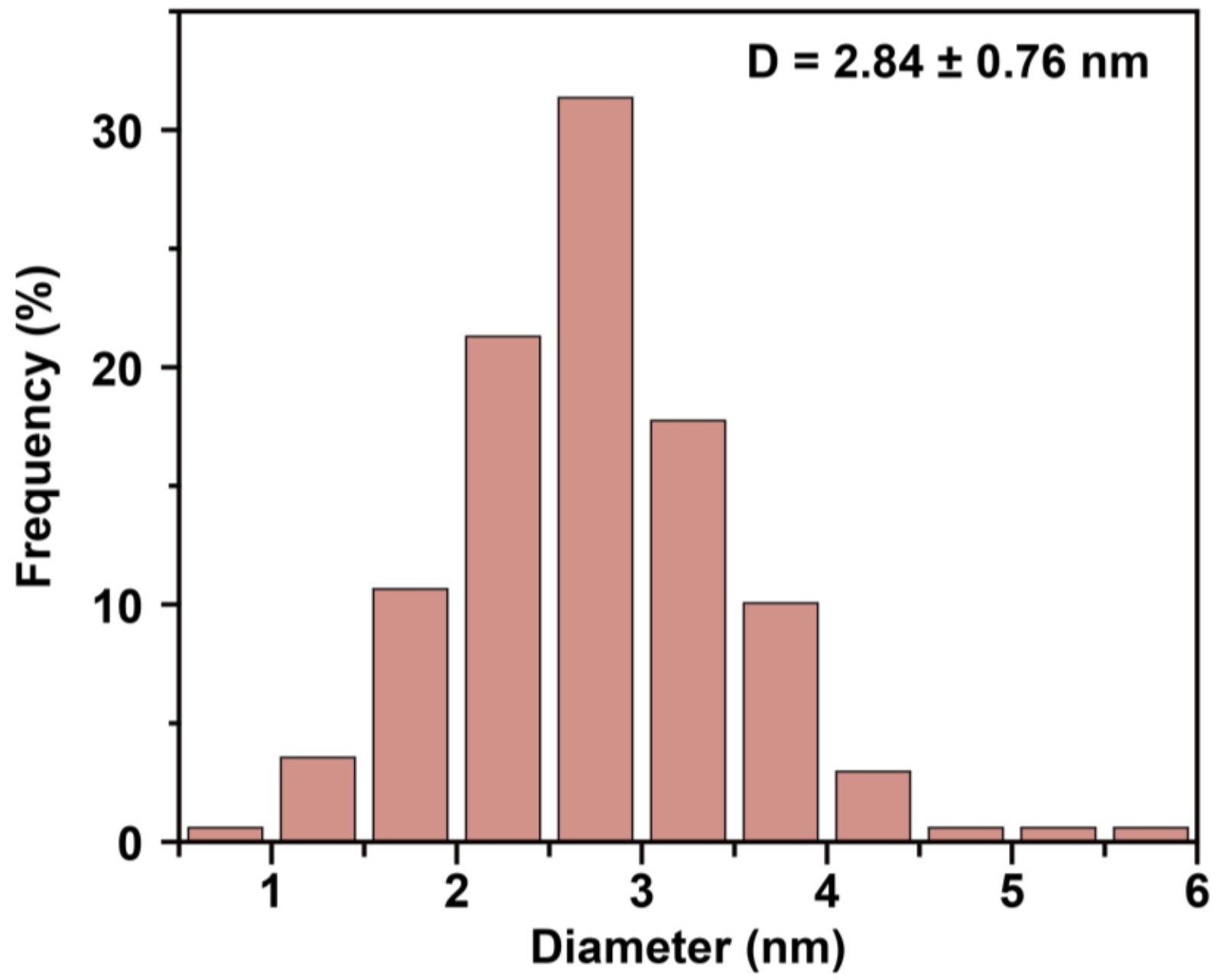

Figure S5. Diameter distribution of the self-nucleated clusters formed during the synthesis of Pd@$Pd_{0.2}Pt_{0.2}Ir_{0.2}Ru_{0.2}Rh_{0.2}$ core-shell nanooctahedra, determined from the TEM images in Figure S4a. The self-nucleated clusters exhibit an average diameter of 2.84 ± 0.76 nm.

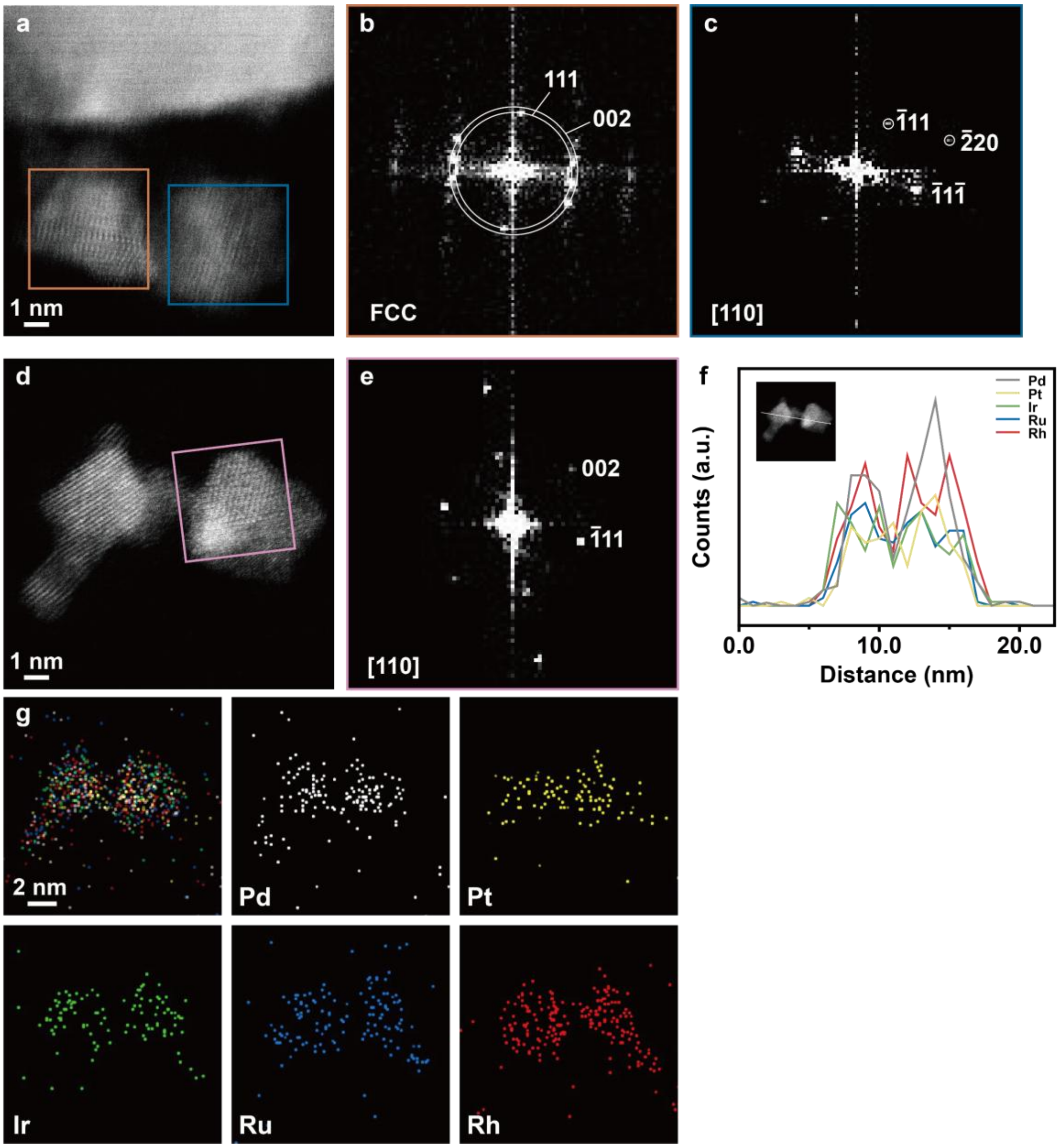

Figure S6. Structural and compositional characterization of self-nucleated clusters formed during the synthesis of $Pd@Pd_{0.2}Pt_{0.2}Ir_{0.2}Ru_{0.2}Rh_{0.2}$ core-shell nanooctahedra. (a) HAADF-STEM image of self-nucleated clusters located adjacent to a nanooctahedron, with the orange and blue boxes indicating the regions selected for FFT analysis. (b, c) Corresponding FFT patterns of the orange- and blue-boxed regions in (a), respectively. (d) HAADF-STEM image of isolated self-nucleated clusters, with the pink box indicating the region selected for FFT analysis. (e) Corresponding FFT pattern of the pink-boxed region in (d), viewed along the [110] zone axis. (f, g) EDS line-scan profiles and overlaid and individual elemental maps showing the distributions of Pd, Pt, Ir, Ru, and Rh within representative self-nucleated clusters. The line-scan profiles show a slightly stronger Pd contribution in some regions,

possibly reflecting the relatively faster reduction kinetics of the Pd precursor.[23] Overall, these self-nucleated clusters are predominantly FCC and contain all five constituent elements, indicating that they represent minor multimetallic byproducts rather than a separate single-element crystalline phase.

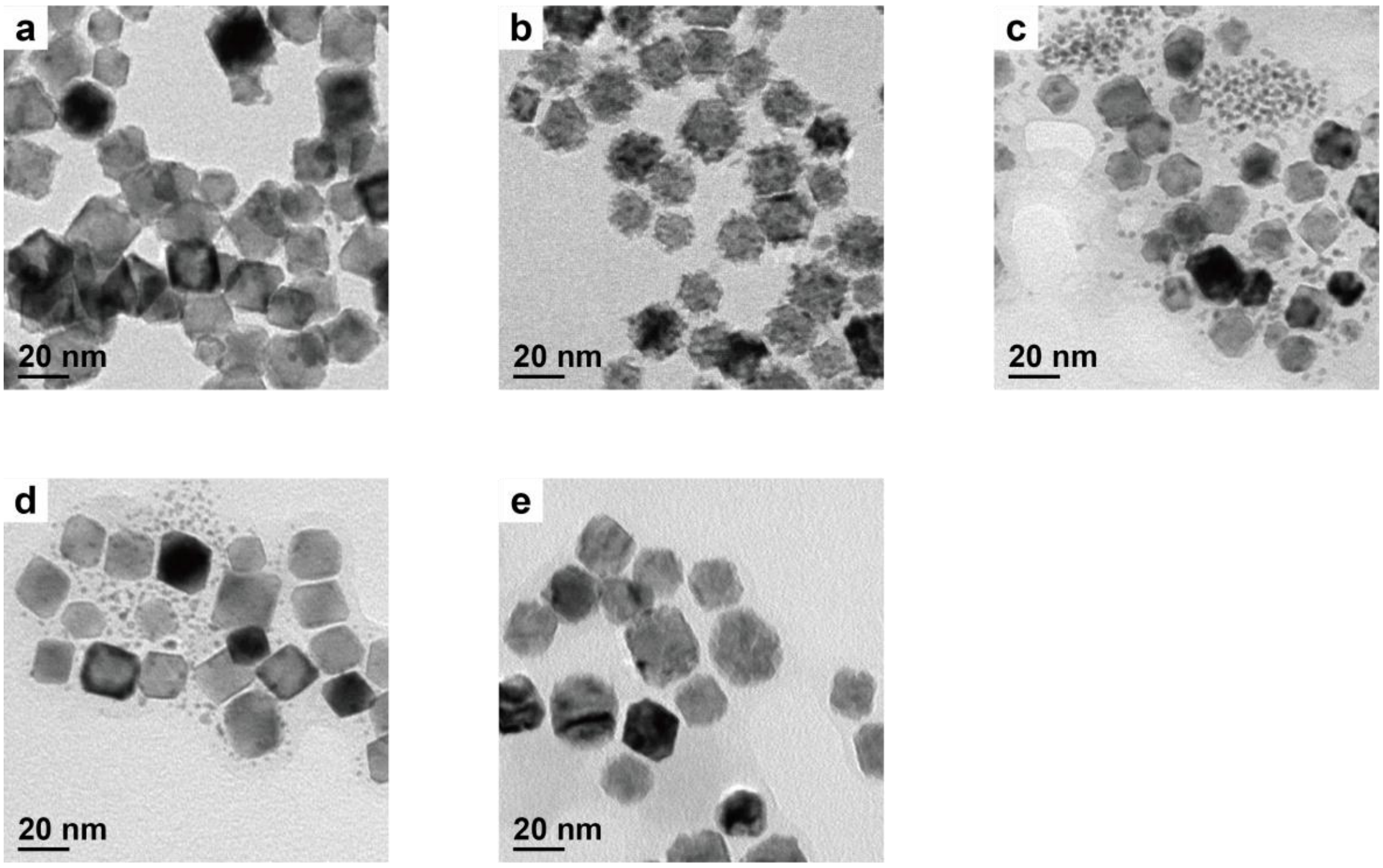

Figure S7. TEM images of simplified reference catalysts based on {111}-faceted nanooctahedra: (a) $Pd@Pt_{0.33}Ir_{0.33}Ru_{0.33}$, (b) $Pd@Ir_{0.5}Ru_{0.5}$, (c) $Pd@Pt_{0.5}Ru_{0.5}$, (d) Pd@Pt, and (e) Pd@Ru nanooctahedra.

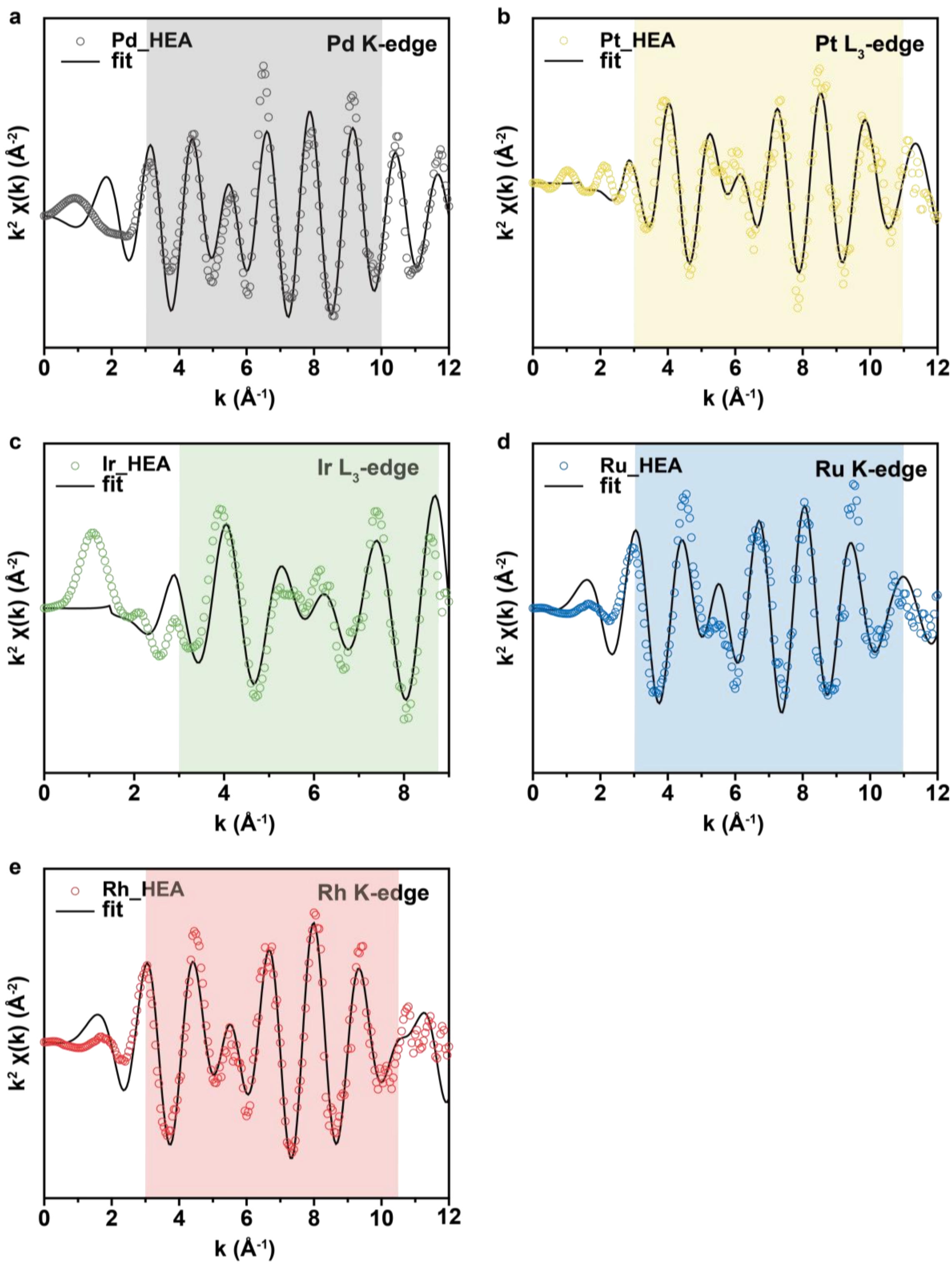

Figure S8. FT-EXAFS spectra (points) and corresponding fits (lines) of Pd@$Pd_{0.2}Pt_{0.2}Ir_{0.2}Ru_{0.2}Rh_{0.2}$ nanooctahedra in k-space. (a) Pd K-edge, (b) Pt L_3-edge, (c) Ir L_3-edge, (d) Ru K-edge, and (e) Rh K-edge. The fitting k ranges were 3–10 $Å^{-1}$ for Pd, 3–11.5 $Å^{-1}$ for Pt and Ru, 3–8.8 $Å^{-1}$ for Ir, and 3–11 $Å^{-1}$ for Rh.

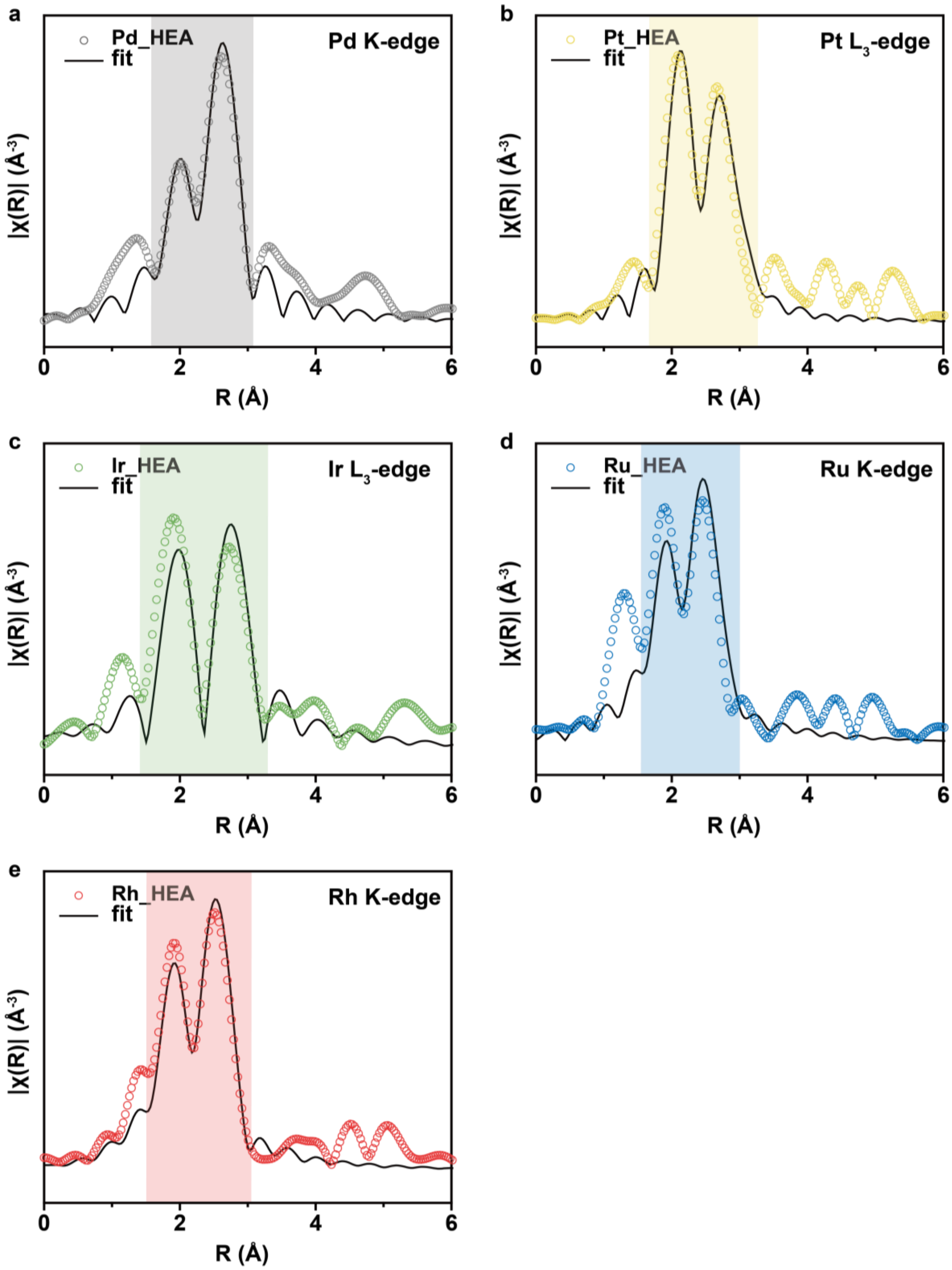

Figure S9. FT-EXAFS spectra (points) and corresponding fits (lines) of $Pd@Pd_{0.2}Pt_{0.2}Ir_{0.2}Ru_{0.2}Rh_{0.2}$ nanooctahedra in R-space. (a) Pd K-edge, (b) Pt L_3-edge, (c) Ir L_3-edge, (d) Ru K-edge, and (e) Rh K-edge. The fitting analyses were performed over a R range of 1.6–3.1 Å for Pd, 1.6–3.0 Å for Pt, 1.4–3.2 Å for Ir, and 1.5–3.0 Å for Ru and Rh.

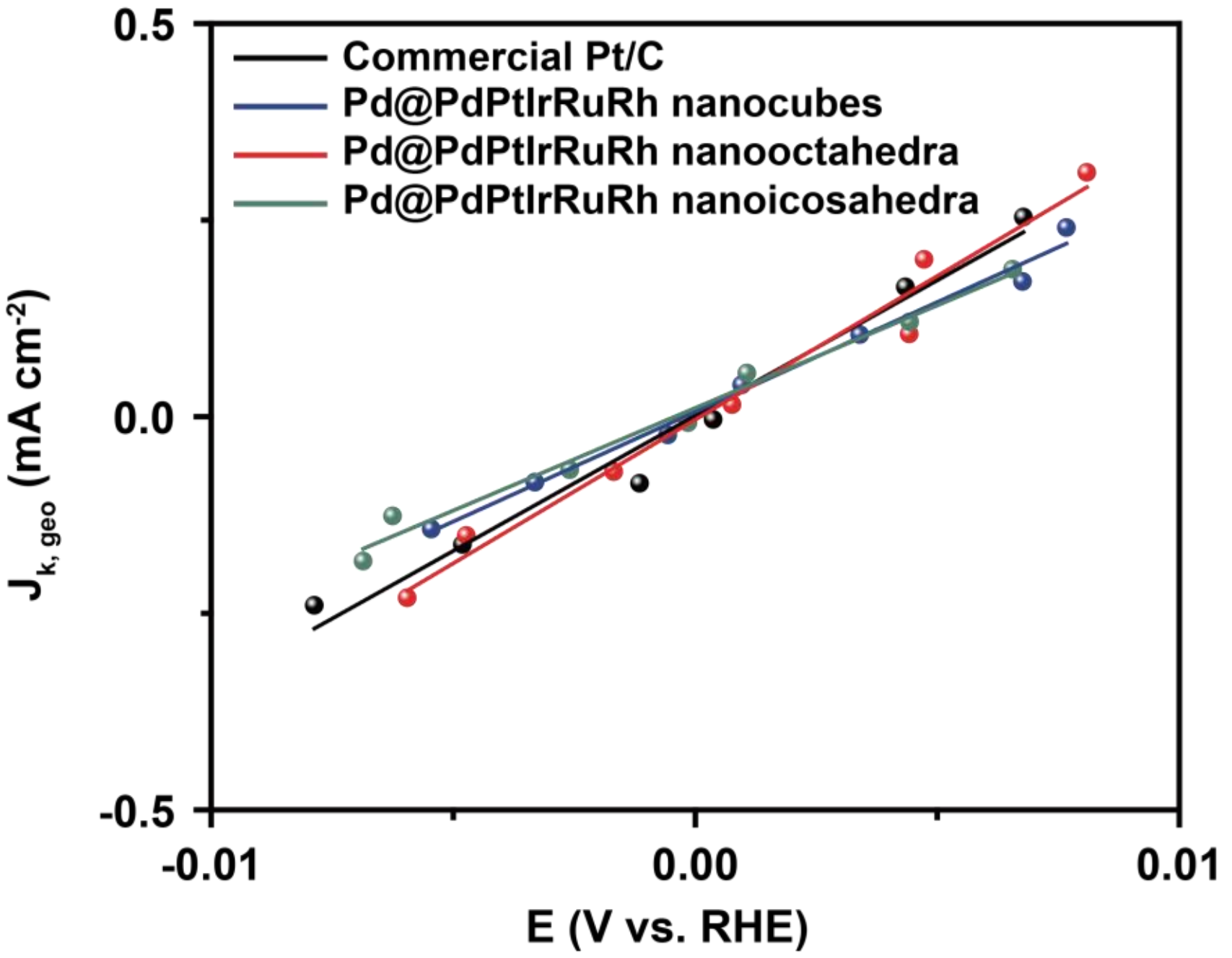

Figure S10. Micropolarization plots for the alkaline HOR in the low-overpotential region ($|\eta| \leq$ 10 mV). Solid lines represent linear fits to the data. The exchenge current densities (j_0) were calculated from the micropolarization region according to the procedure describe in the Materials and Methods section. The j_0 values of commercial Pt/C, $Pd@Pd_{0.2}Pt_{0.2}Ir_{0.2}Ru_{0.2}Rh_{0.2}$ nanocubes, nanooctahedra, and nanoicosahedra are 0.4418, 0.3583, 0.4693, and 0.3350 mA cm^{-2}, respectively.

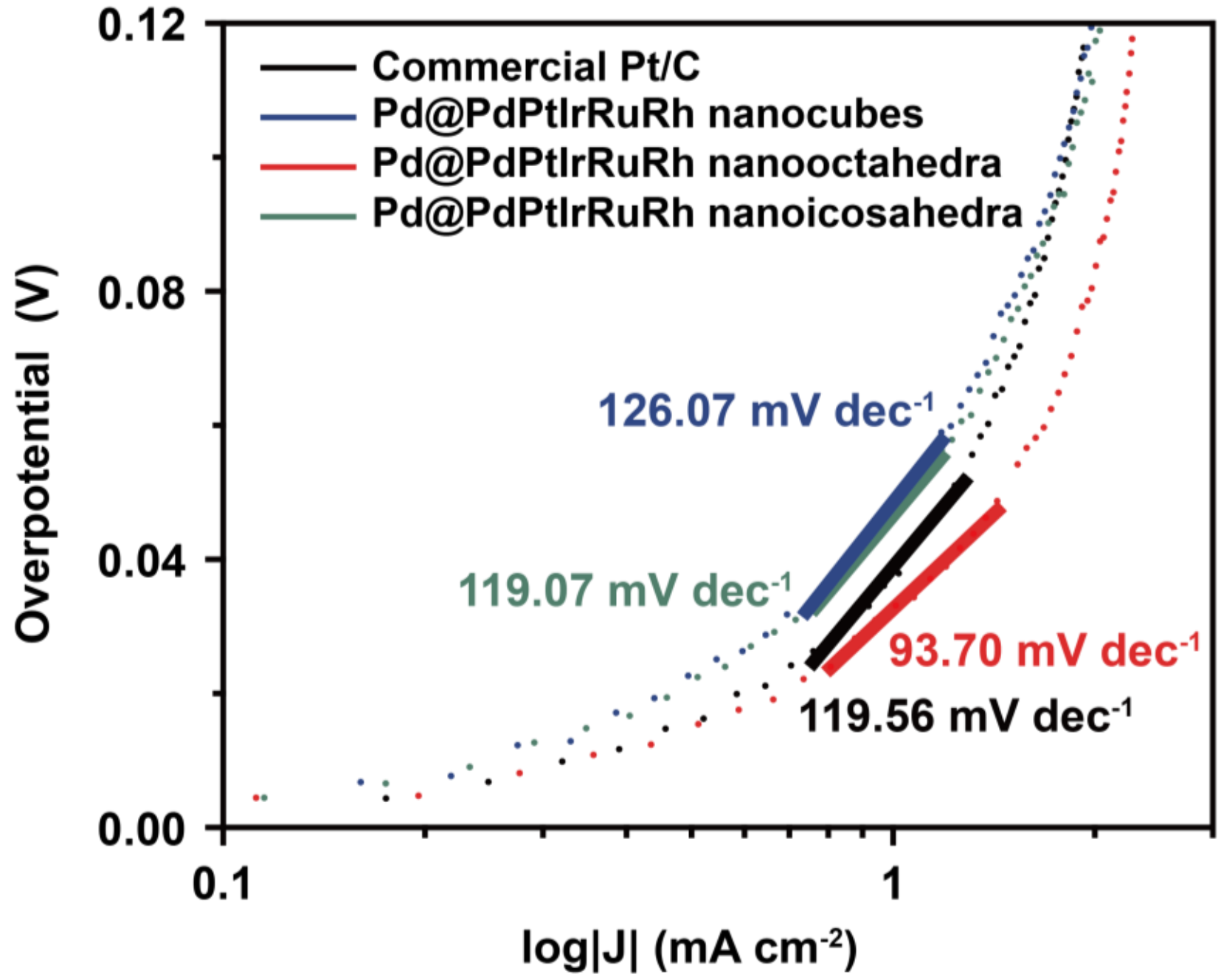

Figure S11. Tafel plots derived from the alkaline HOR polarization curves. The symbols represent the experimental data, and the solid lines indicate the linear fits used to determine the Tafel slopes.

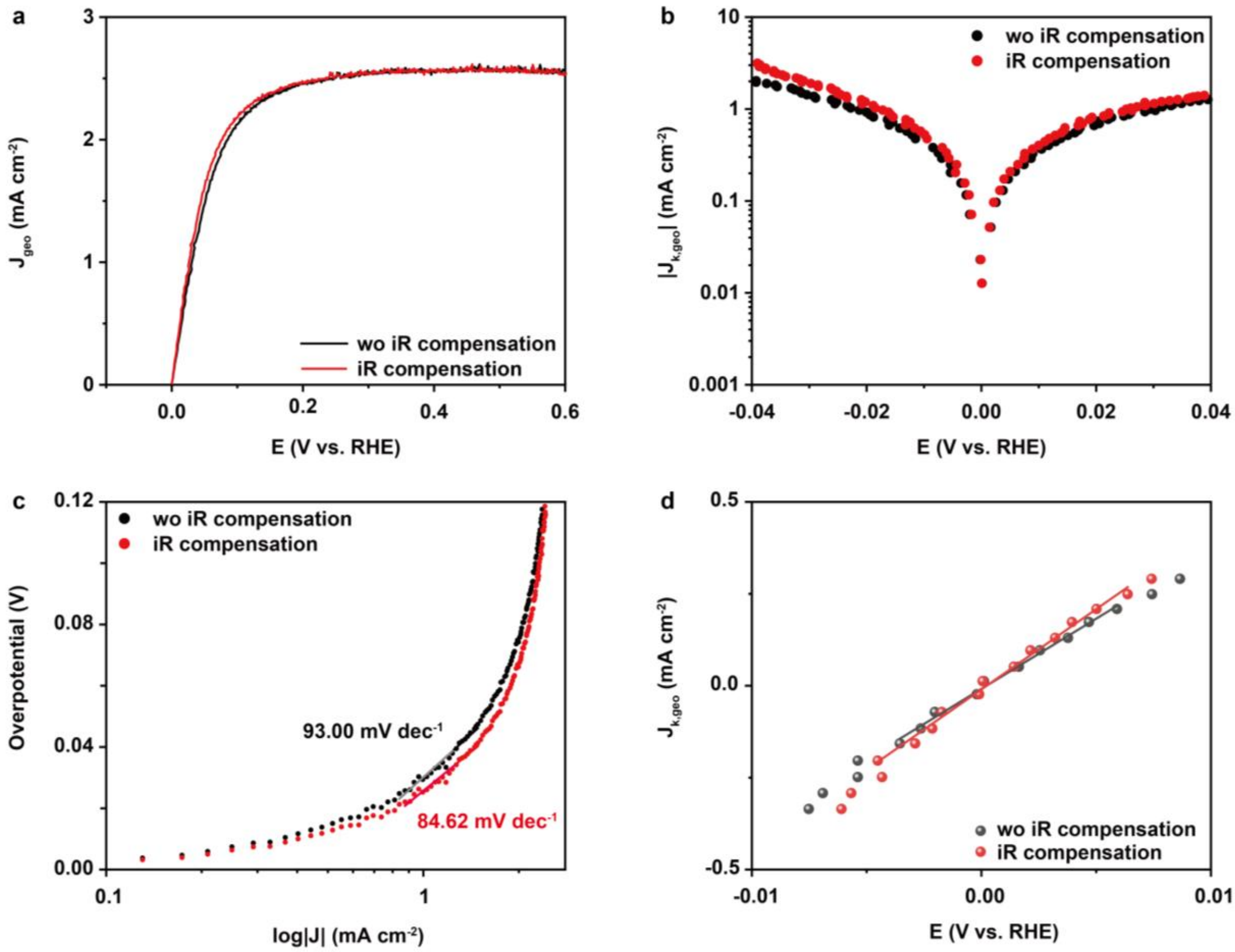

Figure S12. Influence of iR and mass-transport corrections on the HOR kinetic analysis of the {111}-enclosed Pd@$Pd_{0.2}Pt_{0.2}Ir_{0.2}Ru_{0.2}Rh_{0.2}$ core-shell nanooctahedra. (a) HOR polarization curves before and after 85% iR correction. (b) Tafel plots obtained from the HOR polarization curves before and after iR correction. (c) Tafel plots derived from the kinetic current densities (J_k) after mass-transport correction, showing Tafel slopes of 93.00 and 84.62 mV dec^{-1} before and after iR correction, respectively. (d) Micropolarization plots derived from the mass-transport-corrected J_k before and after iR correction. Linear fitting of the near-equilibrium region yielded exchange current densities (j_0) of 0.4903 and 0.5605 mA cm^{-2} before and after iR correction, respectively. Because no real-time iR compensation was applied, post-measurement iR correction was performed using the uncompensated resistance (R_u) obtained from electrochemical impedance spectroscopy (EIS). To avoid overcompensation, 85% of R_u was used. The current density was then converted to kinetic current density using the Koutecky-Levich relationship, with the diffusion-limited current density obtained from the corresponding HOR polarization curve recorded at 1600 rpm.

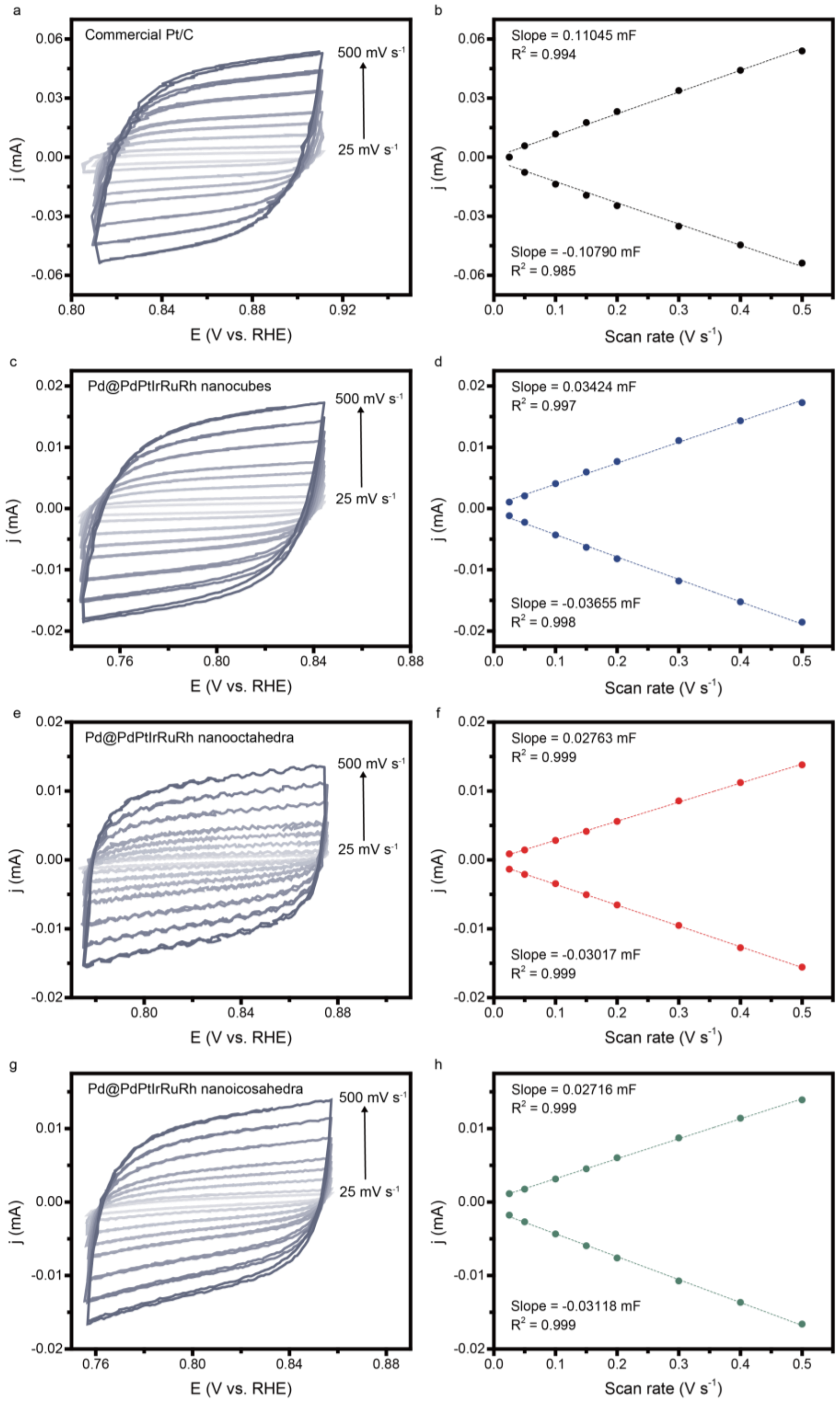

Figure S13. CV curves recorded at various scan rates for estimating ECSA using C_{dl} as a surface-area proxy. CV curves and the corresponding linear fits of current versus scan rate are shown for (a, b) commercial Pt/C, (c, d) $Pd@Pd_{0.2}Pt_{0.2}Ir_{0.2}Ru_{0.2}Rh_{0.2}$ nanocubes, (e, f) $Pd@Pd_{0.2}Pt_{0.2}Ir_{0.2}Ru_{0.2}Rh_{0.2}$ nanooctahedra, and (g, h) $Pd@Pd_{0.2}Pt_{0.2}Ir_{0.2}Ru_{0.2}Rh_{0.2}$ nanoicosahedra. The calculated C_{dl} values are 109.175, 35.395, 28.900, and 29.170 μF, respectively.

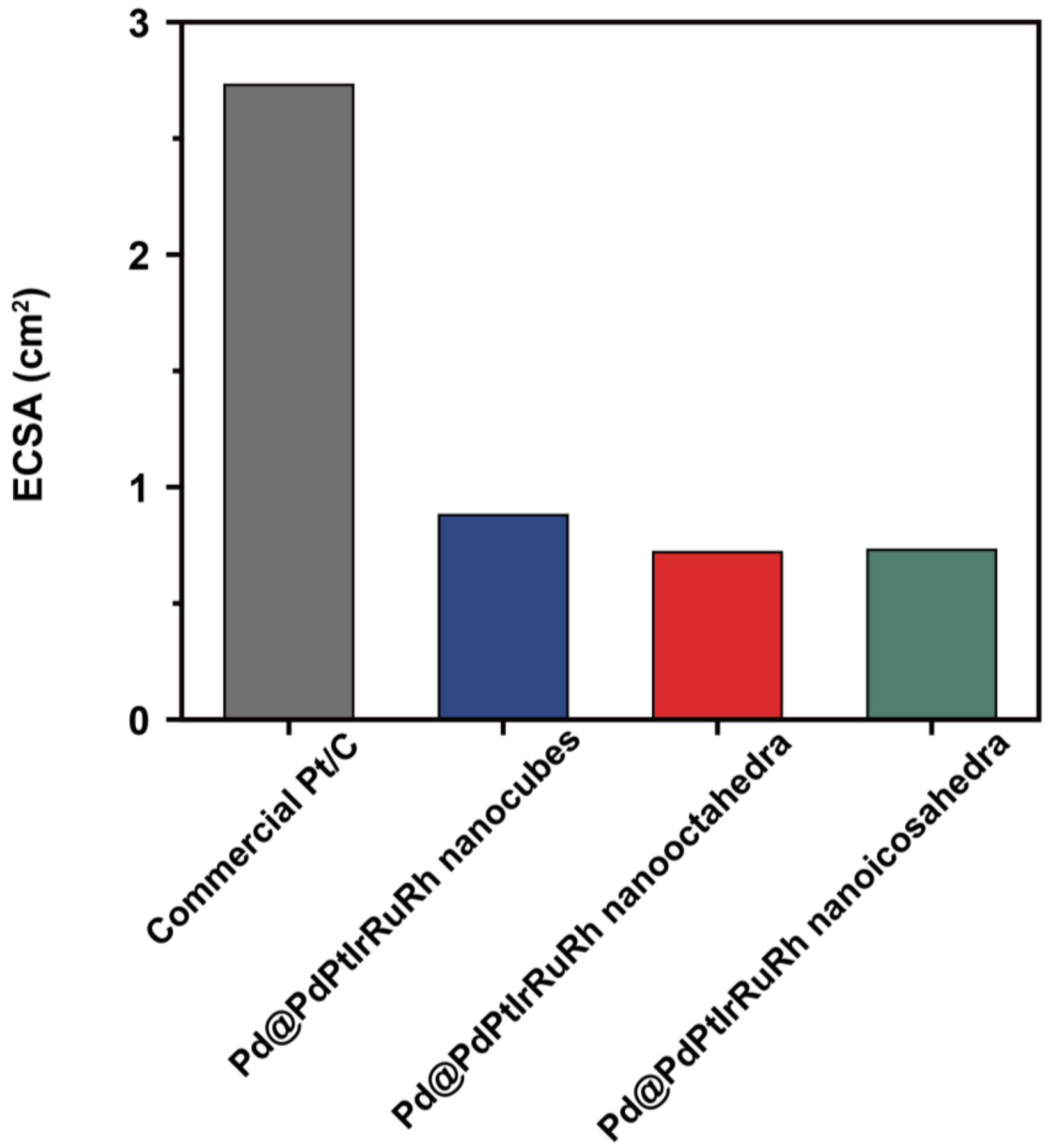

Figure S14. Comparison of ECSA values of commercial Pt/C and Pd@$Pd_{0.2}Pt_{0.2}Ir_{0.2}Ru_{0.2}Rh_{0.2}$ nanocrystals.

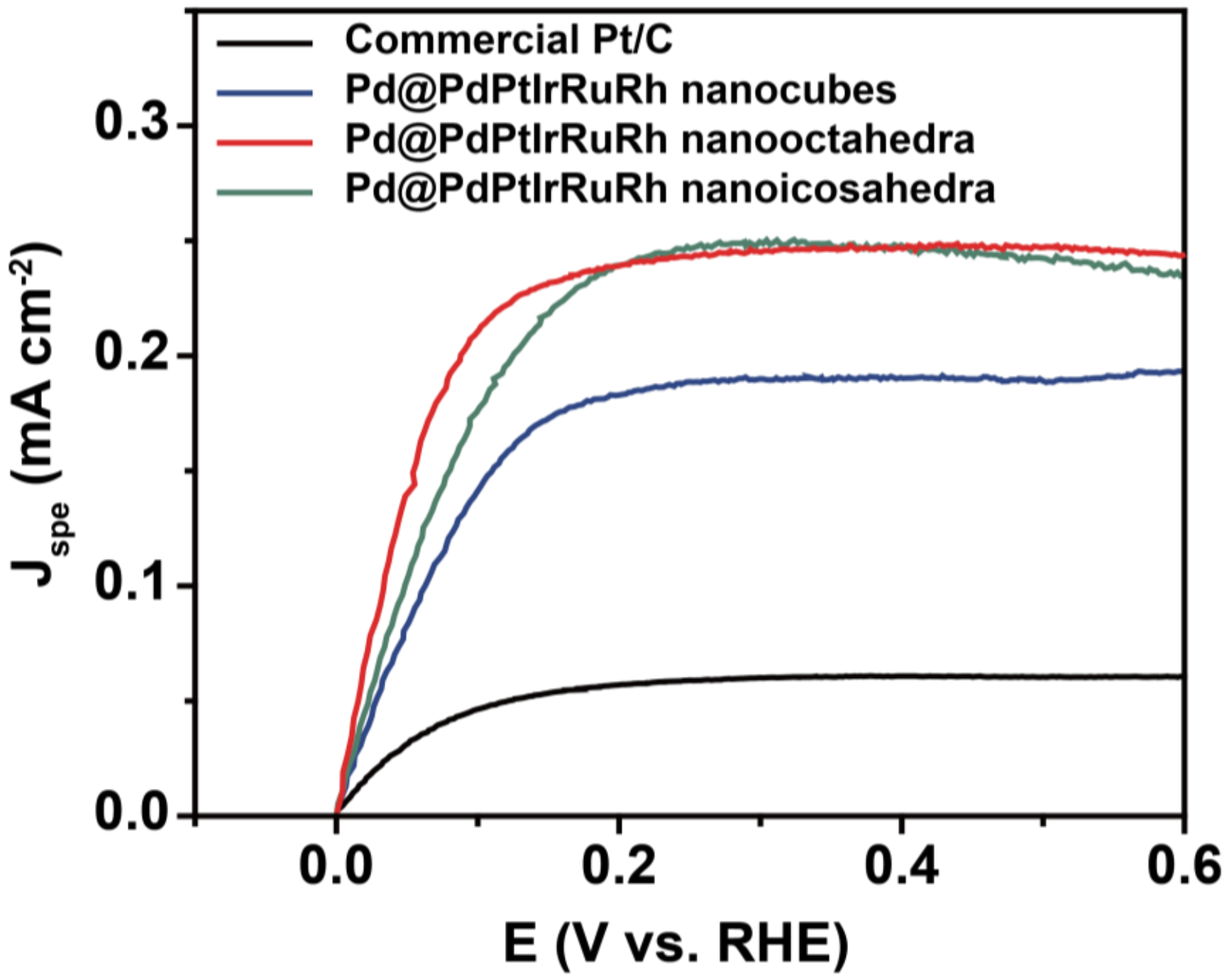

Figure S15. LSV curves with current normalized to ECSA for commercial Pt/C and $Pd@Pd_{0.2}Pt_{0.2}Ir_{0.2}Ru_{0.2}Rh_{0.2}$ nanocrystals.

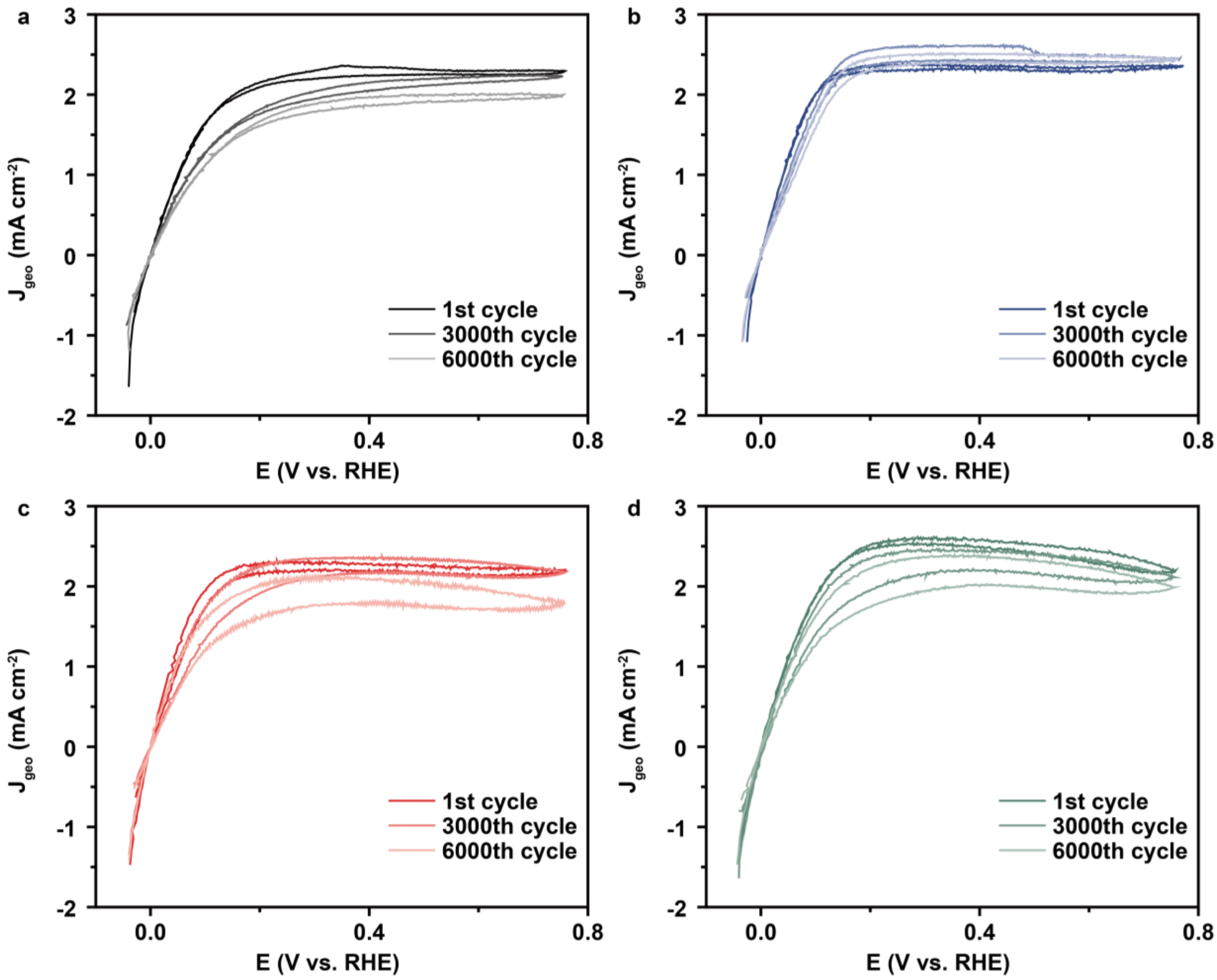

Figure S16. Alkaline HOR durability evaluated using CV curves for (a) Commercial Pt/C, (b) $Pd@Pd_{0.2}Pt_{0.2}Ir_{0.2}Ru_{0.2}Rh_{0.2}$ nanocubes, (c) $Pd@Pd_{0.2}Pt_{0.2}Ir_{0.2}Ru_{0.2}Rh_{0.2}$ nanooctahedra, and (d) $Pd@Pd_{0.2}Pt_{0.2}Ir_{0.2}Ru_{0.2}Rh_{0.2}$ nanoicosahedra.

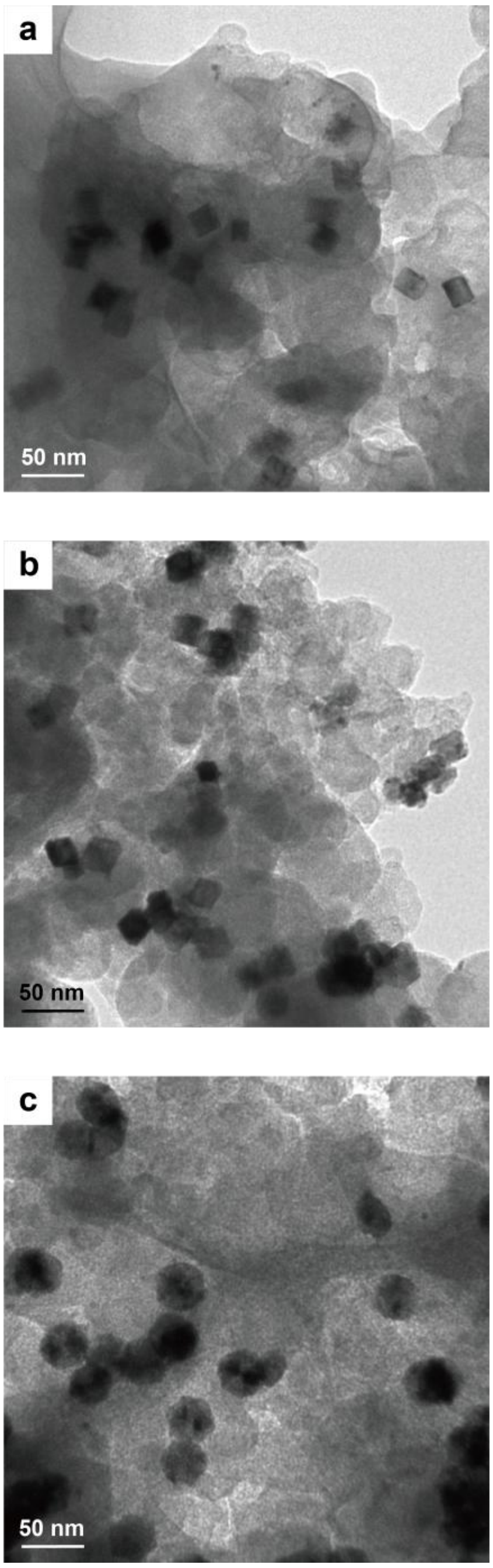

Figure S17. TEM images of catalysts acquired after the alkaline HOR durability test. (a) $Pd@Pd_{0.2}Pt_{0.2}Ir_{0.2}Ru_{0.2}Rh_{0.2}$ nanocubes, (b) $Pd@Pd_{0.2}Pt_{0.2}Ir_{0.2}Ru_{0.2}Rh_{0.2}$ nanooctahedra, and (c) $Pd@Pd_{0.2}Pt_{0.2}Ir_{0.2}Ru_{0.2}Rh_{0.2}$ nanoicosahedra.

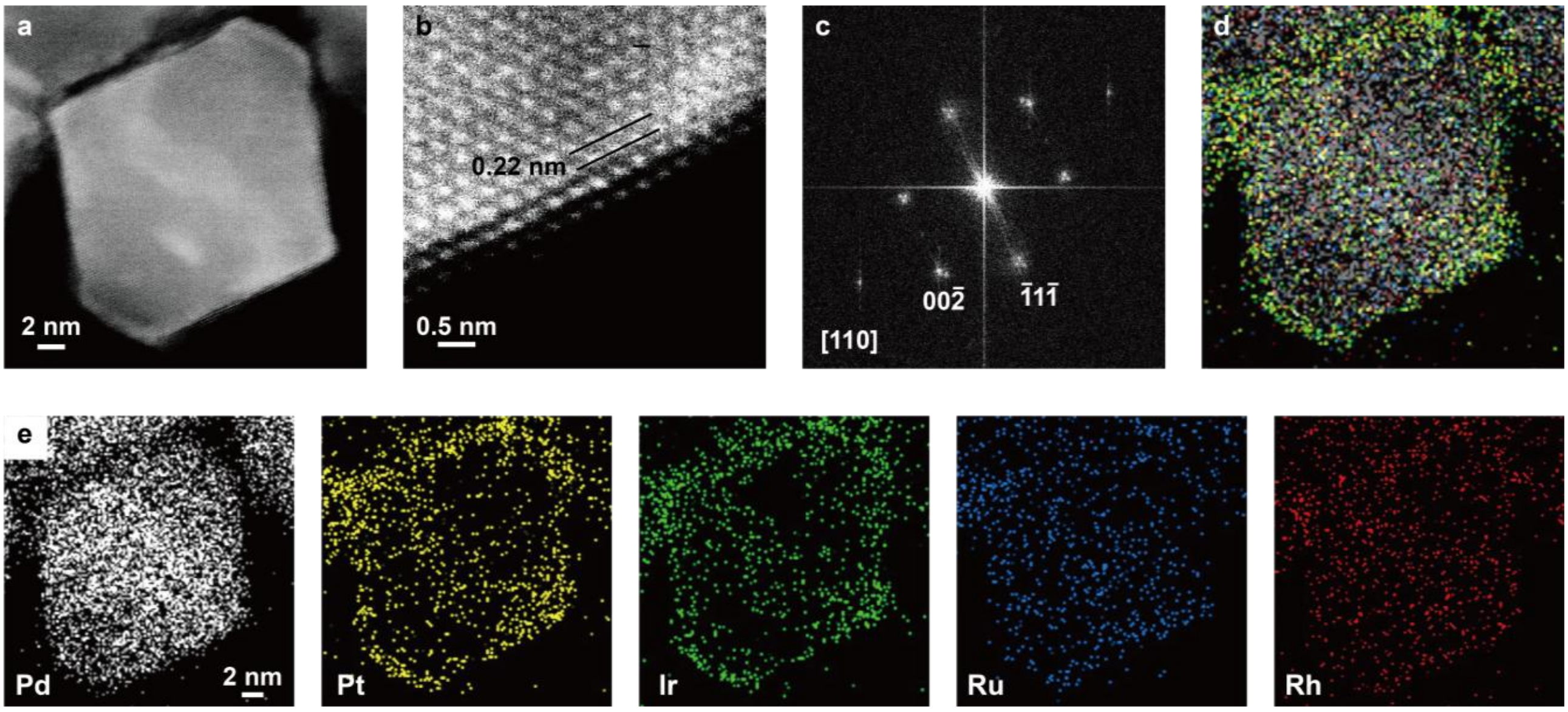

Figure S18. Structural and compositional characterization of the representative $Pd@Pd_{0.2}Pt_{0.2}Ir_{0.2}Ru_{0.2}Rh_{0.2}$ core-shell nanooctahedra as catalysts acquired after 6000 cycles of the alkaline HOR durability test. (a) Low-magnification and (b) atomic-resolution HAADF-STEM images, (c) corresponding FFT pattern, and (d, e) EDS elemental maps showing (d) the overlaid distribution of all constituent elements and (e) the individual distributions of Pd, Pt, Ir, Ru, and Rh.

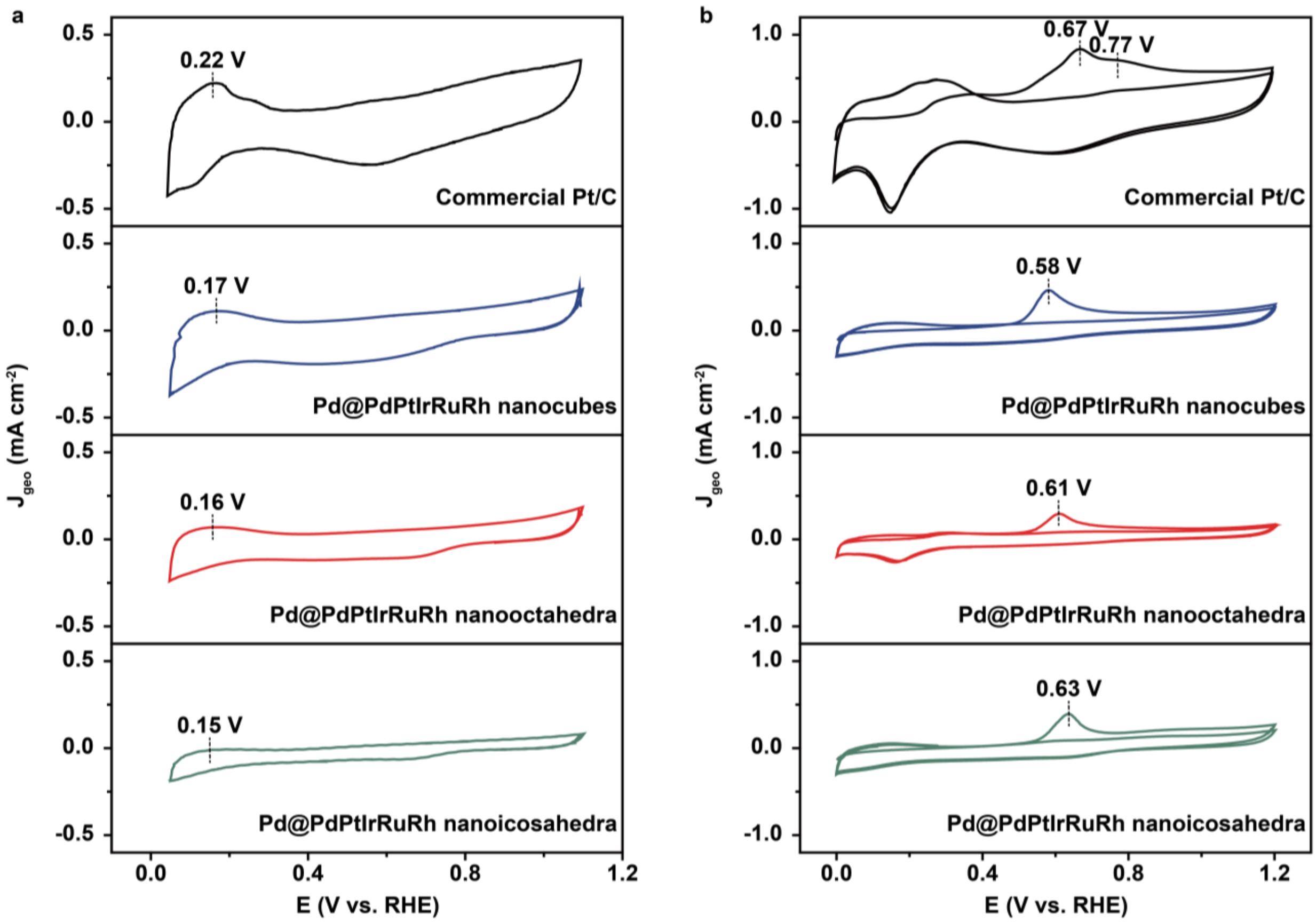

Figure S19. Probing hydrogen binding and hydroxyl adsorption characteristics. (a) HUPD CV curves and (b) CO stripping CV curves of commercial Pt/C, $Pd@Pd_{0.2}Pt_{0.2}Ir_{0.2}Ru_{0.2}Rh_{0.2}$ nanocubes, $Pd@Pd_{0.2}Pt_{0.2}Ir_{0.2}Ru_{0.2}Rh_{0.2}$ nanooctahedra, and $Pd@Pd_{0.2}Pt_{0.2}Ir_{0.2}Ru_{0.2}Rh_{0.2}$ nanoicosahedra.

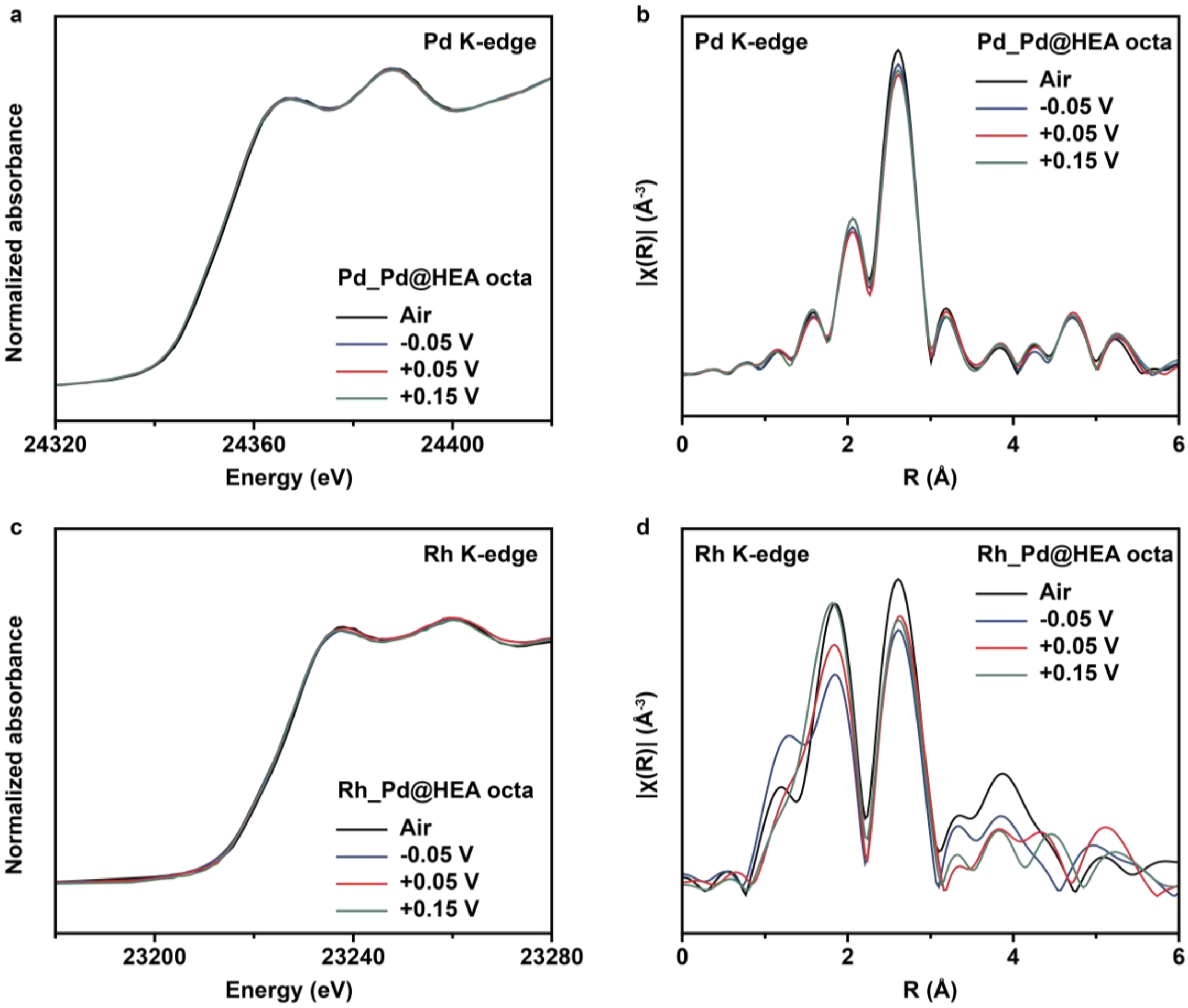

Figure S20. *In situ* XANES and FT-EXAFS spectra of Pd@$Pd_{0.2}Pt_{0.2}Ir_{0.2}Ru_{0.2}Rh_{0.2}$ nanooctahedra at (a, b) Pd K-edge and (c, d) Rh K-edge. Spectra were acquired in air and at applied potentials of –0.05, 0.05, and 0.15 V_{RHE} under alkaline HOR-relevant conditions.

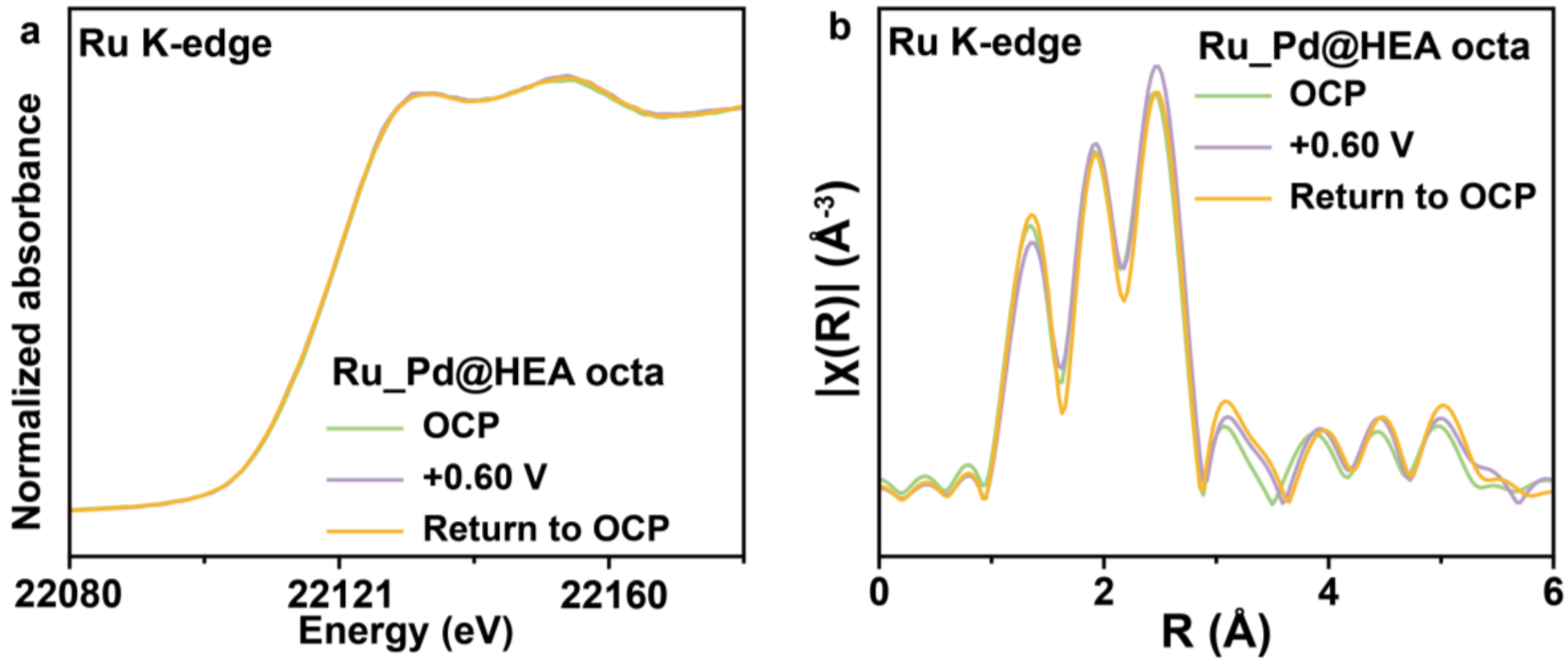

Figure S21. Reversibility test of the Ru local coordination environment for the $Pd@Pd_{0.2}Pt_{0.2}Ir_{0.2}Ru_{0.2}Rh_{0.2}$ core-shell nanooctahedra in 0.1 M KOH. (a) *In situ* Ru K-edge XANES spectra and (b) Fourier-transformed k^3-weighted EXAFS spectra collected at OCP, +0.60 V_{RHE}, and after returning to OCP. The XANES spectra show only minor changes throughout the potential cycle, whereas the ~1.5 Å FT-EXAFS feature exhibits a reversible potential-dependent variation. After returning to OCP, the ~1.5 Å feature largely recovers toward its initial value, while the higher-shell Ru-M features remain essentially unchanged, indicating largely reversible changes in the local Ru coordination environment without pronounced irreversible rearrangement of the Ru-M framework.

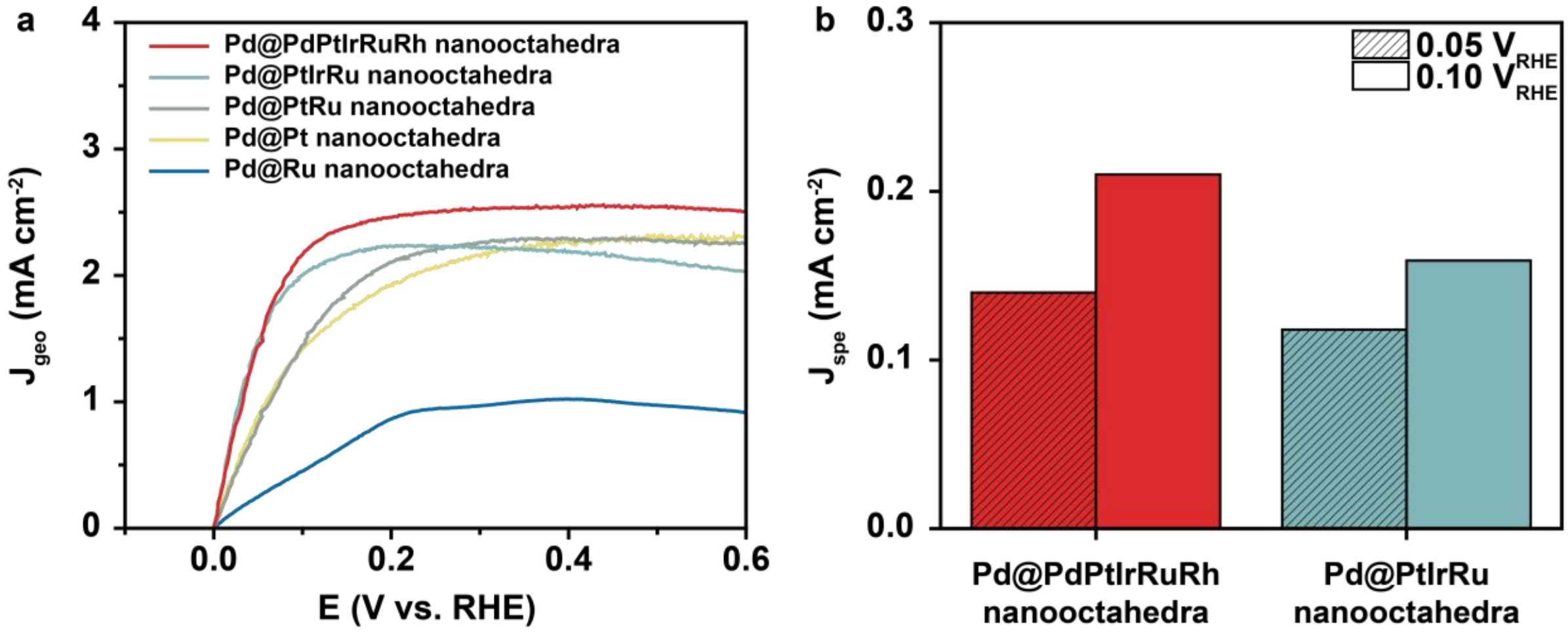

Figure S22. (a) Alkaline HOR polarization curves of $Pd@Pd_{0.2}Pt_{0.2}Ir_{0.2}Ru_{0.2}Rh_{0.2}$, $Pd@Pt_{0.33}Ir_{0.33}Ru_{0.33}$, $Pd@Pt_{0.5}Ru_{0.5}$, Pd@Pt, and Pd@Ru nanooctahedra. (b) Comparison of ECSA-normalized specific activities for $Pd@Pd_{0.2}Pt_{0.2}Ir_{0.2}Ru_{0.2}Rh_{0.2}$ and $Pd@Pt_{0.33}Ir_{0.33}Ru_{0.33}$ nanooctahedra at 0.05 and 0.10 V_{RHE}.

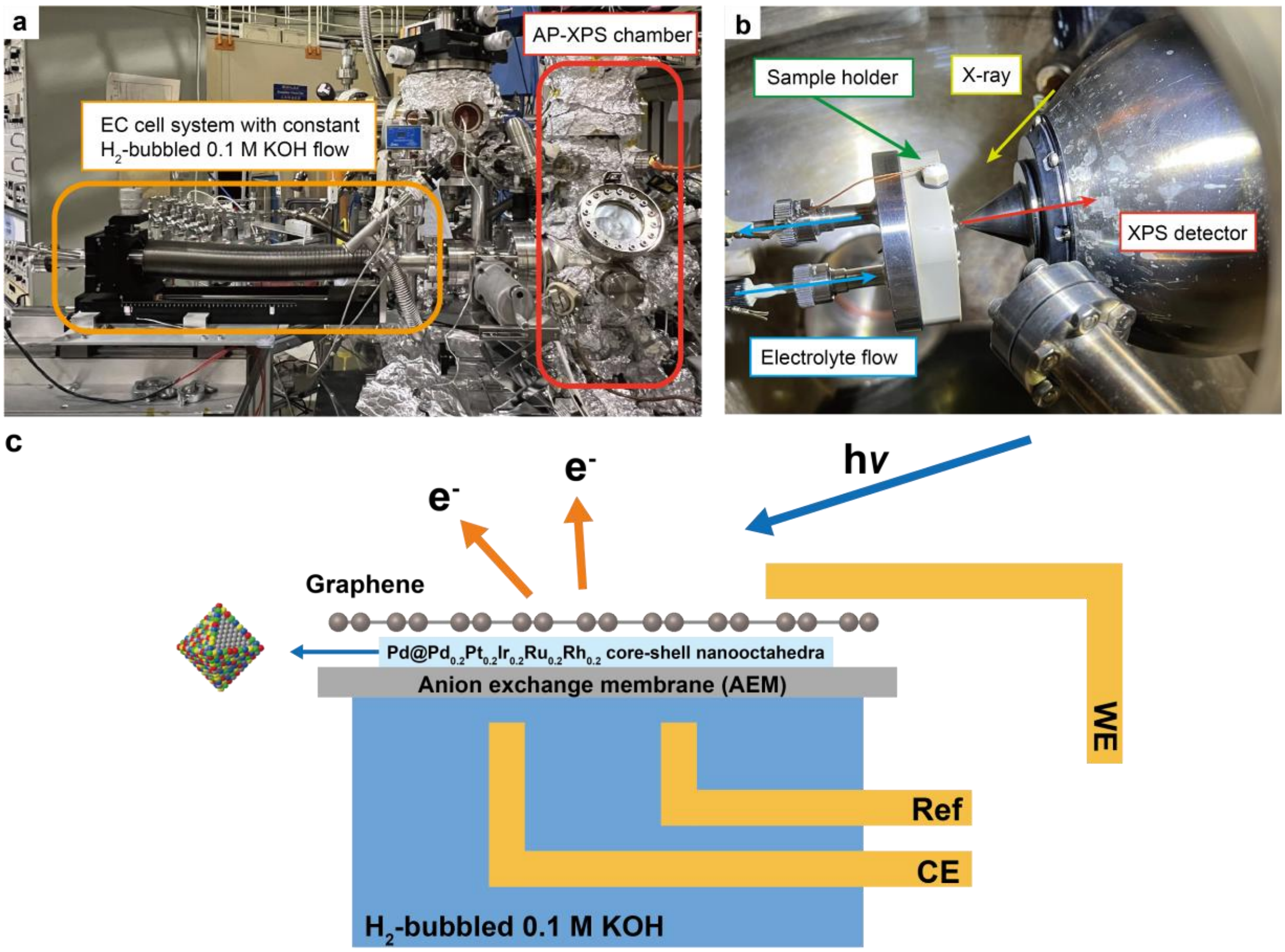

Figure S23. *In situ* synchrotron AP-XPS setup for probing surface chemical states of $Pd@Pd_{0.2}Pt_{0.2}Ir_{0.2}Ru_{0.2}Rh_{0.2}$ core-shell nanooctahedra during alkaline HOR. (a) Photograph of the AP-XPS system integrated with an electrochemical flow cell, in which H_2-saturated 0.1 M KOH electrolyte was continuously circulated to provide an alkaline HOR-relevant environment. (b) Enlarged view of the AP-XPS measurement configuration, showing the sample holder, electrolyte flow path, incident X-ray beam, and XPS detector geometry inside the AP-XPS chamber. (c) Schematic illustration of the electrochemical AP-XPS cell. The catalyst-coated working electrode was supported on an anion exchange membrane (AEM), while H_2-saturated 0.1 M KOH electrolyte was continuously supplied from the backside of the membrane. H_2O and OH^- are transported through the hydrated ion-conducting channels of the AEM, while dissolved H_2 can permeate through the hydrated membrane and reach the catalyst surface. The working, reference, and counter electrodes were electrochemically connected to enable potential control during AP-XPS measurements, allowing potential-dependent analysis of surface chemical states under HOR-relevant conditions.

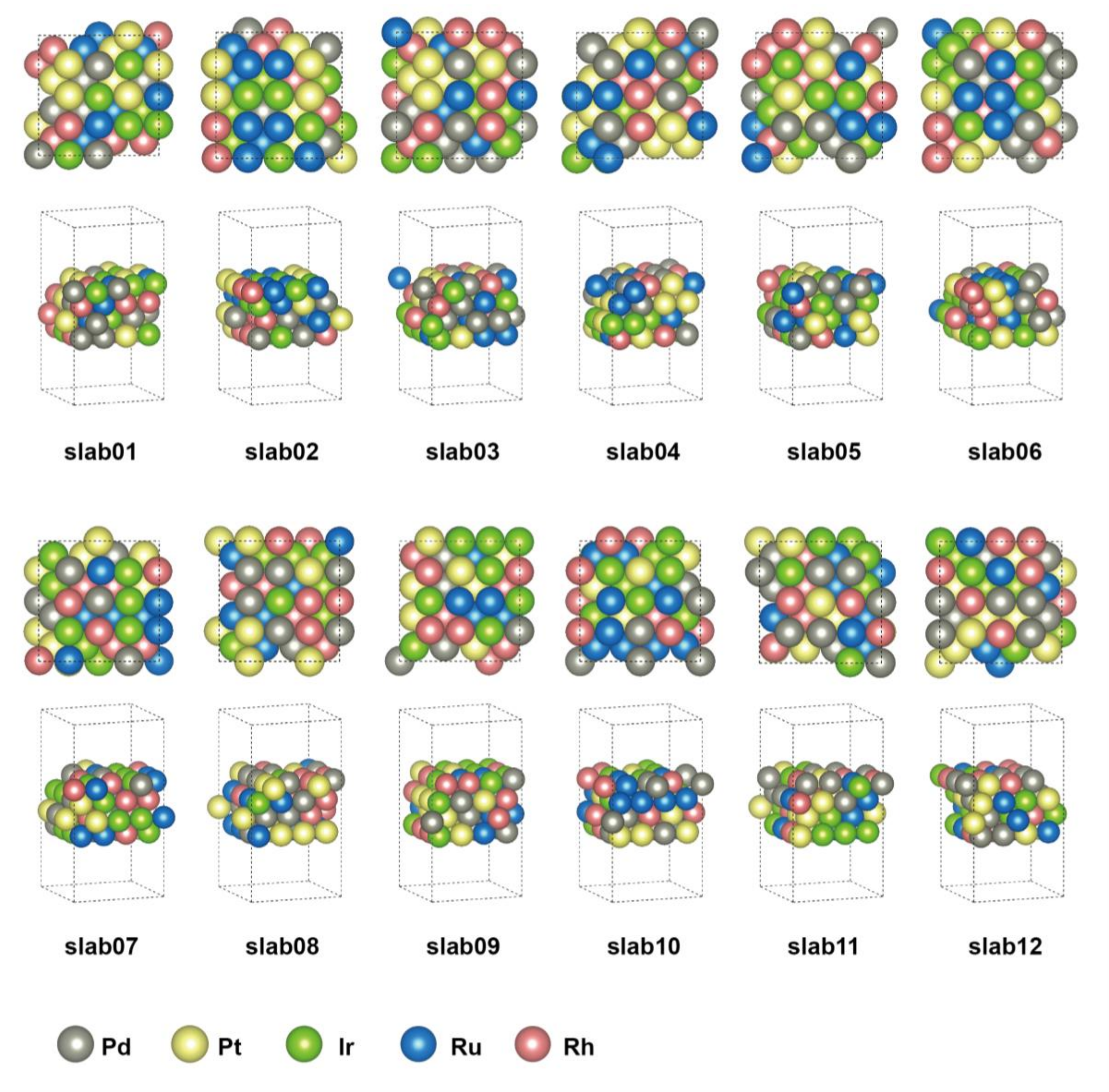

Figure S24. Structural configurations of the $Pd_{0.2}Pt_{0.2}Ir_{0.2}Ru_{0.2}Rh_{0.2}$ HEA {100} slab models after geometry optimization. The slabs were generated using the Atomic Simulation Environment (ASE) by randomly distributing Pd, Pt, Ir, Ru, and Rh atoms over the lattice sites while maintaining the prescribed alloy composition. Top and perspective views are shown for each independently randomized configuration. Different sphere colors represent Pd, Pt, Ir, Ru, and Rh. These models were used to sample diverse local atomic environments and adsorption-site ensembles for H^* and OH^* adsorption.

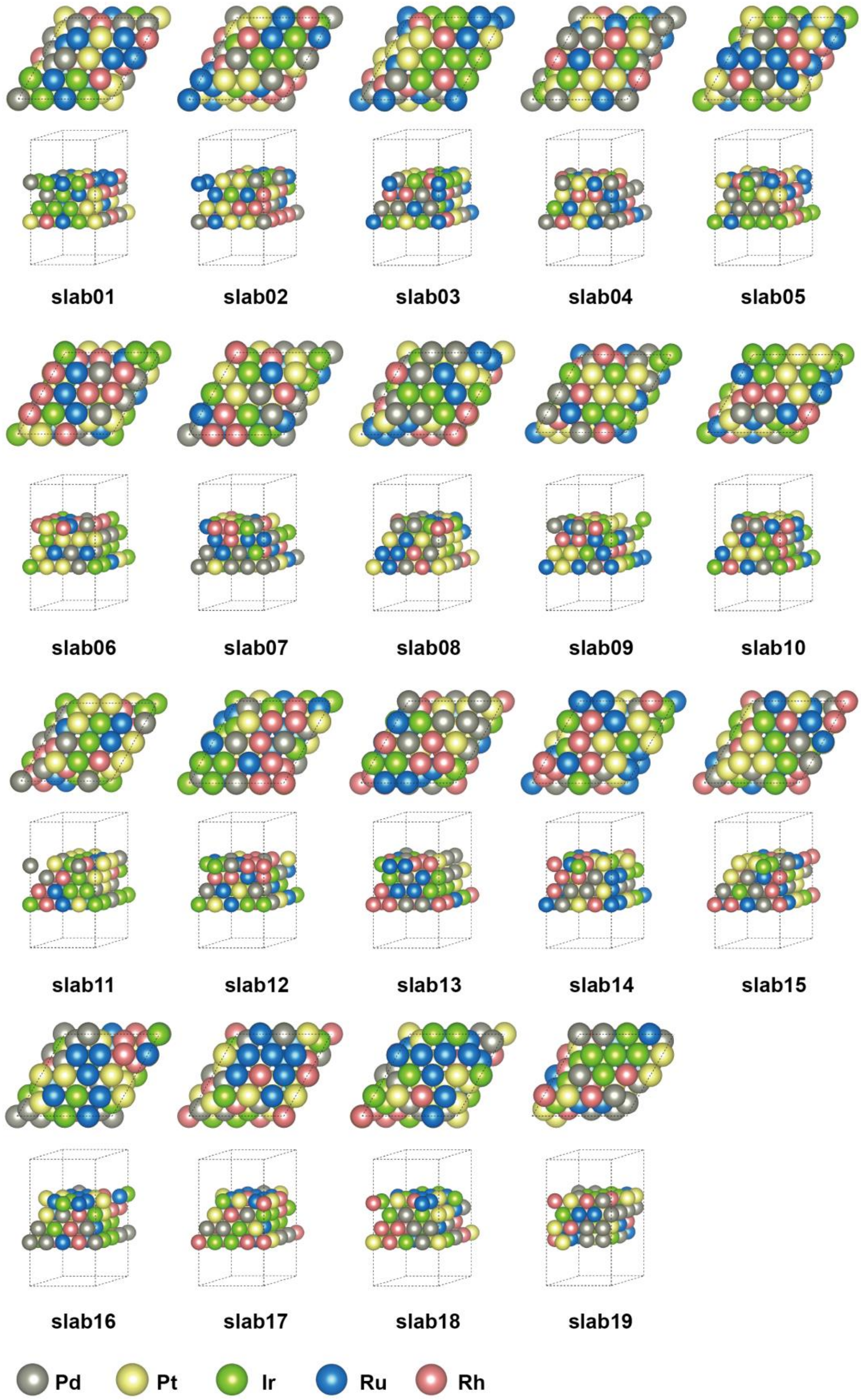

Figure S25. Structural configurations of the $Pd_{0.2}Pt_{0.2}Ir_{0.2}Ru_{0.2}Rh_{0.2}$ HEA {111} slab models after geometry optimization. The slabs were generated using the ASE by randomly distributing Pd, Pt, Ir,

Ru, and Rh atoms over the lattice sites while maintaining the prescribed alloy composition. Top and perspective views are shown for each independently randomized configuration. Different sphere colors represent Pd, Pt, Ir, Ru, and Rh. These models were used to sample diverse local atomic environments and adsorption-site ensembles for H^* and OH^* adsorption.

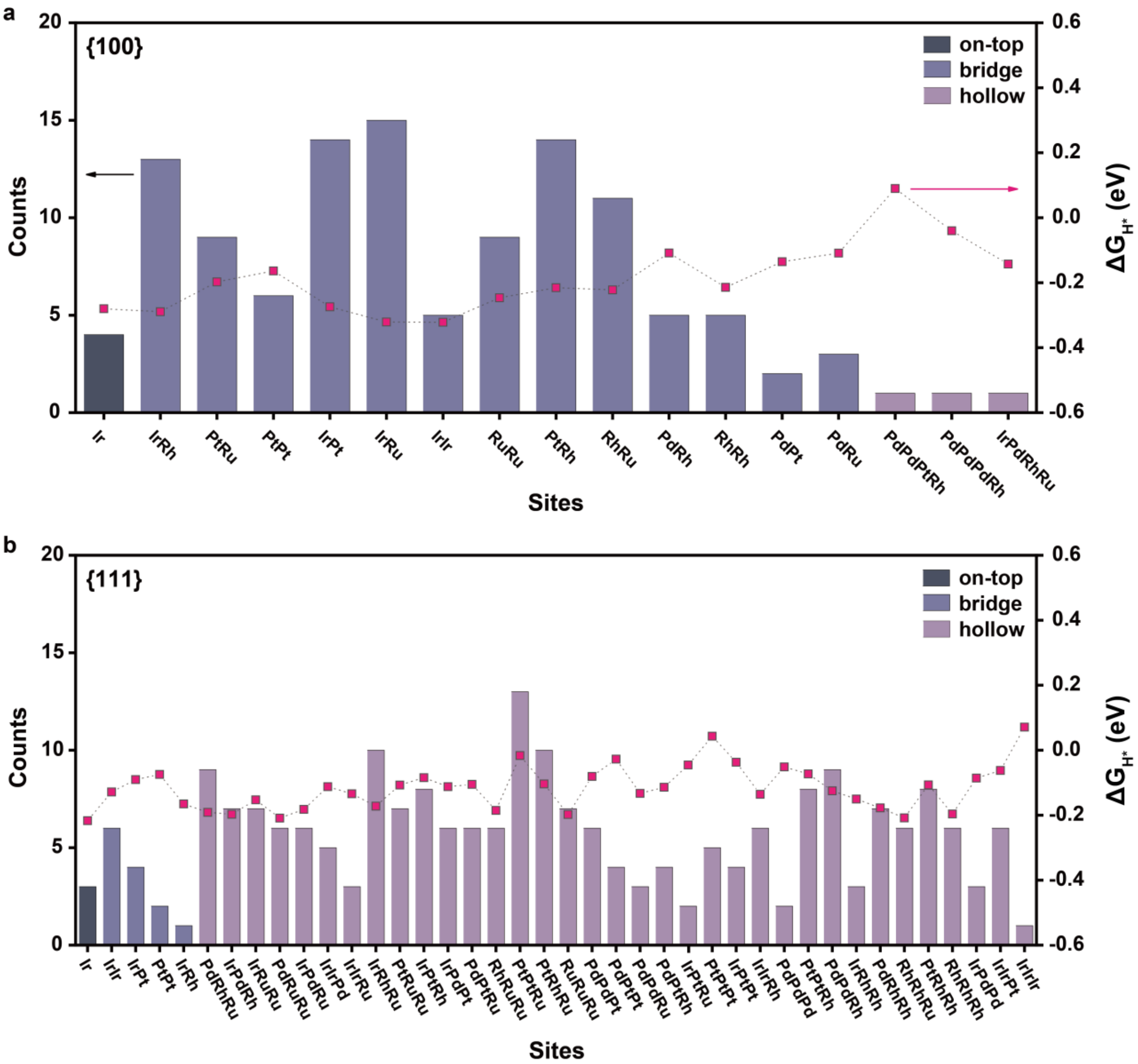

Figure S26. Distribution of hydrogen adsorption sites and their corresponding average ΔG_{H^*} values on the (a) {100} and (b) {111} facets of $Pd_{0.2}Pt_{0.2}Ir_{0.2}Ru_{0.2}Rh_{0.2}$ HEA surfaces. The bars represent the number of adsorption configurations (counts) for each site, corresponding to the left y-axis, while the square data points represent the corresponding H^* adsorption free energy (ΔG_{H^*}), referenced to the right y-axis.

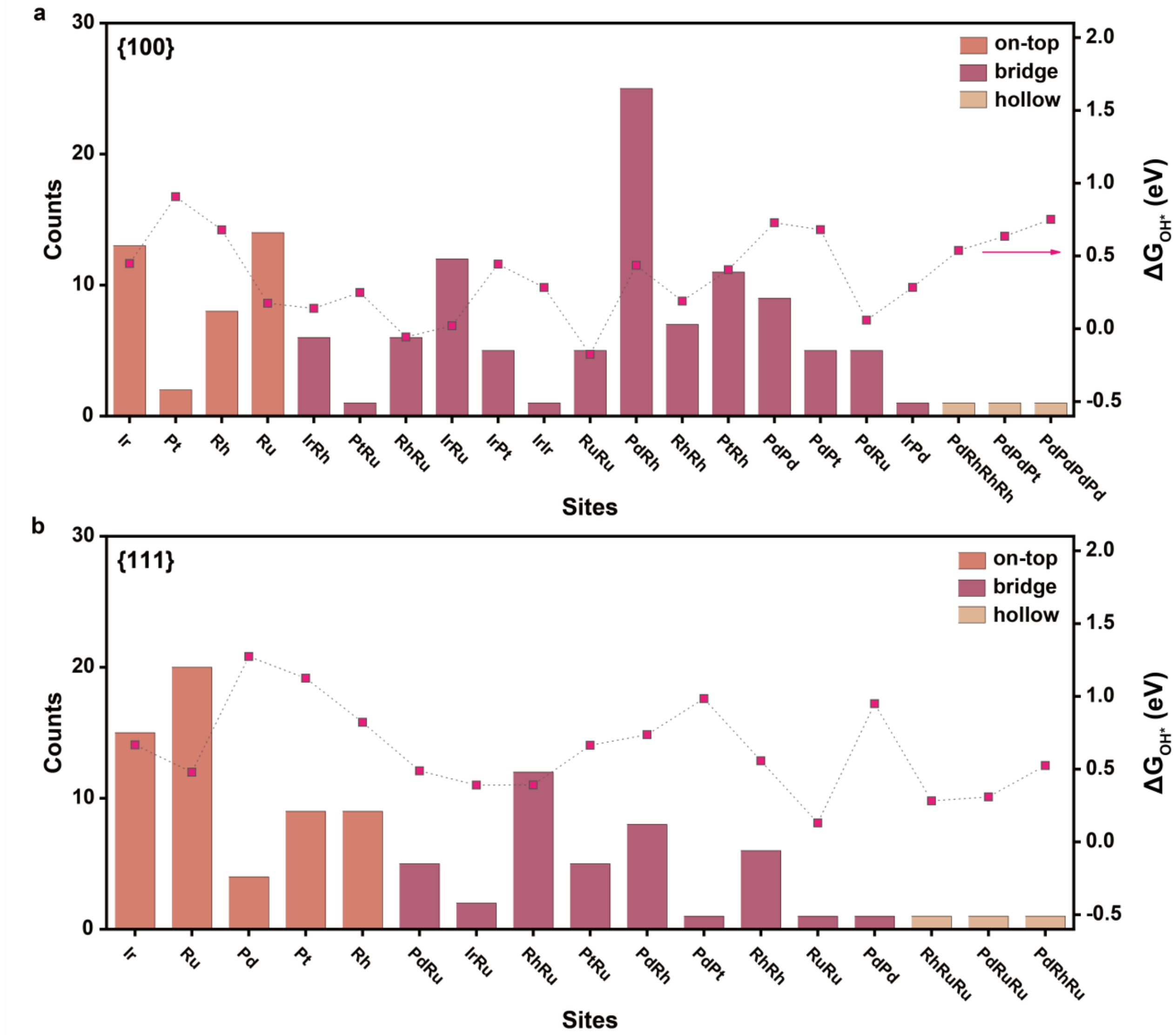

Figure S27. Distribution of hydroxyl adsorption sites and their corresponding average ΔG_{OH*} values on the (a) {100}, and (b) {111} facets of $Pd_{0.2}Pt_{0.2}Ir_{0.2}Ru_{0.2}Rh_{0.2}$ HEA surfaces. The bars represent the number of adsorption configurations (counts) for each site, corresponding to the left y-axis, while the square data points represent the corresponding hydroxyl adsorption free energy (ΔG_{OH*}), referenced to the right y-axis.

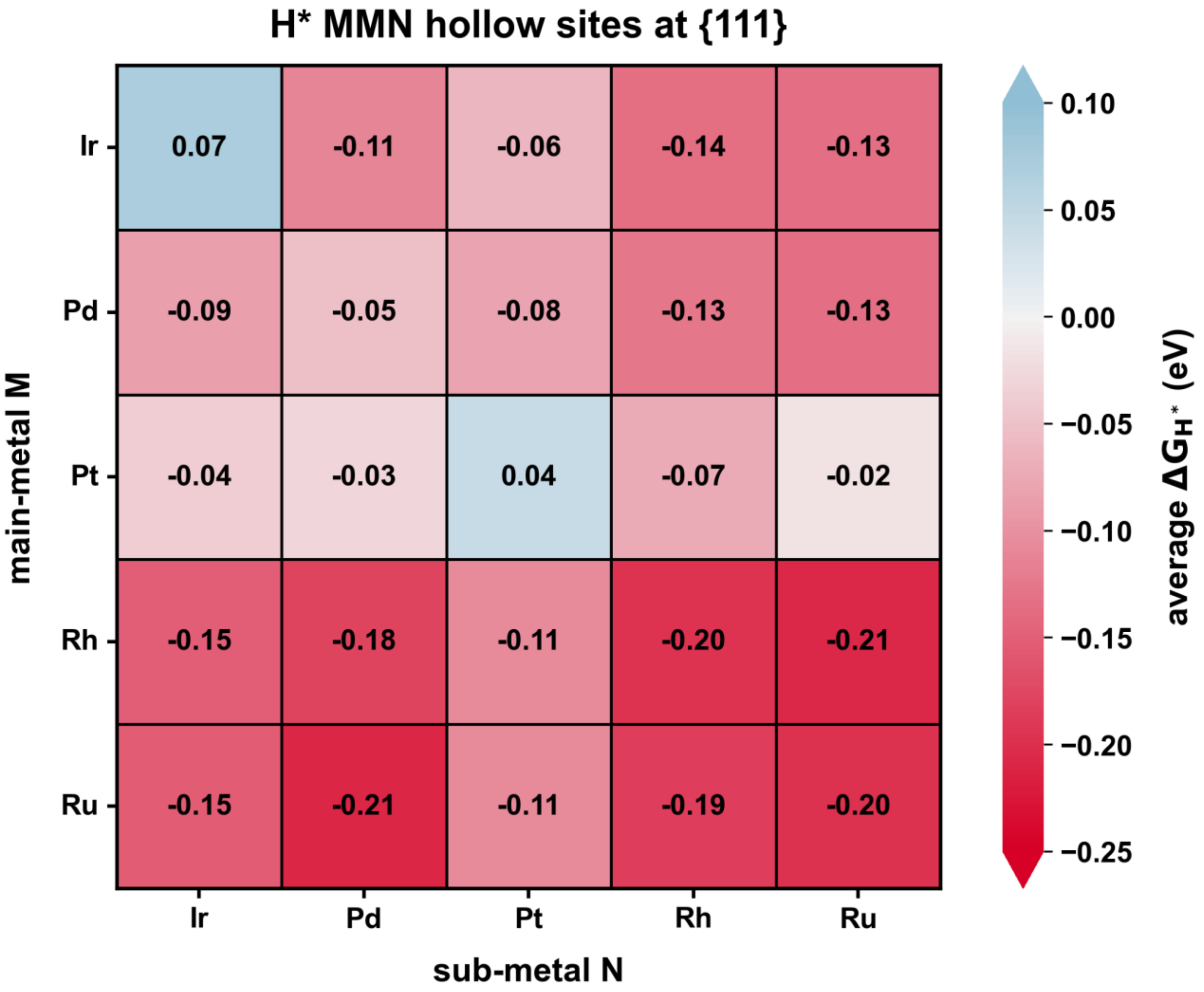

Figure S28. Calculated average ΔG_{H*} at specific MMN hollow sites on the {111} facet of $Pd_{0.2}Pt_{0.2}Ir_{0.2}Ru_{0.2}Rh_{0.2}$ HEA surfaces. The MMN ensemble consists of three surface atoms forming a hollow site, including two identical main-metal atoms (M, y-axis) and one sub-metal atom (N, x-axis). The color scale represents the adsorption strength in eV, with darker red indicating stronger (more negative) hydrogen adsorption.

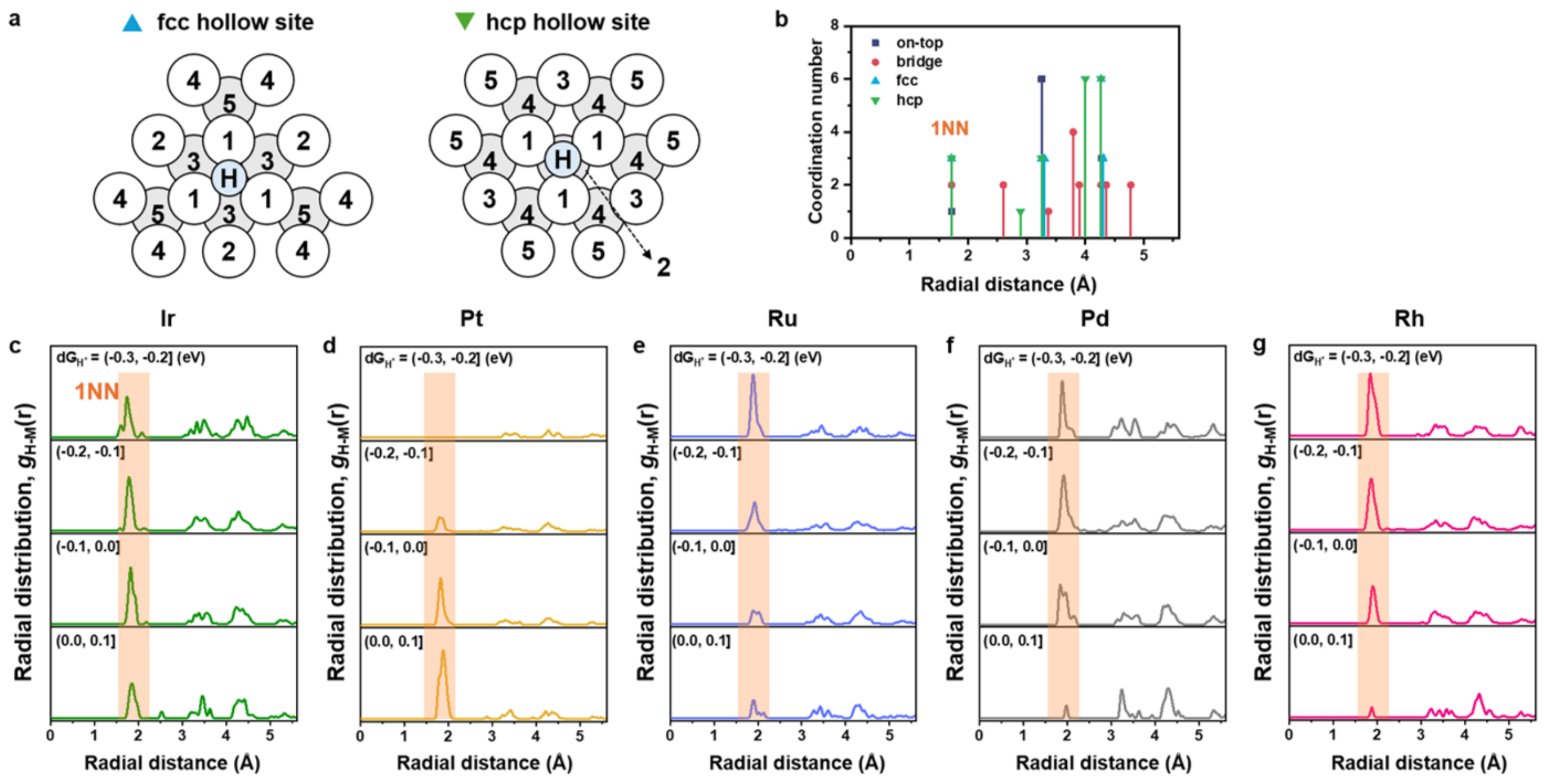

Figure S29. Element-resolved radial distribution function analysis of H^* adsorption on the equimolar PdPtIrRuRh HEA {111} surface. (a) Schematic atomic structure used for the radial distribution analysis, with representative fcc- and hcp-type hollow sites distinguished according to the local stacking sequence of the underlying atomic layers (ABC and ABA, respectively). (b) Reference coordination numbers (CNs) of the first five coordination shells around H^* located at on-top, bridge, fcc-hollow, and hcp-hollow sites on an ideal FCC {111} surface. Only the first five coordination shells are considered because contributions from more distant shells become negligible.[19, 20] (c–g) Ensemble-averaged element-resolved radial distribution functions, $g_{H\text{-}M}(r)$, for (c) Ir, (d) Pt, (e) Ru, (f) Pd, and (g) Rh around H^*, categorized according to the corresponding ΔG_{H*} ranges. Parentheses indicate that the boundary value is excluded from the adsorption-energy interval, whereas square brackets indicate that the boundary value is included. For example, $\Delta G_{H*} = (-0.3, -0.2]$ eV denotes $-0.3 < \Delta G_{H*} \leq -0.2$ eV. The shaded region denotes the first-nearest-neighbor (1NN) coordination shell. Because H^* predominantly relaxes to hollow sites on the HEA {111} surface, the 1NN distributions mainly reflect the three metal atoms directly constituting the local hollow adsorption ensemble. The most pronounced adsorption-energy-dependent variations occur within this region, indicating that the identities of the directly coordinated metals strongly influence H^* binding. A higher Pt contribution is generally

associated with weaker H^* adsorption and shifts ΔG_{H^*} toward more thermoneutral values, whereas increased Pd, Rh, and Ru contributions are associated with stronger H^* binding. These trends indicate that Pd, Rh, and Ru can participate in mixed-metal hollow ensembles that stabilize H^*, while Pt moderates the adsorption strength and broadens the accessible range of H-binding environments. Ir exhibits a comparatively weaker dependence on ΔG_{H^*} and remains distributed across a broad range of adsorption energies, suggesting that its contribution is more sensitive to the surrounding multimetallic environment. Overall, the RDF analysis shows that H^* adsorption on the HEA {111} surface is governed by cooperative mixed-metal hollow ensembles rather than by isolated single-metal adsorption centers. The short-distance feature at approximately 1.6–1.7 Å in the stronger-binding regime is attributed to a minor population of H^* adsorbed at Ir on-top sites.

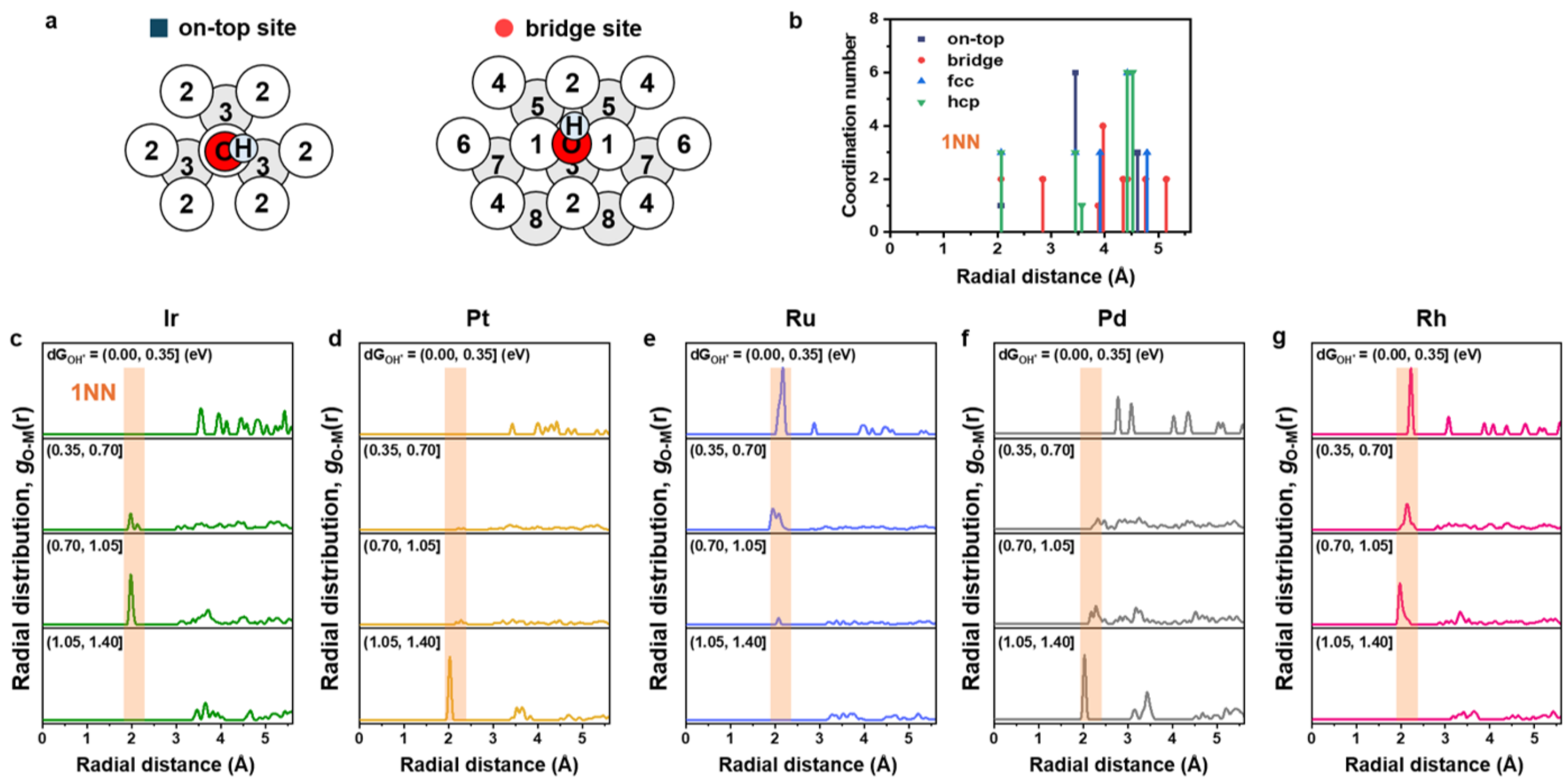

Figure S30. Element-resolved radial distribution function analysis of OH^* adsorption on the equimolar PdPtIrRuRh HEA {111 surface. (a) Schematic atomic structure used for the radial distribution analysis, with representative on-top and bridge sites indicated. (b) Reference coordination numbers (CNs) of the first five coordination shells around OH^* located at on-top, bridge, fcc-hollow, and hcp-hollow sites on an ideal FCC {111} surface. (c–g) Ensemble-averaged element-resolved radial distribution functions, $g_{O\text{-}M}(r)$, for (c) Ir, (d) Pt, (e) Ru, (f) Pd, and (g) Rh around OH^*, categorized according to the corresponding ΔG_{OH^*} ranges. Parentheses indicate that the boundary value is excluded from the adsorption-energy interval, whereas square brackets indicate that the boundary value is included. For example, ΔG_{OH^*} = (0.00, 0.35] eV denotes $0.00 < \Delta G_{OH^*} \leq 0.35$ eV. The shaded region denotes the 1NN coordination shell. Unlike H^*, which predominantly occupies hollow sites, OH^* adsorption on the HEA {111} surface mainly occurs at on-top and bridge sites; thus, the 1NN distributions primarily reflect the metal atoms directly coordinated to the O atom of OH^*. The most pronounced adsorption-energy-dependent variations occur within this region, indicating that the identities of the directly coordinated metals strongly influence OH^* binding. In the stronger OH-binding regime, Ru and Rh exhibit enhanced 1NN contributions, showing that their direct participation in the local adsorption environment favors stronger OH^* interactions. In contrast, Pt and Pd are preferentially associated with

weaker OH^* adsorption and exhibit little direct coordination within the strongest-binding configurations. Ir displays an intermediate dependence on ΔG_{OH*}, remaining distributed across a broader range of adsorption energies. Notably, although Pd contributes substantially to H^* coordination on the {111} surface, it shows a much weaker tendency to directly coordinate strongly bound OH*, highlighting distinct elemental preferences toward H^* and OH^* adsorption. Pd nevertheless remains present in more distant coordination shells, suggesting that it may contribute indirectly through the surrounding multimetallic environment rather than acting as a primary OH^*-binding center. Overall, the RDF analysis indicates that OH^* adsorption on the HEA {111} surface is governed by both the identity of the directly coordinated metals and the broader multimetallic environment, with Ru and Rh favoring stronger OH^* binding and Pt and Pd favoring weaker OH^* interactions.

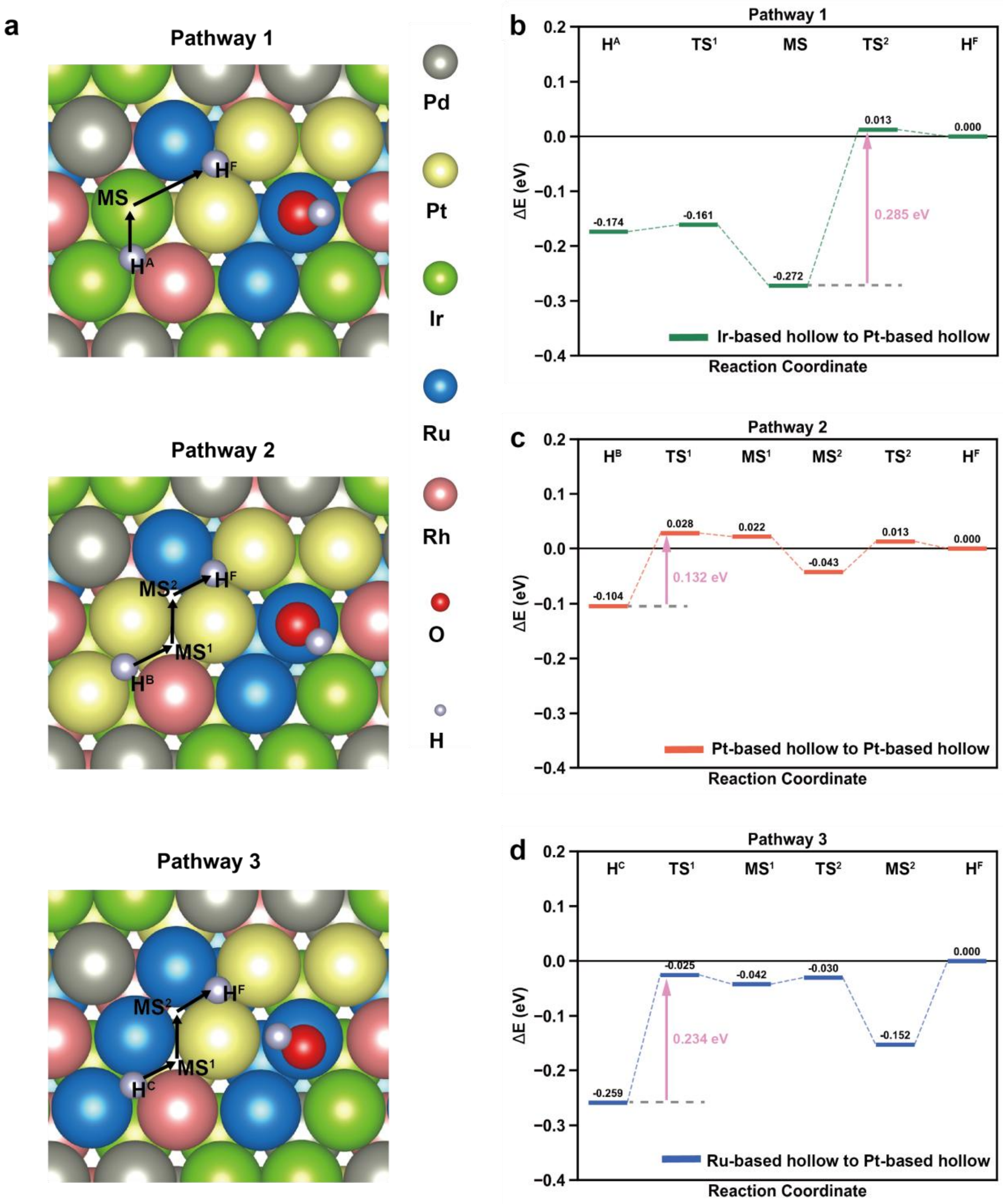

Figure S31. Relative energy profiles of H^* diffusion pathways on the {111} facet of $Pd_{0.2}Pt_{0.2}Ir_{0.2}Ru_{0.2}Rh_{0.2}$ HEA surfaces. (a) Three representative H^* diffusion pathways toward the same Pt-based hollow active site (H^F). (b–d) Energy profiles for H^* migration from (b) an Ir-based hollow site (H^A), (c) a Pt-based hollow site (H^B), and (d) a Ru-based hollow site (H^C). The diffusion distance and orientation were kept consistent to isolate the compositional effect.

Table S1. Estimated average shell thickness (n, atomic layers) and corresponding shell atomic percentages (at%) for core-shell nanocrystals. The ideal shell at% values were calculated based on n and the measured sizes of the Pd seeds, whereas the experimental shell at% values were obtained from ICP-OES. These estimations are consistent with the HAADF-STEM observations in Figure 2, which indicates an HEA shell thickness of approximately four to five atomic layers.

**Sample**	**Number of atomic layers**	**Ideal at% of shell**	**Experimental at% of shell**
Pd@$Pd_{0.2}Pt_{0.2}Ir_{0.2}Ru_{0.2}Rh_{0.2}$ nanocubes	4	19.8	21.6
Pd@$Pd_{0.2}Pt_{0.2}Ir_{0.2}Ru_{0.2}Rh_{0.2}$ nanooctahedra	4	20.6	23.2
Pd@$Pd_{0.2}Pt_{0.2}Ir_{0.2}Ru_{0.2}Rh_{0.2}$ nanoicosahedra	4	17.5	19.3
Pd@$Pt_{0.33}Ir_{0.33}Ru_{0.33}$ nanooctahedra	4	20.6	22.8
Pd@$Ir_{0.5}Ru_{0.5}$ nanooctahedra	4	20.6	20.4
Pd@$Pt_{0.5}Ru_{0.5}$ nanooctahedra	4	20.6	20.0
Pd@Pt nanooctahedra	4	20.6	17.4
Pd@Ru nanooctahedra	4	20.6	17.1

Table S2. Shell compositions of the core-shell nanocrystals determined by ICP-OES analysis. The values are reported as elemental ratios of the HEA shells.

	**Composition of shell (at%)**				
**Sample**	**Pd**	**Pt**	**Ir**	**Ru**	**Rh**
Pd@$Pd_{0.2}Pt_{0.2}Ir_{0.2}Ru_{0.2}Rh_{0.2}$ nanocubes	21.1	19.7	20.1	18.3	20.8
Pd@$Pd_{0.2}Pt_{0.2}Ir_{0.2}Ru_{0.2}Rh_{0.2}$ nanooctahedra	17.2	16.4	24.0	21.9	20.5
Pd@$Pd_{0.2}Pt_{0.2}Ir_{0.2}Ru_{0.2}Rh_{0.2}$ nanoicosahedra	18.3	20.4	22.3	18.8	20.2
Pd@$Pt_{0.33}Ir_{0.33}Ru_{0.33}$ nanooctahedra	--	32.2	34.8	33.1	--
Pd@$Ir_{0.5}Ru_{0.5}$ nanooctahedra	--	--	48.6	51.4	--
Pd@$Pt_{0.5}Ru_{0.5}$ nanooctahedra	--	49.9	--	50.1	--
Pd@Pt nanooctahedra	--	100.0	--	--	--
Pd@Ru nanooctahedra	--	--	--	100.0	--

Table S3. Surface chemical states and corresponding fitting parameters of $Pd@Pd_{0.2}Pt_{0.2}Ir_{0.2}Ru_{0.2}Rh_{0.2}$ core-shell nanooctahedra derived from XPS spectra. The data were extracted from XPS spectra fitted according to the procedure described in the Materials and Methods section.

Element	Name	Position	FWHM	Area	%Area
Pd	$Pd^{0}3d_{3/2}$	340.44	1.18	14659.68	35.53
	$Pd^{0}3d_{5/2}$	335.15	1.18	21978.53	53.27
	$Pd^{2+}3d_{3/2}$	342.14	1.85	1848.17	4.48
	$Pd^{2+}3d_{5/2}$	336.64	1.85	2770.88	6.72
Pt	$Pt^{0}4f_{5/2}$	74.26	1.25	3243.59	37.42
	$Pt^{0}4f_{7/2}$	70.92	1.25	4324.78	49.89
	$Pt^{2+}4f_{5/2}$	75.28	1.28	471.48	5.44
	$Pt^{2+}4f_{7/2}$	72.08	1.28	628.63	7.25
Ir	$Ir^{0}4f_{5/2}$	63.26	1.06	4244.71	34.08
	$Ir^{0}4f_{7/2}$	60.29	1.06	5659.61	45.45
	$Ir^{4+}4f_{5/2}$	64.19	1.24	1092.51	8.77
	$Ir^{4+}4f_{7/2}$	61.31	1.24	1456.68	11.70
Ru	$Ru^{0}3p_{1/2}$	483.65	2.94	1963.94	29.04
	$Ru^{0}3p_{3/2}$	461.18	2.94	3927.87	58.07
	$Ru^{4+}3p_{1/2}$	485.50	3.54	290.56	4.30
	$Ru^{4+}3p_{3/2}$	463.42	3.54	581.13	8.59
Rh	$Rh^{0}3d_{3/2}$	311.58	1.35	3719.01	28.35
	$Rh^{0}3d_{5/2}$	306.93	1.35	5575.72	42.51
	$Rh^{2+}3d_{3/2}$	313.50	2.49	1529.42	11.66
	$Rh^{2+}3d_{5/2}$	308.60	2.49	2292.99	17.48

Table S4. Area percentages of different chemical states for each element in $Pd@Pd_{0.2}Pt_{0.2}Ir_{0.2}Ru_{0.2}Rh_{0.2}$ core-shell nanooctahedra at various applied potentials, derived from *in situ* AP-XPS spectra fitting. The area percentages were obtained from *in situ* AP-XPS spectra fitted according to the procedure described in the Materials and Methods section. These data reveal the potential-dependent evolution of surface oxidation states for each element, enabling evaluation of valence-state changes under electrochemical conditions.

		Area percentages (%)			
Orbital	Chemical state	OCP	+0.05 V	+0.60 V	Return to OCP
Pd 3d	Pd^0	71.71	71.59	65.97	69.83
	Pd^{2+}	28.29	28.41	34.03	30.17
Pt 4f	Pt^0	67.79	66.97	66.51	67.69
	Pt^{2+}	32.21	33.03	33.49	32.31
Ir 4f	Ir^0	69.54	68.74	66.44	68.31
	Ir^{4+}	30.46	31.26	33.56	31.69
Ru 3d	Ru^0	59.41	54.57	48.29	51.68
	Ru^{4+}	40.59	45.43	51.71	48.32
Rh 3d	Rh^0	61.42	59.61	57.40	59.16
	Rh^{3+}	22.74	24.62	26.89	24.98
O 1s	M–O	13.07	13.46	15.85	14.46
	-OH	20.35	19.92	19.10	19.91
	H_2O (ads./liq.)	18.17	17.76	17.51	17.89

Table S5. Summary of hydrogen adsorption characteristics on both the {100} and {111} facets of $Pd_{0.2}Pt_{0.2}Ir_{0.2}Ru_{0.2}Rh_{0.2}$ HEA surfaces, including all possible adsorption sites, their relative populations among surface sites, and the corresponding average hydrogen adsorption energies (ΔG_{H*}).

Facet	SITE	[a]FNN	Counts	[b]Population(%)	ΔG_{H*} (eV)	[c]s (eV)
{100}	on-top / 3.4%	Ir	4	3.4	-0.280	0.027
	bridge / 94.1%	IrRh	13	11.0	-0.289	0.039
		PtRu	9	7.6	-0.197	0.040
		PtPt	6	5.1	-0.164	0.037
		IrPt	14	11.9	-0.274	0.051
		IrRu	15	12.7	-0.321	0.028
		IrIr	5	4.2	-0.322	0.019
		RuRu	9	7.6	-0.247	0.034
		PtRh	14	11.9	-0.215	0.030
		RhRu	11	9.3	-0.222	0.044
		PdRh	5	4.2	-0.109	0.060
		RhRh	5	4.2	-0.214	0.024
		PdPt	2	1.7	-0.136	0.067
		PdRu	3	2.5	-0.109	0.081
	hollow / 2.5%	PdPdPtRh	1	0.8	0.090	-
		PdPdPdRh	1	0.8	-0.040	-
		IrPdRhRu	1	0.8	-0.143	-
{111}	on-top / 1.3%	Ir	3	1.3	-0.217	0.063
	bridge / 5.8%	IrIr	6	2.7	-0.129	0.035
		IrPt	4	1.8	-0.091	0.075
		PtPt	2	0.9	-0.075	0.056
		IrRh	1	0.4	-0.166	-
	hollow / 92.9%	PdRhRu	9	4.0	-0.191	0.044
		IrPdRh	7	3.1	-0.197	0.052
		IrRuRu	7	3.1	-0.153	0.052
		PdRuRu	6	2.7	-0.209	0.043
		IrPdRu	6	2.7	-0.182	0.014
		IrIrPd	5	2.2	-0.112	0.042
		IrIrRu	3	1.3	-0.134	0.011

		IrRhRu	10	4.4	-0.172	0.063
		PtRuRu	7	3.1	-0.107	0.034
		IrPtRh	8	3.6	-0.085	0.038
		IrPdPt	6	2.7	-0.112	0.041
		PdPtRu	6	2.7	-0.105	0.023
		RhRuRu	6	2.7	-0.185	0.041
		PtPtRu	13	5.8	-0.016	0.038
		PtRhRu	10	4.4	-0.104	0.042
		RuRuRu	7	3.1	-0.198	0.039
		PdPdPt	6	2.7	-0.081	0.028
		PdPtPt	4	1.8	-0.027	0.041
		PdPdRu	3	1.3	-0.133	0.030
		PdPtRh	4	1.8	-0.114	0.012
		IrPtRu	2	0.9	-0.046	0.000
		PtPtPt	5	2.2	0.043	0.017
		IrPtPt	4	1.8	-0.037	0.063
		IrIrRh	6	2.7	-0.136	0.062
		PdPdPd	2	0.9	-0.051	0.041
		PtPtRh	8	3.6	-0.073	0.065
		PdPdRh	9	4.0	-0.125	0.025
		IrRhRh	3	1.3	-0.150	0.049
		PdRhRh	7	3.1	-0.178	0.038
		RhRhRu	6	2.7	-0.208	0.048
		PtRhRh	8	3.6	-0.107	0.036
		RhRhRh	6	2.7	-0.196	0.032
		IrPdPd	3	1.3	-0.086	0.023
		IrIrPt	6	2.7	-0.063	0.051
		IrIrIr	1	0.4	0.071	-

[a] FNN: The first nearest neighbors to H^*.

[b] Population: Relative population among all sites of each facet.

[c] S: Sample standard deviation.

Table S6. Summary of hydroxyl adsorption characteristics on the {100} and {111} facets of $Pd_{0.2}Pt_{0.2}Ir_{0.2}Ru_{0.2}Rh_{0.2}$ HEA surfaces, including all possible adsorption sites, their relative populations among surface sites, and the corresponding average hydroxyl adsorption energies (ΔG_{OH*}).

Facet	SITE	[a]FNN	Counts	[b]Population(%)	ΔG_{OH*} (eV)	[c]s (eV)
{100}	on-top / 26.6%	Ir	13	9.4	0.448	0.073
		Pt	2	1.4	0.907	0.094
		Rh	8	5.8	0.679	0.062
		Ru	14	10.1	0.175	0.084
	bridge / 71.2%	IrRh	6	4.3	0.140	0.064
		PtRu	1	0.7	0.249	-
		RhRu	6	4.3	-0.057	0.082
		IrRu	12	8.6	0.021	0.064
		IrPt	5	3.6	0.444	0.050
		IrIr	1	0.7	0.283	-
		RuRu	5	3.6	-0.176	0.021
		PdRh	25	18.0	0.436	0.066
		RhRh	7	5.0	0.190	0.057
		PtRh	11	7.9	0.405	0.093
		PdPd	9	6.5	0.728	0.056
		PdPt	5	3.6	0.681	0.074
		PdRu	5	3.6	0.059	0.053
		IrPd	1	0.7	0.284	-
	hollow / 2.2%	PdRhRhRh	1	0.7	0.538	-
		PdPdPt	1	0.7	0.636	-
		PdPdPdPd	1	0.7	0.751	-
{111}	on-top / 56.4%	Ir	15	14.9	0.667	0.082
		Ru	20	19.8	0.479	0.070
		Pd	4	4.0	1.274	0.042
		Pt	9	8.9	1.125	0.050
		Rh	9	8.9	0.821	0.079
	bridge / 40.6%	PdRu	5	5.0	0.488	0.057
		IrRu	2	2.0	0.391	0.050
		RhRu	12	11.9	0.391	0.069

		PtRu	5	5.0	0.663	0.066
		PdRh	8	7.9	0.737	0.116
		PdPt	1	1.0	0.984	-
		RhRh	6	5.9	0.557	0.083
		RuRu	1	1.0	0.130	-
		PdPd	1	1.0	0.950	-
	hollow / 3.0%	RhRuRu	1	1.0	0.282	-
		PdRuRu	1	1.0	0.309	-
		PdRhRu	1	1.0	0.525	-

[a] FNN: The first nearest neighbors to OH^*.

[b] Population: Relative population among all sites of each facet.

[c] S: Sample standard deviation.

References

(1) Jin, M.; Liu, H.; Zhang, H.; Xie, Z.; Liu, J.; Xia, Y. Synthesis of Pd nanocrystals enclosed by {100} facets and with sizes <10 nm for application in CO oxidation. *Nano Res.* **2011**, *4* (1), 83-91.

(2) Peng, H.-C.; Xie, S.; Park, J.; Xia, X.; Xia, Y. Quantitative Analysis of the Coverage Density of Br– Ions on Pd{100} Facets and Its Role in Controlling the Shape of Pd Nanocrystals. *J. Am. Chem. Soc.* **2013**, *135* (10), 3780-3783.

(3) Jin, M.; Zhang, H.; Xie, Z.; Xia, Y. Palladium nanocrystals enclosed by {100} and {111} facets in controlled proportions and their catalytic activities for formic acid oxidation. *Energy Environ. Sci.* **2012**, *5* (4), 6352-6357.

(4) Lv, T.; Yang, X.; Zheng, Y.; Huang, H.; Zhang, L.; Tao, J.; Pan, L.; Xia, Y. Controlling the Growth of Au on Icosahedral Seeds of Pd by Manipulating the Reduction Kinetics. *J. Phys. Chem. C* **2016**, *120* (37), 20768-20774.

(5) Baletto, F.; Ferrando, R. Structural properties of nanoclusters: Energetic, thermodynamic, and kinetic effects. *Rev. Mod. Phys.* **2005**, *77* (1), 371-423.

(6) Teo, B. K.; Sloane, N. Magic numbers in polygonal and polyhedral clusters. *Inorg. Chem.* **1985**, *24* (26), 4545-4558.

(7) Kaatz, F. H.; Bultheel, A. Informational thermodynamic model for nanostructures. *J. Math. Chem.* **2014**, *52* (6), 1563-1575.

(8) Xie, S.; Choi, S.-I.; Lu, N.; Roling, L. T.; Herron, J. A.; Zhang, L.; Park, J.; Wang, J.; Kim, M. J.; Xie, Z.; et al. Atomic Layer-by-Layer Deposition of Pt on Pd Nanocubes for Catalysts with Enhanced Activity and Durability toward Oxygen Reduction. *Nano Lett.* **2014**, *14* (6), 3570-3576.

(9) Safo, I. A.; Dosche, C.; Oezaslan, M. TEM, FTIR and Electrochemistry Study: Desorption of PVP from Pt Nanocubes. *Z. Phys. Chem.* **2018**, *232* (9-11), 1319-1333.

(10) Morales, D. M.; Risch, M. Seven steps to reliable cyclic voltammetry measurements for the determination of double layer capacitance. *J. Phys. Energy* **2021**, *3* (3).

(11) McCrory, C. C. L.; Jung, S.; Peters, J. C.; Jaramillo, T. F. Benchmarking Heterogeneous Electrocatalysts for the Oxygen Evolution Reaction. *J. Am. Chem. Soc.* **2013**, *135* (45), 16977-16987.

(12) McCrory, C. C. L.; Jung, S.; Ferrer, I. M.; Chatman, S. M.; Peters, J. C.; Jaramillo, T. F. Benchmarking Hydrogen Evolving Reaction and Oxygen Evolving Reaction Electrocatalysts for Solar Water Splitting Devices. *J. Am. Chem. Soc.* **2015**, *137* (13), 4347-4357.

(13) Ramaswamy, N.; Ghoshal, S.; Bates, M. K.; Jia, Q.; Li, J.; Mukerjee, S. Hydrogen oxidation reaction in alkaline media: Relationship between electrocatalysis and electrochemical double-layer structure. *Nano Energy* **2017**, *41*, 765-771.

(14) Huang, M.; Yang, H.; Xia, X.; Peng, C. Highly active and robust Ir-Ru electrocatalyst for alkaline HER/HOR: Combined electronic and oxophilic effect. *Appl. Catal. B: Environ. and Energy* **2024**, *358*, 124422.

(15) Chastain, J.; King Jr, R. C. Handbook of X-ray photoelectron spectroscopy. *Perkin-Elmer Corporation* **1992**.

(16) Nørskov, J. K.; Bligaard, T.; Logadottir, A.; Kitchin, J. R.; Chen, J. G.; Pandelov, S.; Stimming, U. Trends in the Exchange Current for Hydrogen Evolution. *J. Electrochem. Soc.* **2005**, *152* (3), J23.

(17) Man, I. C.; Su, H.-Y.; Calle-Vallejo, F.; Hansen, H. A.; Martínez, J. I.; Inoglu, N. G.; Kitchin, J.; Jaramillo, T. F.; Nørskov, J. K.; Rossmeisl, J. Universality in Oxygen Evolution Electrocatalysis on Oxide Surfaces. *ChemCatChem* **2011**, *3* (7), 1159-1165.

(18) Haynes, W. M. *CRC Handbook of Chemistry and Physics, 93rd Edition*; CRC Press, 2012.

(19) Batchelor, T. A. A.; Pedersen, J. K.; Winther, S. H.; Castelli, I. E.; Jacobsen, K. W.; Rossmeisl, J. High-Entropy Alloys as a Discovery Platform for Electrocatalysis. *Joule* **2019**, *3* (3), 834-845.

(20) Zhou, Q.; Shou, H.; Qiao, S.; Cao, Y.; Zhang, P.; Wei, S.; Chen, S.; Wu, X.; Song, L. Analyzing the Active Site and Predicting the Overall Activity of Alloy Catalysts. *J. Am. Chem. Soc.* **2024**, *146* (22), 15167-15175.

(21) Clausen, C. M.; Batchelor, T. A. A.; Pedersen, J. K.; Rossmeisl, J. What Atomic Positions Determines Reactivity of a Surface? Long-Range, Directional Ligand Effects in Metallic Alloys. *Adv. Sci.* **2021**, *8* (9), 2003357.

(22) Pedersen, J. K.; Clausen, C. M.; Krysiak, O. A.; Xiao, B.; Batchelor, T. A. A.; Löffler, T.; Mints, V. A.; Banko, L.; Arenz, M.; Savan, A.; et al. Bayesian Optimization of High-Entropy Alloy Compositions for Electrocatalytic Oxygen Reduction. *Angew. Chem. Int. Ed.* **2021**, *60* (45), 24144-24152.

(23) Liu, Y.-H.; Hsieh, C.-J.; Hsu, L.-C.; Lin, K.-H.; Hsiao, Y.-C.; Chi, C.-C.; Lin, J.-T.; Chang, C.-W.; Lin, S.-C.; Wu, C.-Y.; et al. Toward controllable and predictable synthesis of high-entropy alloy nanocrystals. *Sci. Adv.* **2023**, *9* (19), eadf9931.
